# Optimization of a “bump-and-hole” approach to allele-selective BET bromodomain inhibition†
†Electronic supplementary information (ESI) available: Supplemental results (Fig. S1–S13 and Tables S1–S10). Crystallography data collection and refinement statistics. Experimental section. Detailed compound synthesis and characterisation. Supplemental references. See DOI: 10.1039/c7sc02536j


**DOI:** 10.1039/c7sc02536j

**Published:** 2018-01-24

**Authors:** A. C. Runcie, M. Zengerle, K.-H. Chan, A. Testa, L. van Beurden, M. G. J. Baud, O. Epemolu, L. C. J. Ellis, K. D. Read, V. Coulthard, A. Brien, A. Ciulli

**Affiliations:** a Division of Biological Chemistry and Drug Discovery , School of Life Sciences , University of Dundee , Dundee , Scotland , UK . Email: a.ciulli@dundee.ac.uk; b Reach Separations Ltd , BioCity Nottingham , Nottingham , UK

## Abstract

Allele-specific chemical genetics enables selective inhibition within families of highly-conserved proteins. The four BET (bromodomain & extra-terminal domain) proteins – BRD2, BRD3, BRD4 and BRDT bind acetylated chromatin *via* their bromodomains and regulate processes such as cell proliferation and inflammation. BET bromodomains are of particular interest, as they are attractive therapeutic targets but existing inhibitors are pan-selective. We previously established a bump-&-hole system for the BET bromodomains, pairing a leucine/alanine mutation with an ethyl-derived analogue of an established benzodiazepine scaffold. Here we optimize upon this system with the introduction of a more conservative and less disruptive leucine/valine mutation. Extensive structure–activity-relationships of diverse benzodiazepine analogues guided the development of potent, mutant-selective inhibitors with desirable physiochemical properties. The active enantiomer of our best compound – 9-ME-1 – shows ∼200 nM potency, >100-fold selectivity for the L/V mutant over wild-type and excellent DMPK properties. Through a variety of *in vitro* and cellular assays we validate the capabilities of our optimized system, and then utilize it to compare the relative importance of the first and second bromodomains to chromatin binding. These experiments confirm the primacy of the first bromodomain in all BET proteins, but also significant variation in the importance of the second bromodomain. We also show that, despite having a minor role in chromatin recognition, BRD4 BD2 is still essential for gene expression, likely through the recruitment of non-histone proteins. The disclosed inhibitor:mutant pair provides a powerful tool for future cellular and *in vivo* target validation studies.

## Abstract 2

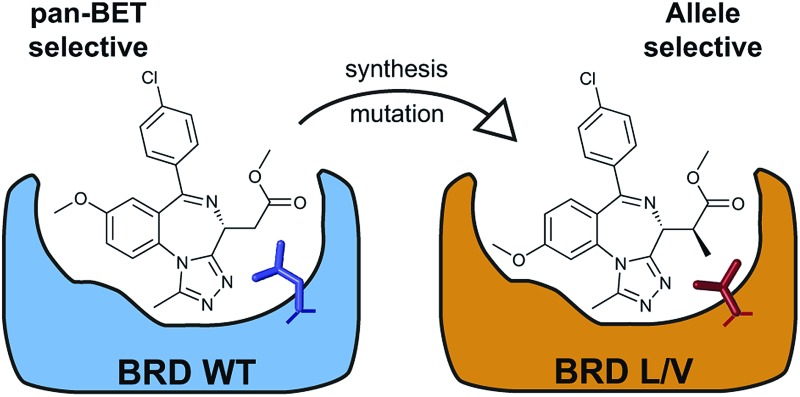
Allele-specific chemical genetics enables selective inhibition within families of highly-conserved proteins.

## Introduction

Chemical probes are biologically-active small-molecules (typically inhibitors) that are used to investigate the importance and functions of proteins.^1^–^3^ The use of chemical probes and observation of the resulting phenotypes in this fashion is known as chemical genetics. Although possessing various advantages over classical genetics (such as gene knockouts) chemical genetics requires that any probes used have a well-defined mode of action and high-selectivity for their target proteins. In cases where target proteins are not structurally distinct enough for the development of selective probes more advanced techniques are needed.

The ‘bump-&-hole’ system is a way of engineering selective inhibition of structurally conserved proteins through the generation of orthogonal protein:ligand pairs.^4^ In this system existing small-molecule inhibitors, showing high affinity and desirable DMPK properties, are modified to include a steric ‘bump’ that weakens or abolishes binding to the target wild-type proteins. Simultaneously, a reciprocal mutation is introduced to the target, replacing a large amino acid residue with a smaller one to create a ‘hole’ that can accommodate the bumped ligand. Using this approach one can take a pan-selective inhibitor that binds multiple structurally-related proteins and generate a bumped ligand that will only inhibit a target protein that has been mutated to contain a ‘hole’. This system allows the specific inhibition of multiple proteins without the costly design of multiple target-specific chemical probes, and takes advantage of existing chemical tools to bypass the discovery of a high-quality chemical scaffold. Such engineered selectivity has successfully been applied to protein kinases and ATP-competitive inhibitors^5^ and FKBP-targeting chemical dimerizers.^6^,^7^ The bump-&-hole approach has not previously been applied to any inhibitor of epigenetic proteins, but the use of mutant enzymes and modified co-factors has been used on a number of epigenetic enzymes for target identification.^1^,^4^,^8^,^9^

In previous work we have explored the potential for establishing a bump-&-hole system targeting the bromodomains of the BET (bromo and extra-terminal) protein family.^10^,^11^ These four human proteins – BRD2, BRD3, BRD4 and BRDT – each contain two tandem bromodomains that bind acetylated lysine residues in histone tails, leading to the recruitment of multi-protein complexes to chromatin.^12^,^13^ Through this function the BET proteins play a significant role in controlling transcription and regulating gene expression.^14^–^16^ The BET proteins regulate proliferation, the cell-cycle and cell differentiation in a wide array of contexts and they have been associated with many disease states such as cancer, inflammation, HIV infection and neurological disorders.^17^

**Chart 1:**
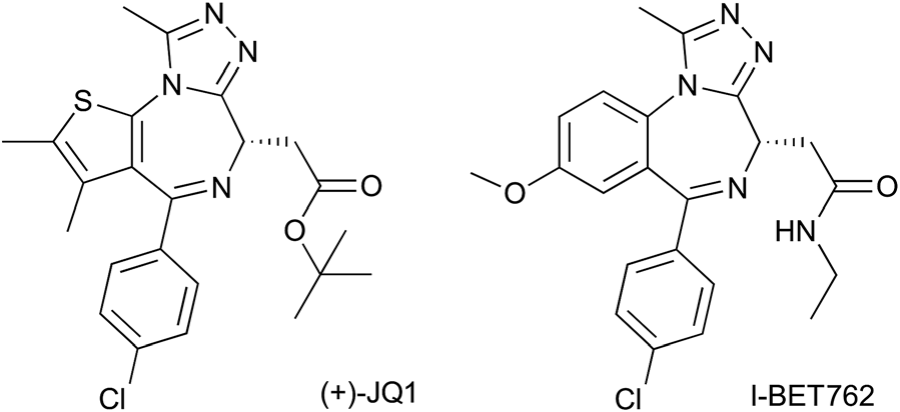
Benzodiazepine-based BET inhibitors.

In the last decade many high-quality small-molecule inhibitors of BET bromodomains have been developed (Chart 1), both for therapeutic and research purposes.^18^–^22^ The phenotypes generated by said inhibitors have been used to investigate the functions of BET proteins and their significance as therapeutic targets. This process has been limited by the pan-selective nature of the BET inhibitors, as they typically target all BET bromodomains with similar potency,^10^ hence specific proteins/bromodomains cannot be associated to specific phenotypes.^1^–^3^ Furthermore this pan-selectivity increases the possibility of side-effects limiting the usability of therapeutic BET inhibitors as all four BET proteins will be inhibited when only one may be disease-relevant.^23^ Additionally it is becoming increasingly apparent that the phenotypes generated by non-BET bromodomain inhibitors is in part driven by low-level BRD4 inhibition.^24^–^27^ Recently some advances have been made, as several inhibitors have been reported to be mildly selective for the ‘second’ bromodomains (BD2s)^11^,^28^,^29^ or the ‘first bromodomains (BD1s)^30^ of the BET proteins. One recently reported compound showed >10-fold selectivity for BRD4 BD1, through exploiting the differing dynamics of the ZA loop between different bromodomains.^31^ The BET bromodomains have also been successfully targeted for degradation by bifunctional PROTAC (proteolysis targeting chimera) compounds, based on existing BET inhibitor scaffolds^32^–^35^ and novel scaffolds.^36^ In our research we discovered a series of PROTACs that are BRD4-selective through the exploitation of novel protein–protein interactions between BRD4 and the VHL ubiquitin E3 ligase.^37^

**Fig. 1:**
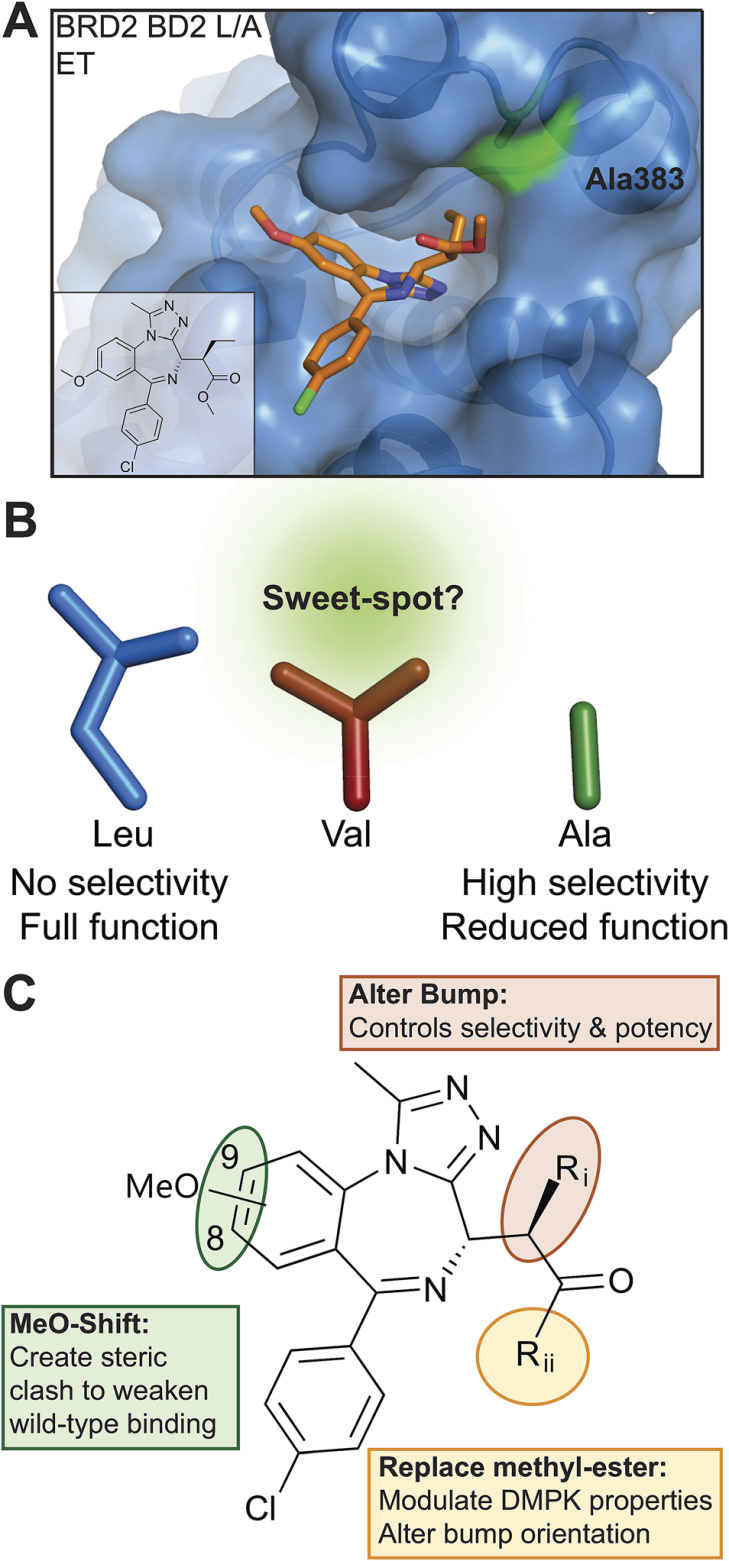
Bump-&-hole system optimization. (A) Co-crystal structure of ET bound to BRD2 BD2 L383A (4QEW). (B) Comparison of WT leucine and mutated residues. (C) Scaffold of bumped compounds, with modification sites highlighted.

For our bump-&-hole project^10^ we have previously identified a conserved leucine residue in the BET bromodomains binding site (L94 & L387 in BRD4) that can be substituted with an alanine, yielding relatively stable and functional bromodomain mutants. Compound ET – an I-BET762 (ref. 19)/JQ1 (ref. 18)-related benzodiazepine scaffold bearing an ethyl bump – targets the L/A mutation with high-affinity and ∼100-fold selectivity relative to wild-type (Fig. 1A). We have since worked to optimize, validate and implement this system. Although still capable of binding acetylated histone peptides the L/A mutants show a noticeable loss in binding affinity, and if not functional enough may compromise the viability of mutant cell-lines and animal models (Fig. 1B). Additionally, screening of diverse chemical modifications may deliver optimized inhibitors that are more selective and have improved physiochemical properties (Fig. 1C).

## Results

### An optimised leucine/valine mutation displays high structural & functional conservation

Here we explored the possibility of improving the bump-&-hole system through replacement of the previously described^10^ and potentially problematic L/A mutation (L94 and L387 in BRD4) (Fig. S1†). Through structural analysis of the bromodomain we hypothesized that a leucine/valine substitution (L/V) would be a more conservative change than the previous L/A substitution, resulting in a smaller ‘hole’ but still allowing enough space to accommodate a bumped ligand.

The mutant BET bromodomains were purified as single-bromodomain constructs, following site-directed mutagenesis, for *in vitro* characterization. This characterization focused on the ability of the bromodomains to bind and discriminate between acetylated histone peptides. We used differential scanning fluorimetry (DSF) to show that the BET bromodomain constructs were not destabilized by the L/V mutation (Table S1†).

**Fig. 2:**
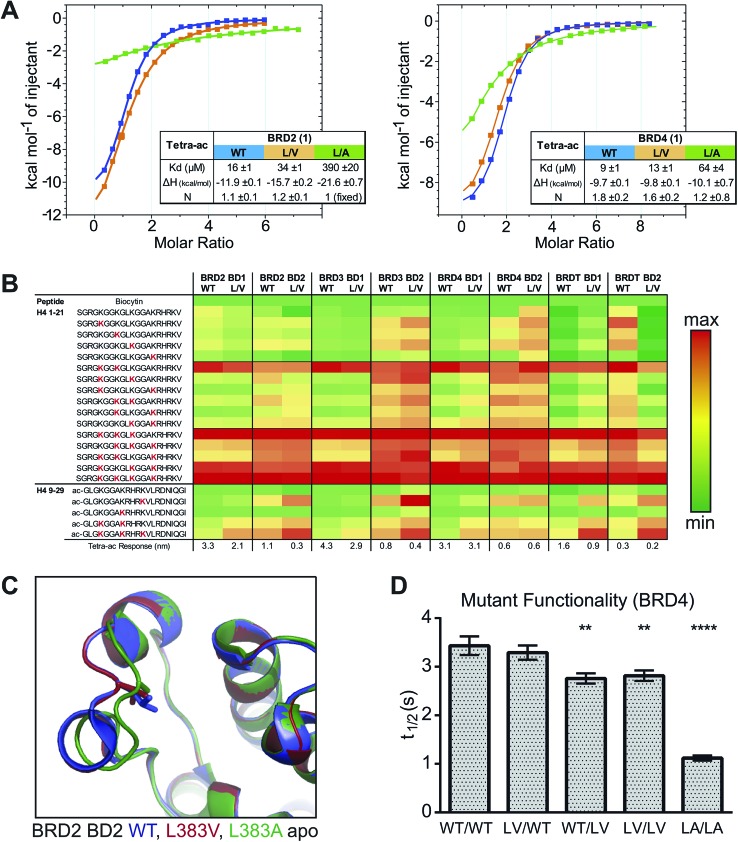
L/V mutant characterization. (A) ITC titrations of tetra-acetylated H4 peptide into BET bromodomains. (B) Binding profiles of WT and L/V bromodomains for acetylated H4 peptides, derived from BLI screen. Responses normalized to strongest response of each construct, and color-coded. Red ‘K’ in peptide sequence denotes lysine acetylation. (C) Alignment of BRD2 BD2 WT (2DVV), L/V and L/A (4QEU) apo structures, with leucine/valine/alanine highlighted. (D) Recovery times of GFP-labelled BRD4 constructs following 0.5 s laser bleach event, at 2 μM SAHA and 0.03% DMSO. Each bar is mean and SE of ∼50 U2OS cells tested over two separate experiments. Statistical significance determined with two-tailed *t* tests: ns *P* > 0.05; *P ≤ 0.05, **P ≤ 0.01, ***P ≤ 0.001, ****P < 0.0001.

We next assessed how the L/V mutation impacted the ability of the bromodomains to bind acetylated histone peptides. We used isothermal titration calorimetry (ITC) to measure the affinity and thermodynamic parameters for di-acetylated H4K(5,8)ac and tetra-acetylated H4K(5,8,12,16)ac substrate peptides^12^ binding to BET bromodomain constructs. The L/V mutation typically decreased the affinity of the peptide:bromodomain interaction by around two-fold (Table S2†), which is close to experimental errors and a significant improvement over the L/A mutation which showed up to 10-fold decreases in affinity.^10^ The supremacy of L/V over L/A was confirmed by titrations of H4K(5,8,12,16)ac against BRD2 BD1 and BRD4 BD1 L/A (Fig. 2A). Analysis of the thermodynamic parameters of binding (Δ*H*, *T*Δ*S*, Δ*G*) suggested that the L/V mutation does not substantially impact the binding mode of these peptides. In contrast, changes in these parameters relative to wild-type were much greater for the L/A mutation, consistent with a more detrimental effect (Fig. S2†).

The cellular function of the BET proteins is based not just on how strongly they bind histone peptides with specific epigenetic marks but also on what combinations of marks they recognize. To assess the impact of the L/V mutation on the binding profiles of the BET bromodomains we used bio-layer interferometry (BLI) to screen a library of acetylated histone peptides (Fig. 2B). Both WT and L/V bromodomains showed a marked preference for a cluster of poly-acetylated H4 peptides, especially H4K(5,8)ac, H4K(5,8,12)ac and H4K(5,8,12,16)ac. The WT and L/V binding profiles for all BD1s were virtually identical; and overall the L/V mutation had a visibly smaller effect on peptide recognition than the L/A mutant.^10^ No significant binding was observed for any non-H4 peptides (Fig. S3†).

To better understand how the L/V mutation affects histone binding, the X-ray crystal structure of the apo form of BRD2 BD2 L383V was solved and compared to the previously solved structures of WT and L383A BRD2 BD2 (ref. 10). Both the L/V and L/A mutants retain the overall structure of WT BRD2 BD2. The conformation of the ZA loop in the L/V mutant structure superposes very well with that of the WT (so-called “open” conformation), which differs from that observed in the L/A mutant, which is in a closed conformation (Fig. 2C). Notably however, the L/A mutant when ligand-bound had instead an open ZA loop (ref. 10, Fig. S4†). Re-orienting the ZA loop during binding might be contributing to the varying affinities observed between WT and mutant bromodomains for acetylated histone peptides. However, differences in crystallization space group and consequently crystal packing around the ZA loop amongst the various crystal structures might also contribute to the different conformations observed for the ZA loop.

To assess the functionality of the L/V mutants in a cellular environment we used a cellular fluorescence recovery after photobleaching (FRAP) assay to monitor the interaction between full-length BET proteins (GFP-tagged) and chromatin inside cells. Inhibition by ligands or deleterious mutations would reduce the proportion of GFP-tagged BET proteins bound to chromatin, increasing the rate of fluorescence recovery and decreasing the measured recovery time (*t*_1/2_).^10^,^38^ Mutant forms of BRD4 were compared in this assay and the L/V mutation was shown to have a much smaller impact on chromatin binding than the L/A mutation (Fig. 2D), in line with our *in vitro* data with histone peptides. Similar results were obtained for the L/V mutants of BRD2, BRD3 and BRDT (Fig. S5†). Together our biophysical and cellular data show that the L/V mutation is a major improvement over the L/A mutation, and has a minor effect on the structure of the BET bromodomains and their substrate binding properties.

### Chemical modifications for selective probing of the L/V bromodomains

In addition to optimizing the ‘biology’ of the bump-&-hole system (the mutation) we were interested in optimizing the ‘chemistry’ through the design of chemical probes superior in terms of binding selectivity, potency and DMPK properties.

First, to cover a range of bump sizes, we included primary alkyl methyl, ethyl and propyl as well as allyl ‘bump’ modifications. As the L/V mutation is more subtle than the previous L/A and is expected to generate a smaller ‘hole’, we decided not to include more sterically-demanding modifications.

Second, we explored the possibility of modifying the core scaffold with the aim to weaken binding to wild-type protein, potentially more so than against the mutant. Previous SAR studies on I-BET762 analogues described a number of chemical modifications to the benzodiazepine scaffold that resulted in weaker binding affinities to the WT BET bromodomains.^39^ One such modification, the shifting of the methoxy group from the 8′ to the 9′ position on the fused-phenyl ring, was deemed attractive as it reduced the BRD4 pIC_50_ by 0.5 log units and resulting analogues would retain very similar physicochemical properties to the parent compounds. We hypothesized that such a “Methoxy-shift” could enhance selectivity if the resulting steric clash was better accommodated by the mutated binding site compared to the WT protein.

Finally, we placed our attention to the flexible side chain on which the alkyl bump is located. Our ‘first generation’ compounds ME and ET both possess a methylester group at this position. Co-crystal structures showed the methylester side chain of ME and ET bound to the L/A mutant moved significantly compared to the bound conformation of the corresponding ethyl-amide group in I-BET762 bound to the WT protein.^10^ Such freedom to rotate could allow the bumped compound to accommodate itself in the WT binding pocket, leading to the residual affinity observed for ME and ET against WT. In contrast, ‘locking’ this side-chain in place would be expected to further weaken WT binding. We therefore decided to replace the methyl-ester side-group first with an amide, as in I-BET762, which would lock the side-group in place through a hydrogen bond from the amide NH to the ASN140 residue.^19^,^39^ In addition, we decided to include a *tert*-butyl ester group, as in JQ1, as this could form favourable hydrophobic interactions, and its bulky nature may limit the flexibility of the side-chain. The ethyl-amide and *tert*-butyl ester side-groups were also deemed as attractive ways to fine-tune the ADMET and physicochemical properties of our chemical probes.

Our L/V-selective ‘bumped’ compounds are derived from analogues of the known BET bromodomain probe, and clinical trial candidate, I-BET762. The synthesis and SAR of this 1,4-benzodiazepine scaffold is well described in the literature,^39^ allowing easy access to our scaffold I-BET-OMe (**1**) and its analogue incorporating the methoxy shift (9-IBET-OMe) (**2**). The key stage of the synthesis of all our bumped compounds was the addition of the sterically demanding alkyl ‘bump’ on the α-carbon. Potassium hexamethyldisilazane (KHMDS) was used to deprotonate in the α-position to the acetic acid methyl ester, to generate the desired enolate intermediate.^11^ These intermediates were then reacted with the appropriate alkyl iodides to form the desired α-alkylated derivatives (**3–9**) as diastereoisomeric mixtures.

From pervious work,^10^ the biologically active product presents a 2R*,3S* relative stereochemistry; however the diastereomeric mixture was at times inconveniently biased towards the inactive 2S*3S* diastereomer, which could be formed at an excess of up to 25-fold. When this occurred the diastereomeric mixtures were epimerized using sodium methoxide in anhydrous methanol under microwave irradiation.

Once the bumped group was installed, aqueous sodium hydroxide was used to hydrolyze the methyl-ester group and obtain the free carboxylic acids (**10–15**). Ethyl and di-ethyl amide groups (**16–22**) were introduced using standard peptide coupling conditions, using 1-[bis(dimethylamino)methylene]-1*H*-1,2,3-triazolo[4,5-*b*]pyridinium 3-oxid hexafluorophosphate (HATU) as the coupling reagent and *N*,*N*-diisopropylethylamine (DIPEA) as the amine base. *Tert*-butyl esters (**23–26**) were obtained from the carboxylic acids using *tert*-butyl trichloroacetimidate and boron-trifluoride as a catalyst.

**Scheme 1:**
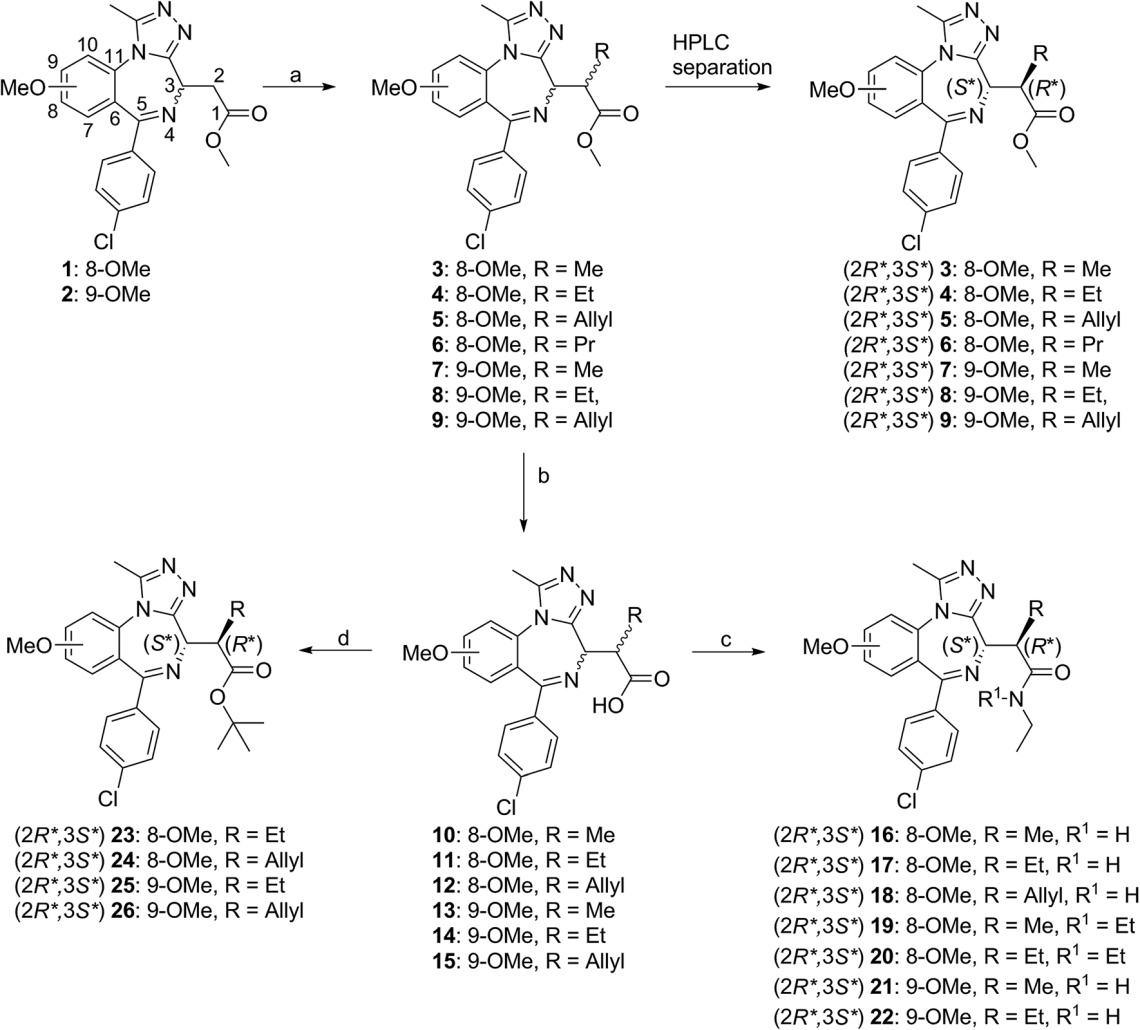
Bumped compound synthesis. (a) KHMDS, R-I, THF, –78–25 °C, 18 h, 24–56%; (b) NaOH, H_2_O, MeOH, 80 °C, 30 min, quant.; (c) HATU, DIPEA, NH2Et in THF, DCM, 25 °C, 2 h, 42–64%; (d) *tert*-butyl trichloro acetimidate, BF3*Et2O, DCM, 25 °C, 14–22%; for detailed synthetic procedures see ESI.†

All compounds were synthesized as diastereomeric mixtures. Reverse phase HPLC was then used to obtain pure samples of the 2R*,3S* diastereoisomer as a racemate, which was expected to contain the active compound, based on our previous work.^10^ Individual enantiomers were not separated at this stage of the project and unless explicitly stated otherwise, ligand concentrations and measured *K*_d_ and IC_50_ values refer to the concentration of the active enantiomer, *i.e.* half the concentration of the racemate (Scheme 1).

### Evaluation & SAR trends of bumped compounds

#### AlphaLISA & X-ray crystallography

Our compounds were first evaluated in a robust and efficient competitive AlphaLISA assay (Table 1, values for individual bromodomains are in Table S4†). The assay gave us broad SAR trends and allowed us to disqualify compounds lacking sufficient potency (L/V pIC_50_ ≥ 5.9) or selectivity (ΔpIC_50_ ≥ 1.3). This AlphaLISA assay measured the displacement from the bromodomain binding pocket of a biotinylated JQ1 probe^40^ (Bio-JQ1) (Fig. S6†), which we show to be a potent binder of both WT and L/V BET bromodomains (Fig. S7 & Table S3†). As SAR trends were identified from the data, a series of compounds showing potential as chemical probes were co-crystallized with L/V BRD2 BD2 and the resulting crystal structures used to rationalize experimental observations (X-ray data collection and refinement statistics are in the ESI†).

**Table 1:** SAR of bumped compounds

Compound	R_i_	R_ii_	MeO-position^*a*^	AlphaLISA pIC_50_ ± SD^*b*^			*C* log *P*^*c*^	Plasma *t*_1/2_ (min)	CL int (ml min^–1^ g^–1^)	*P*_e_ (nm s^–1^)	ITC p*K*_d_ ± SD^*d*^		
				WT	L/V	Δ					WT	L/V	Δ
(+)JQ1	H	O^*t*^Bu	—	6.6 ± 0.1	6.5 ± 0.1	–0.1 ± 0.1	4.8	>180	7.5				
(–)JQ1	H	O^*t*^Bu	—	4.8 ± 0.2	4.6 ± 0.1	–0.2 ± 0.2	4.8						
I-BET762	H	NHEt	8	6.5 ± 0.2	6.5 ± 0.3	0.0 ± 0.3	2.8	>180	1.3	25			
**1**	H	OMe	8	6.6 ± 0.2	6.6 ± 0.3	0.0 ± 0.2	3.3	54	<0.5	149	6.8	6.5	–0.3
**2**	H	OMe	9	5.8 ± 0.2	5.9 ± 0.2	0.1 ± 0.1	3.3	67	1.7				
**3**	Methyl	OMe	8	6.2 ± 0.3	7.4 ± 0.1	1.2 ± 0.2	3.5	>180	0.7	185			
**4**	Ethyl	OMe	8	5.0 ± 0.1	6.8 ± 0.2	1.7 ± 0.2	3.9	>180	1.5	153	5.1 ± 0.5	6.9 ± 0.3	1.8 ± 0.4
**5**	Allyl	OMe	8	5.1 ± 0.1	6.6 ± 0.3	1.5 ± 0.3	3.9	>180	6.7	127	5.1 ± 0.4	6.6 ± 0.2	1.5 ± 0.5
**6**	Propyl	OMe	8	4.5 ± 0.2	5.0 ± 0.1	0.6 ± 0.2	4.3	>180	5.2				
**7**	Methyl	OMe	9	5.4 ± 0.2	6.8 ± 0.2	1.4 ± 0.2	3.5	>180	<0.5	158	4.5 ± 0.2	6.6 ± 0.3	2.0 ± 0.4
**8**	Ethyl	OMe	9	5.5 ± 0.2	6.8 ± 0.2	1.4 ± 0.2	3.9	>180	4.1	155	<4.2	6.4 ± 0.2	>2.2 ± 0.2
**9**	Allyl	OMe	9	4.4 ± 0.1	5.7 ± 0.2	1.3 ± 0.2	3.9	>180	9.4	136			
**16**	Methyl	NHEt	8	5.3 ± 0.2	6.7 ± 0.2	1.4 ± 0.2	3.1	>180	1.2	26	5.6 ± 0.3	6.9 ± 0.2	1.3 ± 0.3
**17**	Ethyl	NHEt	8	4.4 ± 0.1	5.9 ± 0.3	1.5 ± 0.2	3.5	>180	1.4	45	<4.2	6.0 ± 0.2	>1.8 ± 0.2
**18**	Allyl	NHEt	8	4.3 ± 0.1	5.9 ± 0.2	1.5 ± 0.1	3.5	>180	2.6	52			
**19**	Methyl	N[Et]_2_	8	4.9 ± 0.2	6.1 ± 0.2	1.2 ± 0.2	3.8	>180	>50				
**20**	Ethyl	N[Et]_2_	8	4.6 ± 0.1	5.8 ± 0.2	1.2 ± 0.2	4.1	>180	>50				
**21**	Methyl	NHEt	9	4.7 ± 0.3	6.0 ± 0.2	1.3 ± 0.2	3.1	>180	1.5	40	4.5 ± 0.4	6.3 ± 0.2	1.8 ± 0.5
**22**	Ethyl	NHEt	9	<4.0	5.2 ± 0.2	>1.2	3.5	>180	2.1	59			
**23**	Ethyl	O^*t*^Bu	8	5.3 ± 0.1	6.0 ± 0.2	0.7 ± 0.1	4.8	>180	30.7				
**24**	Allyl	O^*t*^Bu	8	5.3 ± 0.1	6.2 ± 0.2	0.9 ± 0.2	4.9	>180	39.1				
**25**	Ethyl	O^*t*^Bu	9	4.6 ± 0.1	5.0 ± 0.2	0.4 ± 0.2	4.8	>180	31.0				
**26**	Allyl	O^*t*^Bu	9	5.2 ± 0.2	5.8 ± 0.2	0.5 ± 0.1	4.9	>180	39.4				

^*a*^JQ1 compounds possess a thiophene ring with no methoxy group.

^*b*^Values are mean and standard deviation of all somatic bromodomains – BD1 and BD2 of BRD2, BRD3 and BRD4.

^*c*^ *C* log *P* calculated in StarDrop.

^*d*^Values are mean and standard deviation of all somatic bromodomains except for **1** which was titrated only against BRD4 BD1. Color-coded version of this table is available as Fig. S8.

Our scaffold compound **1** was as potent as I-BET762 & (+)JQ1 against WT bromodomains, which was unsurprising given their structural similarity. Meanwhile, ITC titrations with **1** provided *K*_d_ values of 150 nM and 290 nM for BRD4 BD1 WT and L/V, respectively. Due to the assay's high sensitivity we were able to detect very weak displacement by the inactive (–)JQ1 isomer at high concentrations. Several bumped compounds (*e.g.***17–22**) showed similarly low potency, which we interpret as them being unlikely to bind WT BET proteins at commonly used concentrations.

Many of our bumped compounds showed promise as potent and selective probes of L/V BET bromodomains. Methyl, ethyl and allyl bumps (**3–5**), the 9′ methoxy group (**7–9**, **21**) and the ethyl-amide side-group (**16–18**, **21**) typically met our criteria. Overall this dataset shows the efficacy of the bump-&-hole approach, with only the propyl bump (**6**) and *tert*-butyl ester compounds (**23–26**) showing less than 10-fold selectivity. Moreover, a wide range of selectivity and potency was observed across the series, showing that the activity of bumped compounds can be fine-tuned with the right modifications.

**Fig. 3:**
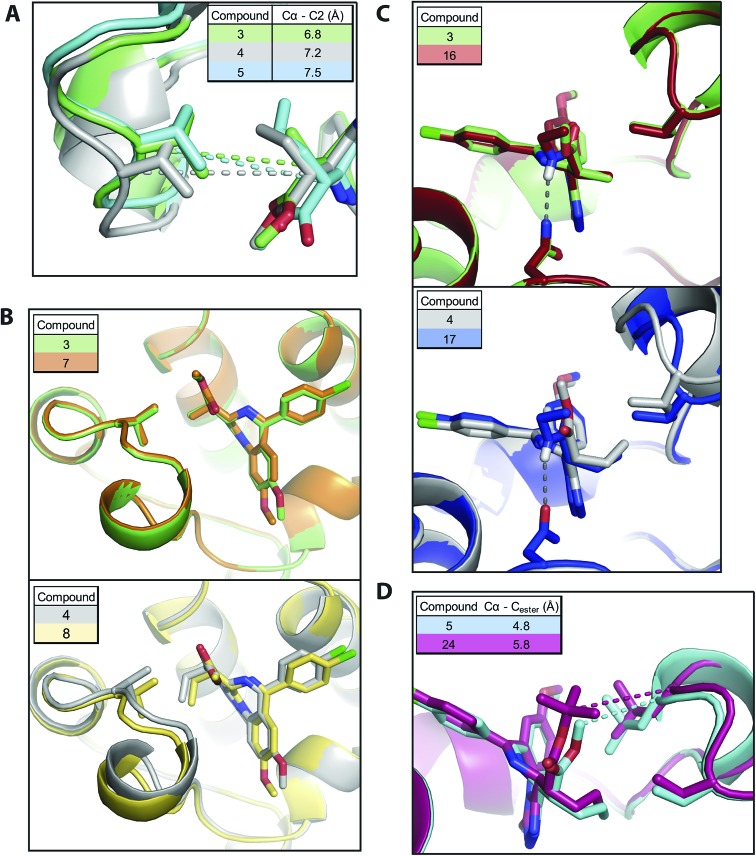
Compound modifications & BRD:ligand co-crystal structures. (A) comparison of alkyl bumps; (B) effect of methoxy shift; (C) effect of ethyl-amide group; (D) effect of *tert*-butyl ester. Structural alignments of different bumped-compounds co-crystallized with BRD2 BD2 L/V. Dark grey dashes represent hydrogen bonds. Other dashes show inter-atom distances of interest. Cα = alpha carbon of valine or other residue. C2 = 2′ carbon of compound. C_ester_ = carbon following ester bond.

As expected the addition of alkyl bumps weakened binding for WT bromodomains and is necessary for L/V selectivity. The smaller bumps increase potency for L/V bromodomains, as the bump and hole now form a new hydrophobic interaction. The effect of the bump on potency mostly results from its size, as shown by the highly potent **3** binding much more intimately within the L/V hole (Fig. 3A). With larger bumps, however, the rotational flexibility of the bump becomes important, with the semi-rigid **5** bump acting more like the equally flexible **4** bump than the **6** bump of similar length.

Shifting the methoxy group to the 9′ position (**7–9**) did not cause large changes in the L/V potency of bumped compounds, despite its pronounced effect on the scaffold (**2**). The methoxy shift only clearly enhanced selectivity when paired with the methyl bump (**7**), where it also does not alter the compound binding orientation (Fig. 3B).

The replacement of the methyl-ester with an amide group results in a pronounced reduction on WT binding affinities. Co-crystal structures (Fig. 3C) confirm the formation of the expected hydrogen bond to Asn140. As this hydrogen bond locks the bump in an orientation facing the WT leucine residue it exacerbates the steric clash.

The ethyl-amide side-group and its hydrogen bond does not reduce potency in non-bumped scaffolds, as can be seen with I-BET762 and **1**. Compounds featuring the di-ethyl amide side-group (**19**, **20**) show greater WT potency and lower selectivity than ethyl-amide compounds.

An alternative methyl-ester replacement was the *tert*-butyl ester group (**23–26**), present in the BET inhibitor JQ1. This group had a very deleterious effect on selectivity, as it both increased WT potency and reduced L/V potency. Co-crystal structures (Fig. 3D) of BRD2 BD2 L/V bound to **5** and **24** show that the *tert*-butyl group clashes with Leu381, reducing L/V potency. As this clash pushes the ligand away from the ZA loop it may relieve the bump/leucine steric clash and increase WT potency.

### DMPK triage

The AlphaLISA screen suggested several promising compounds showing high potency for L/V bromodomains and selectivity against WT. A secondary triage of the compounds, investigating their DMPK qualities, was conducted to eliminate potential candidates with poor PK properties that would later undermine their utility as chemical probes in cells and *in vivo*. To proceed beyond this triage compounds were required to show no breakdown in plasma, microsomal clearance rates similar or lower than existing BET probes and high apparent permeability (>10 nm s^–1^).

Pleasingly, all bumped compounds were very stable in plasma (half-lives over 3 hours), while the scaffold compounds (**1**, **2**) had lower stability, likely due to esterase activity (Table 1). This is consistent with the theory that the presence of an alkyl bump on the α-carbon is important to increase the ester group stability, and suggested that replacement of the methyl-ester side-group was not necessary. Unlike in plasma, **1** and **2** were found to be very stable in liver microsomes, showing low intrinsic clearance, which is an indicator of low CYP450 metabolism.^41^ Compounds bearing the methoxy shift and ethyl-amide side-group showed very little clearance, while the hydrophobic *tert*-butyl ester (**23–26**) and di-ethyl-amide side groups (**19**, **20**) produced unacceptably high (>30 ml min^–1^ g^–1^) clearance rates (Table 1).

Compounds that had passed previous selection criteria, and others still deemed of interest, were then tested in the *in vitro* PAMPA assay (Table 1), an artificial model of cell permeability. All tested compounds with a methyl-ester side-group (**1**, **3–9**) show extremely high permeability, with *P*_e_ values between 127 and 185 nm s^–1^, whereas those compounds with the ethyl-amide side-group (**16–18**, **21**, **22**) show 25 to 59 nm s^–1^. The PAMPA and microsomal clearance data confirms our hypothesis that compound DMPK properties could be tuned through side-group modifications while altering the position of the methoxy group has little impact on DMPK properties.

### Full ITC profiles

To finally determine the best bumped compound(s) a small number of compounds were titrated against all somatic BET bromodomains, WT and L/V, in ITC (Table 1). The following compounds met the selection criteria for both the AlphaLISA assay and DMPK triage and hence underwent ITC profiling: **4**, **5**, **7**, **8**, **16**, **17** and **21**. Results for specific bromodomains can be found in Table S5.†

9-ME (**7**) and 9-ET (**8**) were clearly the most promising, with selectivity values >100-fold, and their ITC profiles were replicated until reliable values of potency and selectivity could be generated. **7** is more potent against both L/V and WT bromodomains, while **8** shows greater overall selectivity (Table S6†). **7** was used for the majority of our cellular experiments, as it was the most potent compound to show >100-fold selectivity and has slightly better DMPK properties.

The remaining compounds were not as promising, but are likely still usable as allele-selective inhibitors, and could be preferable in certain contexts. **4** and 1**6** show very high potency, while **17** was the only compound to show no detectable binding to any WT BET bromodomain.

### Validation of bump-&-hole system

**Fig. 4:**
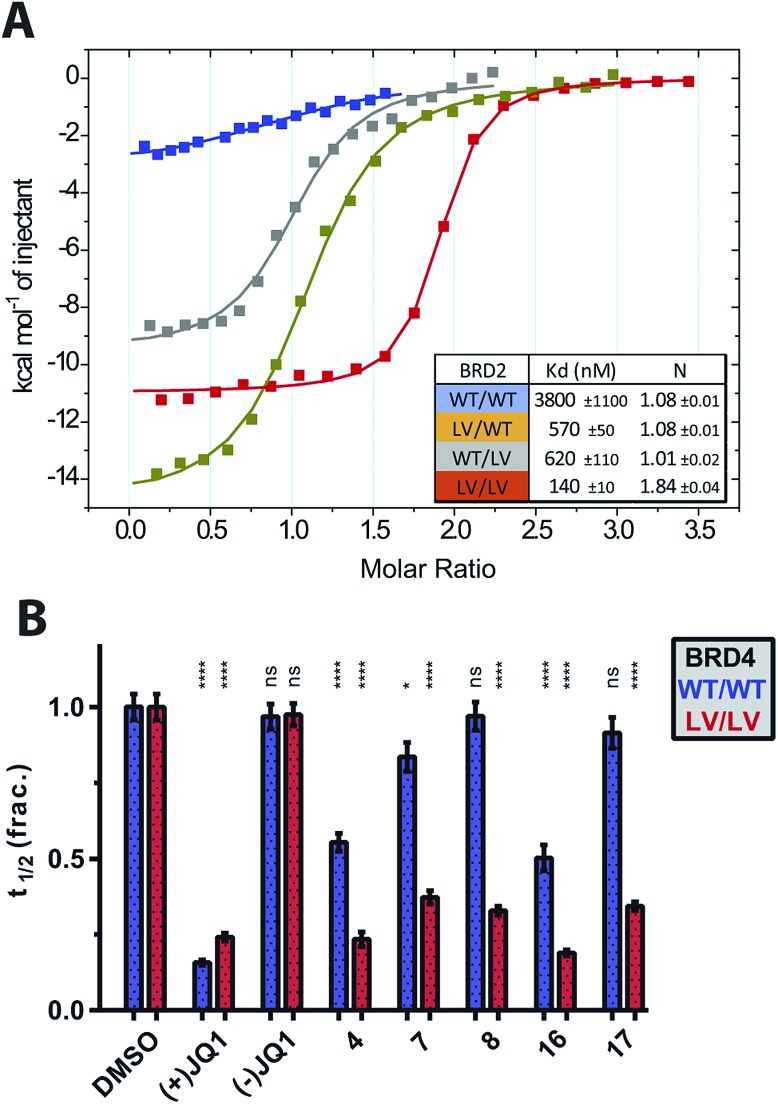
Bumped compounds can inhibit single bromodomains and are effective in cells. (A) ITC titrations of **4** into BRD2 tandem constructs containing both bromodomains. (B) Effects of range of compounds on fluorescence recovery of GFP-labelled BRD4 constructs in U2OS cells, following 0.5 s laser bleach event, at 1 μM compound, 2 μM SAHA and 0.03% DMSO. Statistical significance determined with two-tailed *t* tests: ns *P* > 0.05; *P ≤ 0.05, **P ≤ 0.01, ***P ≤ 0.001, ****P < 0.0001.

The ability of the bump-&-hole system to target a single bromodomain, within one BET protein, was shown through ITC titrations of **4** against BRD2 constructs containing both bromodomains. Through measurements of the ligand:protein stoichiometry it was confirmed that **4** bound with a 2 : 1 stoichiometry when both bromodomains were mutated, and 1 : 1 stoichiometry to the single-bromodomain mutants. Furthermore negligible WT binding was observed (Fig. 4A).

To test the potency and selectivity of our bumped compounds in cells we used a fluorescence recovery after photobleaching (FRAP) assay, using U2OS cells over-expressing GFP-labelled full-length BET proteins.^10^,^38^ While the BET proteins are active and bound to chromatin fluorescence recovery times can be several seconds long. However, when the proteins are displaced by compounds and freely diffusing in the nucleus the fluorescence recovers much faster. The HDAC inhibitor suberoylanilide hydroxamic acid (SAHA) was used to increase the assay window by increasing chromatin acetylation and thus reducing the levels of ‘free’ GFP-BET protein (Fig. S9†). Several compounds were tested against WT and L/V BRD4 (Fig. 4B) and were shown to enter cells and displace full-length BET proteins from chromatin in an L/V-selective manner. **7** was confirmed to be potent and selective in cells, and this compound was hence used further.

### Enantiomer separation

At this point in the project, with the best compounds identified, it was decided to separate the two enantiomers from our racemic mixtures. A method for said separation was developed by Reach Separations Ltd. Racemic mixtures were dissolved to 20 mg ml^–1^ in ethanol and purified by HPLC, using a Lux A1 column (21.2 mm × 250 mm, 5 μm) at ambient temperature and a flow rate of 21 ml min^–1^. Samples were injected at a volume of 1 ml with 4 : 6 HEPT : EtOH (0.1% v/v NH_3_). A 42 mg sample of **7** at 96% purity could be separated into two clear peaks. The first peak to elute yielded 11 mg of compound (chemical purity of 94% and enantiomeric excess of 98), while the second peak yielded 9 mg, at 98% purity and enantiomeric excess of 97.

**Fig. 5:**
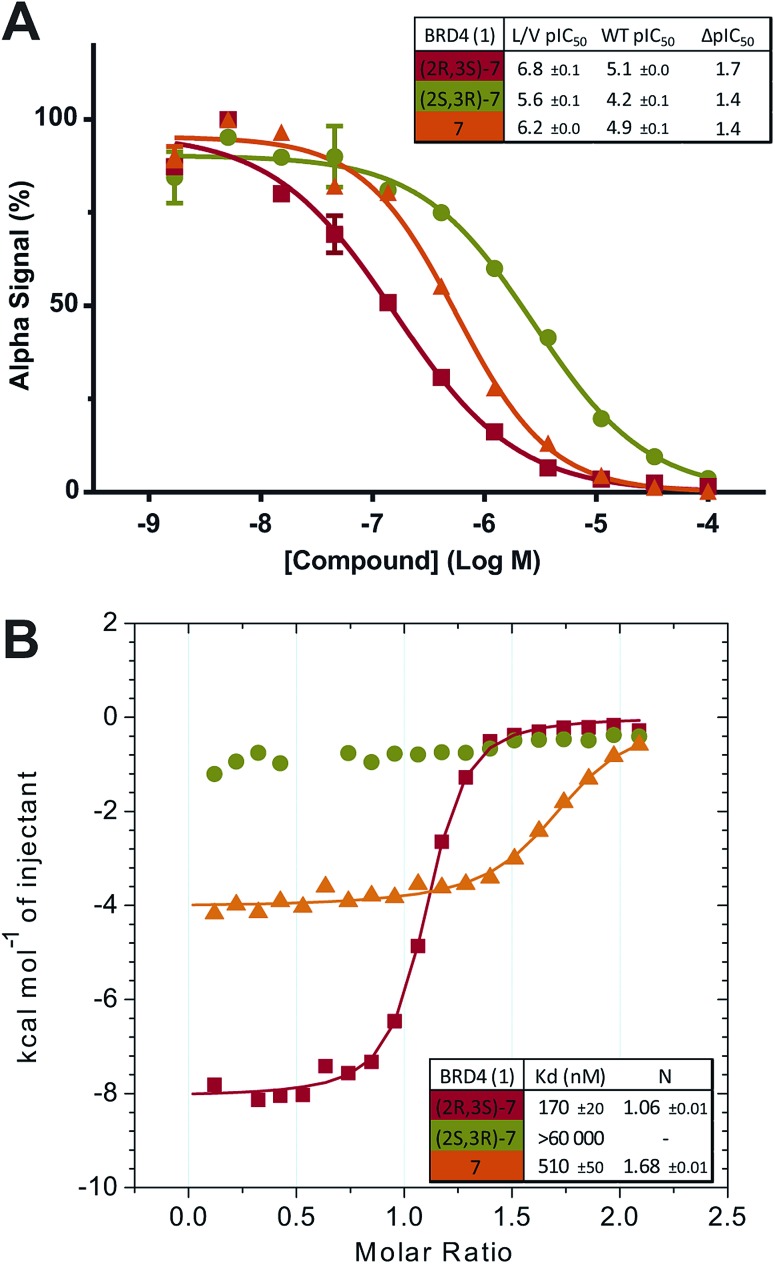
Enantiomer Characterization. Purified **7** enantiomers, and racemic mixture thereof, titrated against BRD4 BD1 L/V in competitive AlphaLISA assay (A) and ITC (B).

Separated enantiomers were next titrated against BRD4 BD1 in the AlphaLISA assay (Table S7†). The compound in the first elution peak was more potent than the racemic mixture, and even more so than the compound eluted in the second peak (Fig. 5A). We can assign the active enantiomer as **(2*R*,3*S*)-7**, and consequently the less active enantiomer as **(2*S*,3*R*)-7**. This assignment is based on analyses of the co-crystal structures. In all structures obtained, electron density around the chiral centres in question was well resolved (Fig. S10†). All ligands could be fitted with high quality (ligand real space correlation coefficient ≥0.87 and real space *R*-value ≤0.18). Based on these analyses, we therefore conclude that the (2*R*,3*S*) enantiomer is the more active compound.

ITC titrations (Table S8†) showed the (2*R*,3*S*) enantiomers of **7** and **8** to bind BRD4 BD1 L/V with high affinity and a 1 : 1 stoichiometry, while the racemic mixtures showed ∼2-fold decreases in affinity and enthalpy and *N* values close to 2 (Fig. 5B), consistent with only half the mixture binding the bromodomain. Finally no detectable binding was observed for the (2*S*,3*R*) enantiomers.

**Fig. 6:**
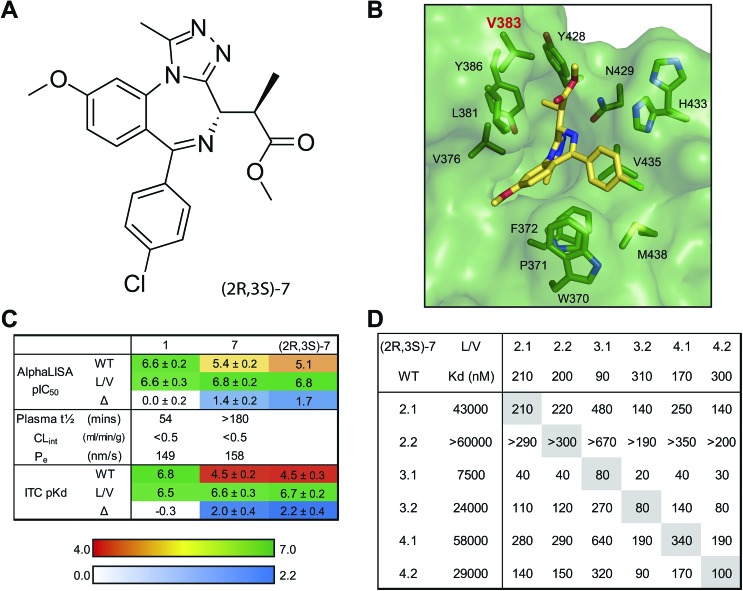
**(2*R*,3*S*)-7**: a potent and highly-selective bumped BET inhibitor. (A) Chemical structure of **(2*R*,3*S*)-7**. (B) Co-crystal structure of **(2*R*,3*S*)-7** bound to BRD2 BD2 L/V, with key residues highlighted. (C) SAR of scaffold (**1**), **7** and **(2*R*,3*S*)-7**. **(2*R*,3*S*)-7** AlphaLISA data and **1** ITC data from BRD4 BD1 only, other data is mean ± SD of titrations against all somatic BET bromodomains. (D) Selectivity plot of **(2*R*,3*S*)-7**.

Although the inactivity of the second enantiomer means racemic mixtures can still be reliably used in experiments we chose to exclusively use the active enantiomer going forward. ITC titrations (Fig. S11†) showed **(2*R*,3*S*)-7** to be in general more potent and mutant-selective than the racemic mixture. The use of the purified active enantiomer overall boosts potency and selectivity and reducing the total compound concentration by half will provide other benefits (easier dosing, reduced compound metabolism). We therefore present 9-ME-1 (Fig. 6) as our preferred bumped inhibitor, showing high potency, selectivity for L/V BET bromodomains and a strong DMPK profile. The (2*S*,3*R*) enantiomer – 9-ME-2 – can also be used as an inactive control.

### WT cytotoxicity

**Fig. 7:**
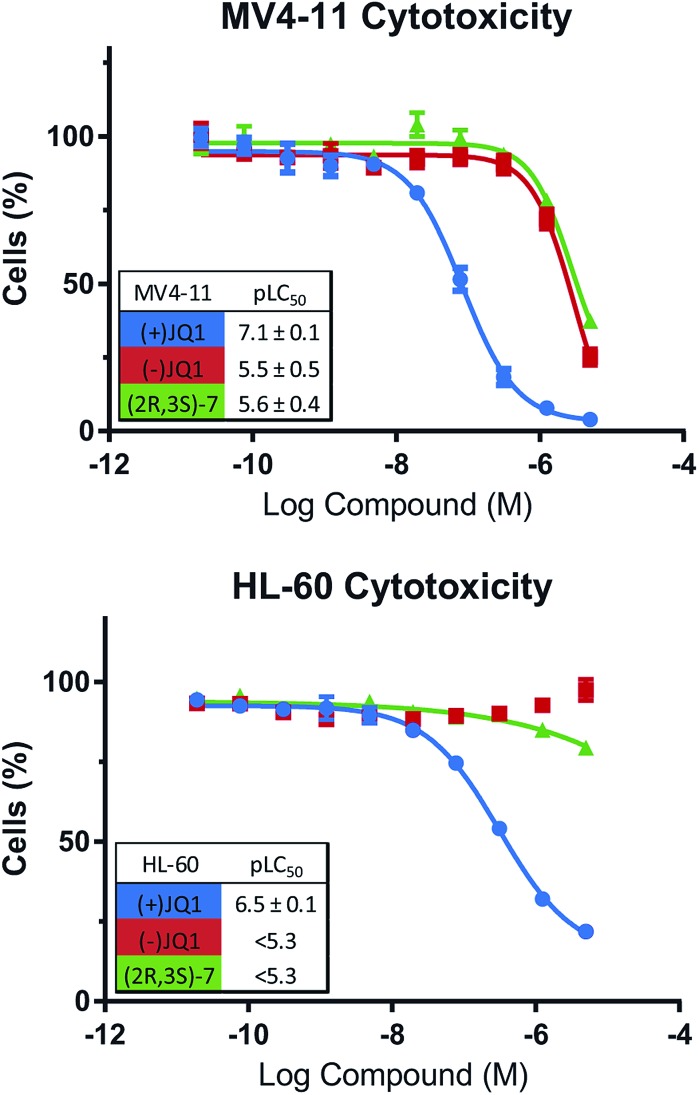
**(2*R*,3*S*)-7** does not perturb WT BET-dependent cells. Effects of compounds on viability of BET-dependent cell-lines MV4-11 & HL-60.

To confirm that our bumped compounds will not inhibit WT BET proteins at commonly used concentrations, nor alter the phenotypes of BET-sensitive cells we assayed the activity of our compounds on the viability of BET-dependent AML cell-lines MV4-11 and HL-60 (ref. 14) (Fig. 7). Pleasingly, **(2*R*,3*S*)-7** affected these cell-lines at a similar level to the ‘inactive’ (–)JQ1 control, and showed no cytotoxicity below 1 μM. This data supports the use of **(2*R*,3*S*)-7** for allele-selective BET inhibition at commonly used concentrations (100 nM–1 μM).

### Off-target screening

We have used several techniques to show that our bumped compounds are strongly selective for L/V BET bromodomain mutants. To show selectivity against non-BET WT bromodomains we employed the BROMOscan screen (DiscoveRX), testing 32 human bromodomains (Fig. S12†). Using BROMOscan technology we first tested our scaffold (**1**) as a positive control. Against BRD4 BD1 WT this gave a *K*_d_ value of 15 nM and showed >90% inhibition above 100 nM. **(2*R*,3*S*)-7** was screened at 1 μM and was found to bind non-BET bromodomains to a lesser degree than BET bromodomains. Some overlap was observed for SMARCA2, SMARCA4 and WDR9 BD2, an identified off-target of JQ1 (ref. 18).

To check for any unexpected off-target activity outside of the BET bromodomain subfamily we employed two high-quality screens. A screen of 50 representative human kinases showed no more than 20% inhibition at 1 μM **(2*R*,3*S*)-7** (Table S9†). A test of 55 receptors, transporters and ion channels (Table S10†) showed 20% or less inhibition at 1 μM **(2*R*,3*S*)-7**, with the exception of the melatonin receptor MT1 which showed 77% inhibition. Undergoing the same screen, JQ1 also had previously shown off-target inhibition of MT1, in addition to the adenosine A3 receptor and the neurokinin NK2 receptor.^18^

### Application to a biological question

Some aspects of basic BET protein function are still unclear, such as the roles and relative importance of the first and second bromodomains within each protein. The BD1 of BRD4 has long been thought^12^ to play a greater role in chromatin binding than its BD2. Experiments with ChIP-seq and acetylated nucleosome libraries^42^ show that BRD4 BD1 is alone sufficient for chromatin binding, although it is enhanced by the BD2, while we have previously shown the BD1 of BRD4 is required for chromatin binding.^10^ Recently it was shown that only the BD1 of BRDT is capable of binding acetylated nucleosomes, and the role of the BD2 may instead be to recruit acetylated non-histone proteins.^43^ Currently little is known about the mechanisms of BRD2/BRD3 chromatin binding and the relative importance of their BD1s and BD2s.

**Fig. 8:**
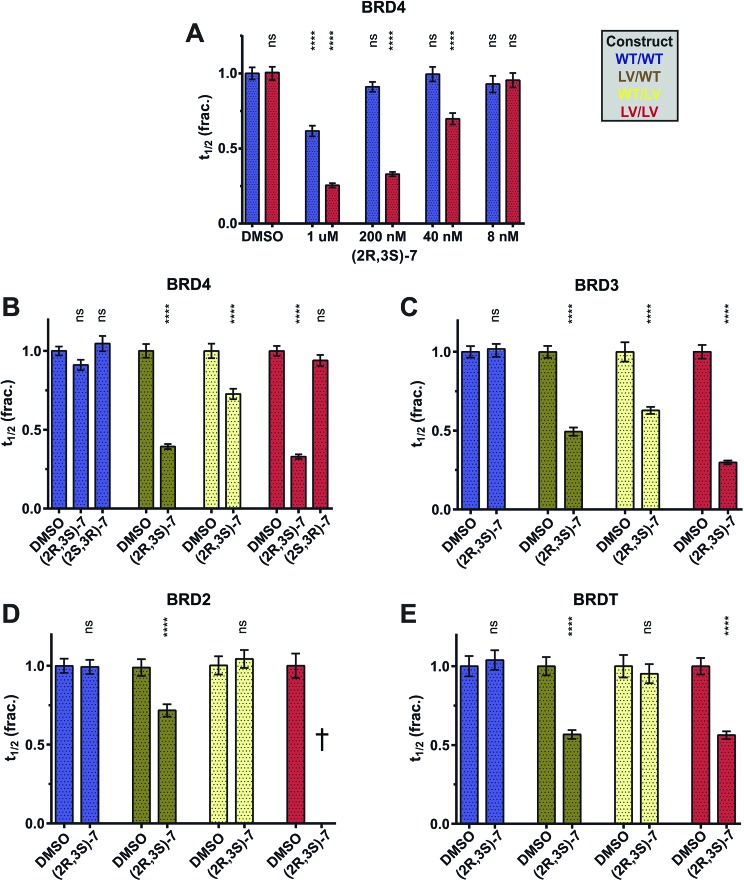
Application of the bump-&-hole system to a biological question. (A–E) Effects of **7** enantiomers on the fluorescence recovery of GFP-labelled full-length BET constructs in U2OS cells, following 0.5 s laser bleach event, at 2 μM SAHA and 0.03% DMSO. Compound concentration is 200 nM unless stated otherwise. Unpaired *t* tests compare the effects of each compound to said construct's DMSO control. Results are mean and SEM of ∼50 cells tested over 2 separate experiments. †–*t*_1/2_ could not be determined due to inhibition-induced aggregation. Statistical significance determined with two-tailed *t* tests: ns *P* > 0.05; *P ≤ 0.05, **P ≤ 0.01, ***P ≤ 0.001, ****P < 0.0001.

Our development of the highly selective **(2*R*,3*S*)-7** probe and improved L/V mutation motivated the application of the bump-&-hole system to answer these biological questions. By testing **(2*R*,3*S*)-7** against GFP-BET constructs in our FRAP assay and comparing its effects on BD1 *vs.* BD2 mutants we can observe their relative importance to chromatin binding. A dose of 200 nM was chosen as it showed no statistically significant blockade of WT BRD4 alongside almost complete displacement of L/V BRD4 (Fig. 8A). As the L/V mutation has a minor impact on uninhibited recovery times (Fig. S5†) the effects of inhibition on different constructs can be reliably compared.

Our data shows that, for all BET proteins, inhibition of BD1 has a greater effect than that of BD2 (Fig. 8B–E). Interestingly, the degree to which BD1 is dominant varies between the BET proteins. BD2 inhibition had no impact on BRDT (Fig. 8E), and **(2*R*,3*S*)-7** had the same effect on both the LV/WT and LV/LV constructs. These data are consistent with a model in which BRDT BD2 is not involved in chromatin binding, a conclusion recently drawn by Miller and colleagues.^43^ BRD4 (Fig. 8B) did see a small change in *t*_1/2_ in response to BD2 inhibition, suggesting (alongside previous experiments from us^10^ and others^42^) that it has a minor role in chromatin binding. Finally BRD3 (Fig. 8C) shows the greatest impact of BD2 inhibition, and mutation of both bromodomains is necessary for full displacement from chromatin, suggesting a much more balanced mechanism of bromodomain:histone binding. We cannot be certain of the role of BRD2 BD2 (Fig. 8D) as the WT/LV construct shows no change in *t*_1/2_ but BD1 inhibition does not match that of the double mutant. Inhibition of the double mutant could not be quantified, as the GFP-BRD2 construct aggregated in the nucleus. This phenomenon was shown to be connected to strong inhibition and to occur with the WT construct and hence not be due to any destabilizing effect of the L/V mutation (Fig. S13†). Inhibition-triggered aggregation of bromodomain constructs has previously been observed.^44^

**Fig. 9:**
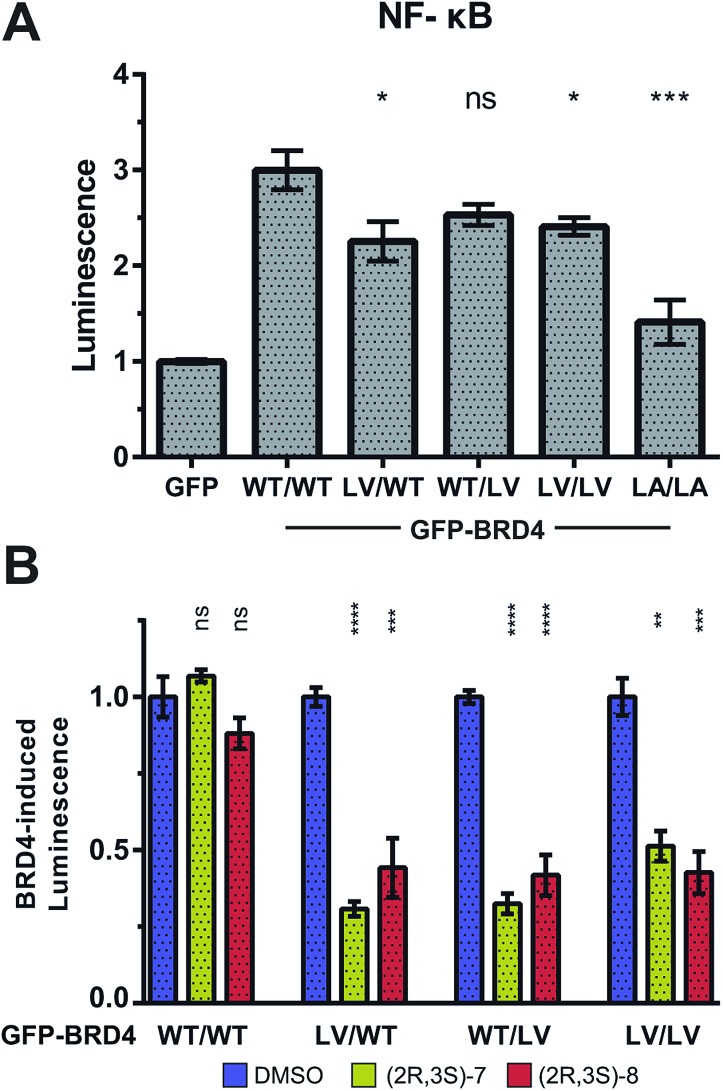
BRD4 and NF-κB-target gene expression. Luminescence of HEK cells transfected with GFP-labelled BRD4 constructs and a NF-κB-RE/*luc*2*P* reporter plasmid. (A) Luminescence normalised to GFP control. Statistical significance indicators relate to WT BRD4. (B) Basal luciferase expression (with GFP plasmid) subtracted as background. Signal normalised to each construct's DMSO control. Results are mean and standard error of six technical replicates spread over two independent experiments. Statistical significance determined with two-tailed *t* tests: ns *P* > 0.05; *P ≤ 0.05, **P ≤ 0.01, ***P ≤ 0.001, ****P < 0.0001.

To provide additional functional insights into the individual roles of BD1 *vs.* BD2 beyond chromatin binding, a luciferase assay was developed to monitor the expression of NF-κB target genes, inspired by work of Zou *et al.*^45^ Expression of our NF-κB-controlled luciferase was significantly increased by overexpression of GFP-BRD4 (on top of endogenous BET protein). This luciferase induction was maintained with L/V mutations (but not L/A), consistent again with more WT-like functionality of L/V compared to L/A (Fig. 9A).

Treatment of **(2*R*,3*S*)-7** and **(2*R*,3*S*)-8** against each GFP-BRD4 L/V construct showed that inhibiting either individual BD of BRD4 strongly impacted on NF-κB signalling, matching that of inhibiting both BDs (Fig. 9B). Together, the FRAP and luciferase data are consistent with the BD2 having little role in chromatin binding, but still being vital for initiating transcription and regulating gene expression, at least in the case of BRD4 and NF-κB.

## Discussion

Through a variety of techniques we have optimized both the biological and chemical aspects of our BET-bromodomain targeting bump-&-hole system. From our library of bumped compounds we have highlighted **(2*R*,3*S*)-7** which shows high potency and strong selectivity for our L/V mutation, which we have shown to be more structurally and functionally conservative than the previous L/A mutation.

We applied our optimized system to address a biological question – the relative importance of the BD1s and BD2s of BET proteins to chromatin binding.^10^,^42^,^43^ By dosing a variety of GFP-BET constructs in our FRAP assay we showed that, for all BET proteins, chromatin binding is primarily influenced by BD1s. Furthermore we could show that the degree to which the BD1 is dominant varies between BET proteins and that BRD3 is sensitive to BD2 inhibition. Interestingly our GFP-BRDT construct generates significantly slower recovery times (*t*_1/2_ ∼ 1.5 s) when uninhibited. This could indicate BRDT being a weaker binder of acetylated chromatin, or a result of U2OS not presenting BRDT's preferred epigenetic marks. These observations show that when using this bump-&-hole system it may be necessary to mutate both bromodomains to fully displace a given BET protein. If the BD2s of certain BET proteins do not primarily function through the binding of chromatin then an alternate function could be the recognition and recruitment of acetylated, non-histone proteins. Several such interactions have been reported,^46^–^49^ such as an interaction between BRD4 BD2 and di-acetylated Twist.^50^ The unusual BD1/BD2 balance displayed by BRD3 suggests its chromatin and non-chromatin mediated biological functions may also be atypical.

By combining our bump-&-hole system and a NF-κB luciferase assay we were able to assess the importance of the BD1 and BD2 of BRD4 to initiating transcription and regulating gene expression (for NF-κB target genes). Our data revealed that BRD4 BD2 is still vital for transcription initiation, despite having a minor role in chromatin binding. This suggests that recruitment of non-histone proteins is also essential. This is consistent with the model put forth by Shi *et al.*^50^ wherein BRD4 BD2 binds Twist, a transcriptional activator, giving the BRD4 – P-TEFb – RNA Pol II complex specificity for WNT5A. One can imagine that there are other non-histone proteins (NF-κB in our example^45^) recognised by BRD4 BD2, and other BET BDs not involved in chromatin binding, that can direct BET proteins to up-regulate other specific sets of genes. In the future, a more widespread and systematic investigation into such non-histone BET binding partners could reveal much about how they function on a molecular level, and present new opportunities for drug or chemical probe development.

Recently the results of BET-inhibitor clinical trials have highlighted the risks of on-target toxicity, especially thrombocytopenia.^23^,^51^–^55^ The BD2-selective RVX-208 (ref. 28) is the only BET-inhibitor to progress to phase 3, hinting at reduced toxicity.^56^ This low toxicity could be a result of the reduced role of BD2s in chromatin binding through three potential routes. As the BD2s are less important to chromatin binding RVX-208 may generate only partial BET inhibition and a greater therapeutic window. As the role of the BD2 differs between BET proteins this could generate some inter-protein selectivity for BRD3 over BRD4, reducing any BRD4-mediated toxicity. Finally, RVX-208 may not function through chromatin displacement but by blocking interactions between BD2s and non-histone proteins (such as Twist) and hence act through a more precise, less toxic, mechanism.

Through a combination of selectivity screens we show **(2*R*,3*S*)-7** to have almost no off-target inhibition. The only significant off-target is the melatonin receptor MT1. MT1 has been shown to have a variety of functions,^57^ primarily taking place in the CNS and regarding the circadian rhythm, which should not complicate the use of **(2*R*,3*S*)-7**. Based on the results of said selectivity screens, the observation of cytotoxicity in BET-dependent AML cell-lines above 1 μM and our FRAP dose-response experiments we recommend that, for cellular experiments, a dosage of 100–500 nM is optimal, to allow for total or near-total inhibition of L/V BET proteins while sparing the wild-type.

Although we show how the current system can be used to address biological questions, the investigation of more complex physiological and disease-relevant functions, for example comparing the genes regulated by each protein, will require the development of isogenic cell-lines and model organisms carrying the L/V mutation. Recent advances in the use of the CRISPR/cas9 system^58^ as a gene-editing technology presents an ideal opportunity to introduce single-point mutations into a variety of cell-lines and model organisms, without introducing exogenous BET genes that may not be regulated or post-translationally processed correctly.

Our optimized bumped compounds could also provide useful chemical tools for sophisticated or unconventional chemical genetics experiments. Ethyl-amide containing compounds (**16**, **17** & **21**) could be converted into L/V-selective alternatives to Bio-JQ1 (ref. 40) and a series of JQ1-based cross-linking compounds used for protein pulldown and fluorescence microscopy.^59^ The 1,4-benzodiazepine scaffold used in BET inhibitors has also been derivatized to create PROTAC degraders^32^–^35^,^37^ and bivalent inhibitors,^60^ and such modifications could be implemented in our bumped compounds.

Through optimizing our specific bump-&-hole system we believe we have revealed some observations relevant to the bump-&-hole technique in general. It is clear that any mutation introduced to target proteins must be very subtle, as even the relatively conservative L/A mutation had noticeable effects on BET bromodomain binding and function. Fortunately, major mutations and large ‘holes’ are not required for selectivity, as our L/V mutation allowed for over 100-fold selectivity despite the removal of only one methyl group. By association, the design of mutant-selective compounds should focus on minor modifications, and longer alkyl bumps can easily cause large drops in potency. Large bumps will also increase the molecular weight and log *P* of the inhibitor, leading to poorer DMPK properties. Care should be taken with regard to bump placement, as our compounds featured the bump on a flexible side-group which allowed for some residual WT binding. This could be prevented by locking the bump in place, as we do with the amide side-groups, or by placing the bump on a rigid part of the scaffold. Interestingly, we show the potential of introducing chemical modifications that are not located near the bump or mutation, as our best compounds contained a methoxy shift modification quite distant to our alkyl bump and L/V hole.

## Conclusions

In summary, we describe an iterative, step-wise and rational optimization of the bump-&-hole approach for allele-selective BET bromodomain inhibition, in both its biological and chemical aspects, which has led to the development of a more reliable and powerful system.

Through a three-stage process, several bumped analogues were identified with high potency, selectivity for the L/V mutant over WT bromodomains and desirable DMPK properties. This culminated in our selection of enantiomerically-pure **(2*R*,3*S*)-7** as a chemical probe targeting L/V BET bromodomains with ∼200 nM potency, >100-fold selectivity across the BET subfamily and displaying an excellent DMPK profile.

This orthogonal **(2*R*,3*S*)-7**:L/V inhibitor:mutant pair was validated through a number of *in vitro* and cellular experiments, and then utilized to answer a biological question, revealing that the BD1 of all BET proteins is more important to chromatin binding than the BD2, albeit to varying degrees. Interestingly, the BD2 of BRD4 was shown to still be essential for transcription (with NF-κB target genes) highlighting the importance of BD:non-histone protein interactions. We present this optimized bump-&-hole system as a powerful and reliable tool for investigating the biological role of the BET proteins and for more advanced target validation.

## Crystal structure PDB codes

BRD2 BD2 L383V apo (5O38), in complex with compounds **3** (5O39), **4** (5O3A), **5** (5O3B), **7** (5O3C), **8** (5O3D), **16** (5O3E), **17**(5O3F), **18** (5O3G) **21** (5O3H) and **24** (5O3I).

## Conflicts of interest

There are no conflicts of interest to declare.

## Supplementary Material

Supplementary information
