# Supplementary material for: Optimization of a “bump-and-hole” approach to allele-selective BET bromodomain inhibition
Source: Chem Sci. 2018 Jan 24;9(9):2452–68. doi: 10.1039/c7sc02536j (PMC5909127; doi:10.1039/c7sc02536j)
Supplement: Supplementary file 1 [file SC-009-C7SC02536J-s001.pdf]

Electronic Supplementary Material (ESI) for Chemical Science.
This journal is © The Royal Society of Chemistry 2018

Optimization of a "Bump-and-Hole" Approach to Allele-Selective BET Bromodomain Inhibition

A. C. Runcie[a]†, M. Zengerle[a]†, K.-H. Chan[a]†, A. Testa[a], L. van Beurden[a], M. G. J. Baud[a], O. Epemolu[a], L. C.J. Ellis[a], K. D. Read[a], V. Coulthard[b], A. Brien[b], A. Ciulli[a]*

[a] Division of Biological Chemistry and Drug Discovery, School of Life Sciences, University of Dundee, Dundee, Scotland, UK.

[b] Reach Separations Ltd, BioCity Nottingham, Nottingham, UK.

Supplementary Information

Supplementary Figures & Tables S2
X-Ray Crystallography: Data Collection & Refinement Statistics S20
Materials & Methods S22
Compound Synthesis & Characterization S26
Supplemental References S38

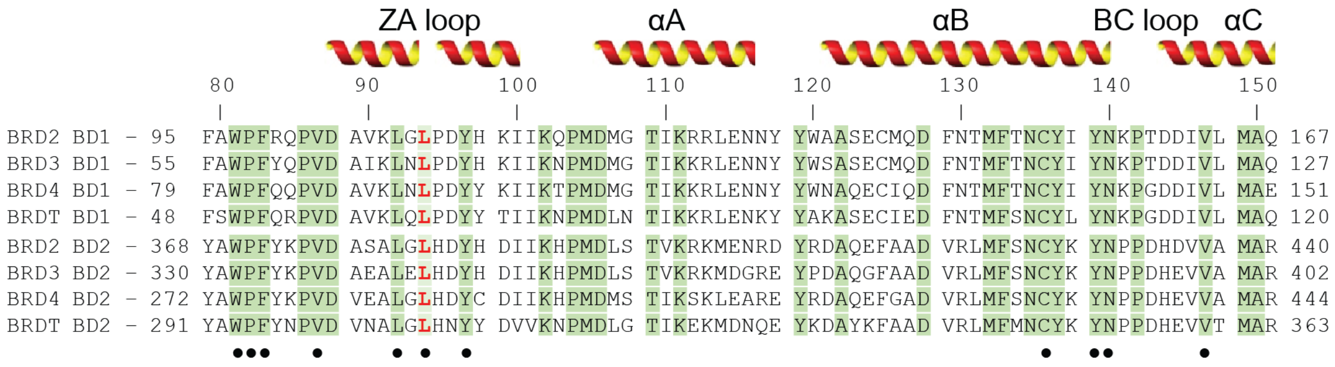

Figure S1 – BET Bromodomain Sequence alignment

Sequence alignment of the eight human BET bromodomains, with positions of α–helices and associated loops. Conserved residues highlighted in green, with mutated leucine in red. Conserved residues making direct contact with I-BET shown with black dot. Ruler numbering based on BRD4 BD1 sequence.

Bromodomain	Tm (°C) ±SE			ΔTm (°C) ±SE	
	WT	L/V	L/A	L/V	L/A
BRD2 BD1	45 ±0.0	43 ±0.6	45 ±0.5	-2 ±0.6	-1 ±0.6
BRD2 BD2	49 ±0.1	48 ±0.1	48 ±0.3	-1 ±0.1	-1 ±0.3
BRD3 BD1	46 ± 0.1	43 ±0.2	44 ±0.1	-3 ±0.2	-2 ±0.1
BRD3 BD2	44 ±0.1	45 ±0.9	44 ±0.0	1 ±0.9	0 ±0.1
BRD4 BD1	43 ±0.2	41 ±0.2	44 ±0.2	-2 ±0.3	0 ±0.3
BRD4 BD2	46 ±0.1	45 ±0.3	43 ±0.1	-1 ±0.3	-4 ±0.1
BRDT BD1	45 ±0.2	45 ±0.1	43 ±0.2	1 ±0.2	-1 ±0.3
BRDT BD2	44 ±0.1	42 ±1.6	47 ±0.1	-2 ±1.6	3 ±0.2

Table S1 – Thermal Stability of BET Bromodomain Constructs

Differential Scanning Fluorimetry –derived melting points of WT, L/V and L/A BET bromodomains. Mean and standard error of three replications.

H4 K5,8ac	BRD2 BD1		BRD2 BD2		BRD3 BD1		BRD3 BD2		BRD4 BD1		BRD4 BD2		BRDT BD1	
	WT	L/V	WT	L/V	WT	L/V	WT	L/V	WT	L/V	WT	L/V	WT	L/V
Kd (μM)	31	42	91	138	18	22	105	154	8	10	83	198	23	40
SE	2	3	19	16	1	1	12	16	1	1	2	22	4	8
ΔH (kcal/mol)	-8.8	-9.8	-1.7	-1.4	-9.4	-10.7	-6.7	-11.6	-10.5	-10.9	-4.1	-5.6	-14.4	-16.9
SE	0.2	0.5	0.2	0.2	0.1	0.2	1.1	5.7	0.1	0.1	0.1	0.9	1.4	3.0
N	2.1	1.8	2.3	2.0	2.0	1.7	1.2	0.5	1.7	1.7	1.4	1.6	1.3	1.1
SE	0.04	0.06	1.14	0.17	0.02	0.02	0.18	0.22	0.02	0.01	0.02	0.21	0.10	0.16

H4 K5,8, 12,16ac	BRD2 BD1		BRD2 BD2		BRD3 BD1		BRD3 BD2		BRD4 BD1		BRD4 BD2		BRDT BD1	
	WT	L/V	WT	L/V	WT	L/V	WT	L/V	WT	L/V	WT	L/V	WT	L/V
Kd (μM)	16	34	133	169	20	36	63	128	9	13	83	138	15	33
SE	1	1	7	12	1	3	6	7	1	1	3	10	1	4
ΔH (kcal/mol)	-11.9	-15.7	-6.3	-3.8	-9.1	-10.5	-9.3	-7.0	-9.7	-9.8	-8.3	-7.3	-14.8	-14.9
SE	0.1	0.2	0.4	0.3	0.1	0.3	0.9	1.0	1.8	0.1	0.2	0.4	0.4	1.0
N	1.1	1.2	1.1	1.5	2.3	2.0	0.9	1.0	1.8	1.6	0.9	1.1	1.3	1.1
SE	0.01	0.01	0.05	0.08	0.01	0.03	0.08	(FIX)	0.02	0.02	0.02	0.04	0.02	0.06

Table S2 – Effect of L/V Mutation on BRD:Peptide Affinity

Results of ITC titrations of di- and tetra-acetylated H4 peptides titrated into WT and L/V BET bromodomains.

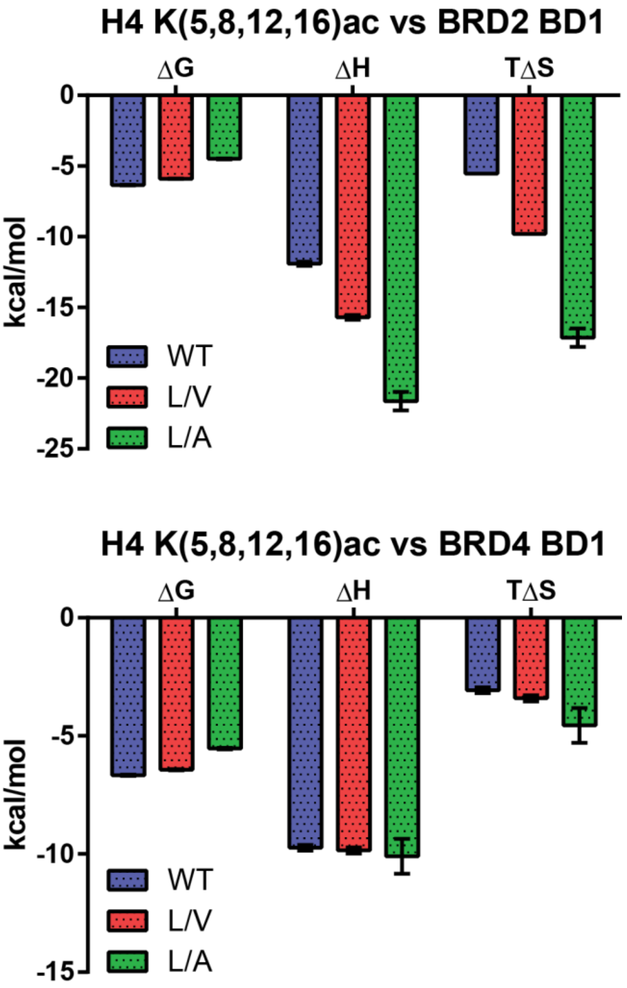

Figure S2 – Effect of L/V Mutation on Binding Energies of BRD:Peptide Interactions

Thermodynamic measurements of tetra-acetylated H4 peptide titrated into WT and mutant BET bromodomains.

Peptide	BRD2 BD1 WT	BRD2 BD1 LV	BRD2 BD2 WT	BRD2 BD2 LV	BRD3 BD1 WT	BRD3 BD1 LV	BRD3 BD2 WT	BRD3 BD2 LV	BRD4 BD1 WT	BRD4 BD1 LV	BRD4 BD2 WT	BRD4 BD2 LV	BRDT BD1 WT	BRDT BD1 LV	BRDT BD2 WT	BRDT BD2 LV
H4 1-21	0.62	0.21	0.07	-0.03	0.14	-0.01	0.08	0.04	0.05	0.03	0.00	0.19	-0.23	0.00	0.06	-0.04
H4 1-21 K5ac	0.53	0.21	0.18	0.06	0.23	0.10	0.23	0.21	0.14	0.18	0.16	0.29	-0.11	0.07	0.19	-0.03
H4 1-21 K8ac	0.44	0.17	0.15	0.03	0.17	0.08	0.21	0.12	0.10	0.19	0.11	0.22	-0.17	0.03	0.09	-0.04
H4 1-21 K12ac	0.56	0.23	0.23	0.06	0.26	0.14	0.26	0.20	0.21	0.37	0.20	0.27	-0.01	0.12	0.13	-0.02
H4 1-21 K16ac	0.26	0.08	0.02	-0.02	0.09	0.02	0.09	0.06	0.04	0.10	0.03	0.15	-0.17	0.01	0.02	-0.04
H4 1-21 K5acK8ac	2.70	1.40	0.54	0.16	3.77	2.18	0.54	0.31	2.74	2.12	0.38	0.53	1.00	0.59	0.29	0.11
H4 1-21 K5K8ac	0.46	0.25	0.37	0.04	0.40	0.23	0.51	0.32	0.33	0.55	0.37	0.32	0.09	0.15	0.07	0.02
H4 1-21 K5K16ac	0.16	0.11	0.16	0.06	0.16	0.12	0.24	0.18	0.12	0.32	0.17	0.18	-0.05	0.07	0.07	-0.01
H4 1-21 K8K12ac	0.49	0.28	0.37	0.07	0.49	0.34	0.33	0.24	0.42	0.68	0.33	0.28	0.13	0.18	0.10	0.03
H4 1-21 K8K16ac	0.15	0.12	0.16	0.07	0.17	0.15	0.23	0.16	0.13	0.37	0.17	0.17	-0.05	0.07	0.11	0.00
H4 1-21 K12K16ac	0.52	0.29	0.29	0.09	0.66	0.43	0.32	0.20	0.59	0.82	0.26	0.24	0.17	0.17	0.08	0.03
H4 1-21 K5K8K12ac	3.38	2.01	1.10	0.33	3.93	2.60	0.89	0.42	3.12	2.72	0.65	0.64	1.43	0.86	0.31	0.17
H4 1-21 K8K12K16ac	1.06	0.75	0.62	0.18	1.26	0.90	0.53	0.29	1.22	1.33	0.38	0.37	0.51	0.43	0.18	0.08
H4 1-21 K5K12K16ac	0.79	0.51	0.56	0.18	0.99	0.66	0.57	0.38	0.81	1.20	0.43	0.35	0.38	0.30	0.15	0.05
H4 1-21 K5K8K16ac	2.51	1.54	0.69	0.21	3.65	2.28	0.62	0.31	2.88	2.37	0.35	0.49	1.10	0.62	0.20	0.14
H4 1-21 K5K8K12K16ac	3.31	2.12	1.12	0.29	4.34	2.86	0.80	0.36	3.11	3.05	0.55	0.56	1.61	0.95	0.26	0.23
H4 9-29	0.04	0.04	0.01	-0.02	0.07	0.04	0.01	0.06	0.03	0.05	0.02	0.04	-0.09	0.01	0.03	0.00
H4 9-29 K20ac	0.11	0.18	0.26	0.17	0.10	0.14	0.34	0.46	0.13	0.38	0.22	0.25	0.11	0.33	0.06	0.11
H4 9-29 K16ac	0.05	0.06	0.02	0.01	0.04	0.05	0.05	0.06	0.03	0.09	0.03	0.03	0.01	0.04	0.01	0.02
H4 9-29 K12K16ac	0.36	0.47	0.27	0.12	0.40	0.41	0.23	0.18	0.23	0.63	0.18	0.13	0.23	0.32	0.06	0.10
H4 9-29 K12K16K20ac	0.50	0.90	0.48	0.23	0.53	0.66	0.35	0.32	0.33	1.07	0.22	0.23	0.33	0.68	0.06	0.17
H2a 1-21	0.45	0.09	0.04	-0.02	0.09	0.03	0.01	0.08	0.03	0.07	0.02	0.21	-0.14	0.01	0.04	-0.03
H2a 1-21 K5ac	0.54	0.23	0.17	0.04	0.22	0.04	0.18	0.17	0.14	0.17	0.16	0.32	-0.12	0.06	0.11	-0.02
H2a 1-21 K9ac	0.50	0.20	0.20	0.03	0.22	0.03	0.16	0.12	0.11	0.13	0.13	0.25	-0.16	0.04	0.10	-0.03
H2a 1-21 K13ac	0.36	0.13	0.07	0.01	0.16	0.05	0.03	0.08	0.07	0.08	0.04	0.20	-0.12	0.04	0.09	-0.02
H2a 1-21 K15ac	0.33	0.10	0.07	0.00	0.15	0.04	0.04	0.07	0.06	0.07	0.04	0.17	-0.15	0.03	0.08	-0.03
H2a 1-21 K5K9ac	0.24	0.12	0.30	0.10	0.22	0.13	0.34	0.24	0.16	0.28	0.28	0.31	-0.07	0.08	0.12	0.01
H2a 1-21 K5K13ac	0.10	0.07	0.14	0.03	0.17	0.09	0.20	0.17	0.11	0.18	0.15	0.19	-0.11	0.05	0.08	0.00
H2a 1-21 K5K15ac	0.12	0.07	0.07	0.09	0.14	0.07	0.20	0.17	0.09	0.19	0.07	0.21	-0.10	0.05	0.07	-0.01
H2a 1-21 K9K13ac	0.18	0.09	0.17	0.04	0.22	0.11	0.21	0.07	0.13	0.24	0.18	0.17	-0.09	0.06	0.09	0.00
H2a 1-21 K9K15ac	0.16	0.12	0.29	0.09	0.22	0.14	0.29	0.18	0.16	0.29	0.27	0.21	-0.03	0.06	0.11	0.02
H2a 1-21 K13K15ac	0.10	0.04	0.04	-0.01	0.13	0.06	0.05	0.07	0.05	0.10	0.06	0.09	-0.12	0.03	0.04	-0.02
H2a 1-21 K5K9K13ac	0.02	0.05	0.00	0.03	0.01	0.06	0.10	0.04	0.01	0.05	0.05	0.03	0.07	0.05	0.03	0.07
H2a 1-21 K5K13K15ac	0.12	0.08	0.17	0.06	0.15	0.11	0.20	0.17	0.13	0.23	0.17	0.15	0.02	0.05	0.03	0.00
H2a 1-21 K9K13K15ac	0.20	0.13	0.30	0.08	0.30	0.11	0.19	0.12	0.18	0.31	0.21	0.13	-0.03	0.06	0.12	-0.01
H2a 1-21 K5K9K15ac	0.19	0.13	0.18	0.15	0.32	0.13	0.40	0.20	0.22	0.35	0.35	0.26	-0.02	0.08	0.11	-0.02
H2a 1-21 K5K9K13K15ac	0.30	0.23	0.51	0.15	0.35	0.28	0.40	0.23	0.29	0.49	0.35	0.23	0.14	0.12	0.12	0.05
H2b 1-21	0.02	0.00	0.00	0.00	0.08	0.02	-0.02	0.04	0.01	0.01	0.02	0.04	-0.14	0.02	0.04	-0.02
H2b 1-21 K5ac	0.01	0.02	0.06	0.07	0.06	0.05	-0.02	0.06	0.02	0.06	0.02	0.02	-0.03	0.03	0.06	0.01
H2b 1-21 K11ac	0.00	0.00	0.00	0.01	0.00	0.02	0.00	0.04	0.01	0.01	0.01	0.02	-0.04	0.02	0.02	-0.01
H2b 1-21 K12ac	0.01	0.02	0.00	0.00	0.02	0.04	0.03	0.06	0.02	0.05	0.03	0.02	-0.01	0.02	0.04	0.01
H2b 1-21 K15ac	0.01	0.04	0.00	0.01	0.03	0.06	0.05	0.06	0.02	0.08	0.02	0.03	0.00	0.04	0.04	0.01
H2b 1-21 K5K11K16ac	0.04	0.04	0.02	0.03	0.04	0.08	0.04	0.04	0.03	0.10	0.03	0.03	0.04	0.04	0.04	0.02
H2b 1-21 k5k12AC	0.02	0.01	0.00	0.02	0.00	0.03	0.02	0.03	0.01	0.02	0.02	0.02	0.00	0.03	0.01	0.00
H2b 1-21 K5K15ac	0.01	0.03	0.01	0.01	0.02	0.06	0.06	0.04	0.02	0.08	0.02	0.03	0.01	0.04	0.02	0.01
H2b 1-21 K5K16ac	0.02	0.03	0.01	0.02	0.04	0.05	0.03	0.03	0.02	0.09	0.02	0.02	0.03	0.03	0.00	0.01
H2b 1-21 K11K12ac	0.03	-0.02	0.06	0.01	0.03	-0.02	0.01	0.00	0.02	0.00	0.01	0.00	-0.02	0.00	0.00	-0.01
H2b 1-21 K11K15ac	0.04	0.03	0.11	0.05	0.07	0.02	0.04	0.01	0.03	0.07	0.02	0.01	0.00	0.02	0.01	0.00
H2b 1-21 K11K16ac	0.04	0.05	0.03	0.04	0.00	0.05	0.04	0.03	0.03	0.08	0.02	0.02	0.02	0.03	0.03	0.01
H2b 1-21 K5K11K12ac	0.03	0.01	0.05	0.03	0.03	0.04	0.04	0.03	0.02	0.05	0.02	0.02	0.01	0.01	0.03	0.01
H2b 1-21 K5K15K16ac	0.03	0.06	0.06	0.03	0.04	0.06	0.03	0.03	0.04	0.09	0.03	0.03	0.04	0.04	0.02	0.02
H2b 1-21 K5K15K16ac	0.26	0.12	0.17	0.03	0.50	0.20	0.06	0.03	0.17	0.17	0.03	0.03	0.13	0.06	0.04	0.03
H2b 1-21 K11K15K16ac	0.02	0.04	0.02	0.00	0.05	0.08	0.04	0.03	0.03	0.08	0.02	0.02	0.04	0.05	0.02	0.02
H2b 1-21 K12K15K16ac	0.47	0.17	0.20	0.04	1.02	0.30	0.07	0.02	0.45	0.34	0.05	0.03	0.30	0.10	0.03	0.02
H2b 1-21 K11K12K15ac	0.17	0.10	0.13	0.04	0.30	0.16	0.06	0.03	0.11	0.15	0.03	0.03	0.11	0.06	0.04	0.03
H2b 1-21 K11K12K16ac	0.04	0.04	0.01	0.02	0.06	0.07	0.05	0.04	0.04	0.07	0.02	0.03	0.05	0.04	0.02	0.02
H2b 1-21 K5K11K12K15K16ac	0.07	0.12	0.01	0.06	0.01	0.05	0.09	0.04	0.03	0.03	0.03	0.01	0.07	0.09	0.01	0.05
H2b 13-33	0.73	0.14	0.09	-0.02	0.18	0.06	0.02	0.11	0.05	0.08	0.02	0.25	-0.25	0.02	0.08	-0.04
H2b 13-33 K20ac	1.09	0.39	0.18	0.01	0.19	0.04	0.04	0.10	0.09	0.09	0.03	0.29	-0.28	0.03	0.12	-0.02
H2b 13-33 K23ac	0.88	0.35	0.14	-0.01	0.20	0.02	0.07	0.09	0.06	0.05	0.02	0.26	-0.27	0.02	0.10	-0.05
H2b 13-33 K24ac	0.83	0.26	0.11	0.00	0.14	0.06	0.02	0.10	0.07	0.10	0.02	0.26	-0.24	0.03	0.11	-0.03
H2b 13-33 K20K23K24ac	0.43	0.18	0.23	0.02	0.23	0.09	0.08	0.10	0.09	0.12	0.07	0.19	-0.21	0.05	0.10	-0.05
H3 1-21	0.09	0.02	0.01	0.01	0.08	0.03	-0.01	0.06	0.03	0.02	0.01	0.10	-0.10	0.02	0.04	-0.02
H3 1-21 K4ac	0.02	0.02	0.01	0.00	0.10	0.05	0.03	0.07	0.03	0.07	0.04	0.06	-0.13	0.03	0.04	-0.01
H3 1-21 K9ac	0.02	0.01	-0.01	0.00	0.07	0.05	0.03	0.06	0.02	0.04	0.02	0.05	-0.11	0.02	0.04	0.00
H3 1-21 K14ac	0.07	0.03	0.06	0.03	0.12	0.06	0.10	0.10	0.04	0.09	0.10	0.10	-0.09	0.03	0.05	0.00
H3 1-21 K4K9ac	0.04	0.05	0.05	0.02	0.07	0.09	0.06	0.09	0.06	0.13	0.06	0.06	0.00	0.05	0.06	0.02
H3 1-21 K4K14ac	0.07	0.04	0.08	0.03	0.10	0.07	0.10	0.12	0.05	0.12	0.11	0.08	-0.02	0.04	0.03	0.00
H3 1-21 K9K14ac	0.05	0.04	0.04	0.02	0.04	0.08	0.05	0.05	0.03	0.10	0.03	0.03	0.05	0.03	0.03	0.02
H3 1-21 K4K9K14ac	0.11	0.07	0.10	0.04	0.09	0.09	0.15	0.10	0.07	0.17	0.10	0.07	0.06	0.05	0.05	0.02
H3 11-31	0.01	-0.03	0.05	0.01	0.11	-0.01	-0.03	0.05	0.02	0.02	0.00	0.04	-0.17	-0.01	0.04	-0.03
H3 11-31 K14ac	0.03	-0.02	0.04	0.00	0.09	0.01	0.00	0.05	0.01	0.03	0.04	0.03	-0.07	-0.01	0.02	-0.03
H3 11-31 K18ac	0.04	0.03	0.11	0.06	0.07	0.08	0.15	0.14	0.06	0.16	0.19	0.10	-0.01	0.04	0.13	0.04
H3 11-31 K23ac	0.11	0.08	0.03	0.00	0.14	0.04	0.01	0.09	0.05	0.06	0.02	0.12	-0.22	0.01	0.08	-0.03
H3 11-31 K27ac	0.01	0.01	0.01	0.00	0.04	0.04	0.00	0.07	0.02	0.06	0.03	0.03	-0.02	0.01	0.04	0.00
H3 11-31 K14K18ac	0.06	0.04	0.13	0.06	0.08	0.10	0.18	0.12	0.05	0.15	0.15	0.09	0.03	0.05	0.11	0.05
H3 11-31 K14K23ac	0.04	0.02	0.03	0.02	0.03	0.06	0.07	0.05	0.04	0.07	0.04	0.04	0.03	0.02	0.03	0.02
H3 11-31 K14K27ac	0.05	0.02	0.08	0.07	0.03	0.06	0.07	0.05	0.02	0.09	0.05	0.04	0.03	0.03	0.03	0.02
H3 11-31 K18K23ac	0.06	0.05	0.10	0.05	0.05	0.09	0.13	0.07	0.05	0.15	0.13	0.07	0.05	0.05	0.06	0.03
H3 11-31 K18K27ac	0.05	0.04	0.13	0.06	0.08	0.12	0.19	0.12	0.07	0.18	0.18	0.11	0.06	0.07	0.10	0.06
H3 11-31 K23K27ac	0.03	0.04	0.01	0.02	0.02	0.06	0.04	0.04	0.02	0.08	0.02	0.03	0.05	0.03	0.02	0.02
H3 11-31 K14K23K27ac	0.10	0.06	0.07	0.02	0.07	0.10	0.08	0.05	0.05	0.11	0.05	0.03	0.08	0.05	0.03	0.03
H3 11-31 K18K23K27ac	0.16	0.13	0.30	0.11	0.14	0.10	0.24	0.09	0.12	0.22	0.23	0.10	0.08	0.11	0.11	0.06
H3 11-31 K14K18K27ac	0.11	0.06	0.14	0.05	0.12	0.06	0.16	0.05	0.07	0.15	0.15	0.06	0.03	0.03	0.08	0.03
H3 11-31 K14K18K23K27ac	0.16	0.13	0.29	0.10	0.14	0.13	0.18	0.09	0.10	0.20	0.18	0.08	0.10	0.09	0.11	0.06
H3 23-43	0.10	0.08	0.05	0.02	0.13	0.07	0.02	0.09	0.05	0.05	0.02	0.12	-0.22	-0.01	0.06	-0.04
H3 23-43 K36ac	0.04	0.06	0.11	0.03	0.11	0.07	0.19	0.14	0.06	0.08	0.11	0.09	-0.08	0.04	0.07	-0.01
H3 23-43 K37ac	0.02	0.02	0.11	0.00	0.11	0.09	0.05	0.09	0.04	0.06	0.09	0.06	-0.08	-0.01	0.08	-0.02
H3 23-43 K36K37ac	0.05	0.04	0.23	0.04	0.05	0.07	0.18	0.11	0.05	0.08	0.16	0.07	-0.01	0.04	0.10	0.01
H3 23-43 K27ac	0.02	0.02	0.01	0.00	0.08	0.08	0.01	0.08	0.03	0.07	0.04	0.05	-0.09	0.00	0.03	-0.02
H3 23-43 K27K36ac	0.06	0.05	0.17	0.06	0.07	0.10	0.23	0.14	0.07	0.14	0.11	0.08	0.00	0.06	0.08	0.02
H3 23-43 K27K37ac	0.05	0.03	0.19	0.03	0.07	0.09	0.16	0.10	0.06	0.10	0.14	0.09	-0.01	0.00	0.08	0.01
H3 23-43 K27K36K37ac	0.09	0.07	0.31	0.08	0.06	0.09	0.22	0.07	0.07	0.12	0.17	0.08	0.05	0.05	0.11	0.03

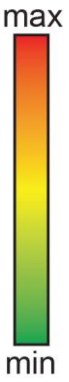

Figure S3 – BLI Screen of WT & L/V BET Bromodomains against Acetylated Histone Library

BLI binding profiles of WT and L/V BET bromodomains tested against acetylated histone peptide library. Average response of two biocytin negative controls subtracted, to account for non-specific binding. Responses color-coded to maximum and minimum response of each construct.

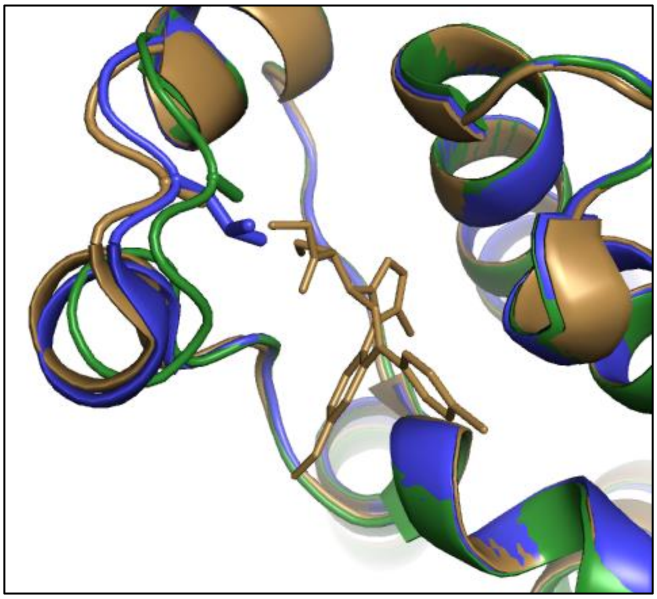

Figure S4 – Crystallographic Analysis of ZA Loop during Binding

Aligned X-ray crystal structures of BRD2 BD2 L/A apo (green) (4QEU) and bound to ET (brown) (4QEW), as well as BRD2 BD2 WT bound to an acetylated H4 peptide (blue) (2E3K). Bound peptide not shown. Mutated leucine/alanine side-chain highlighted.

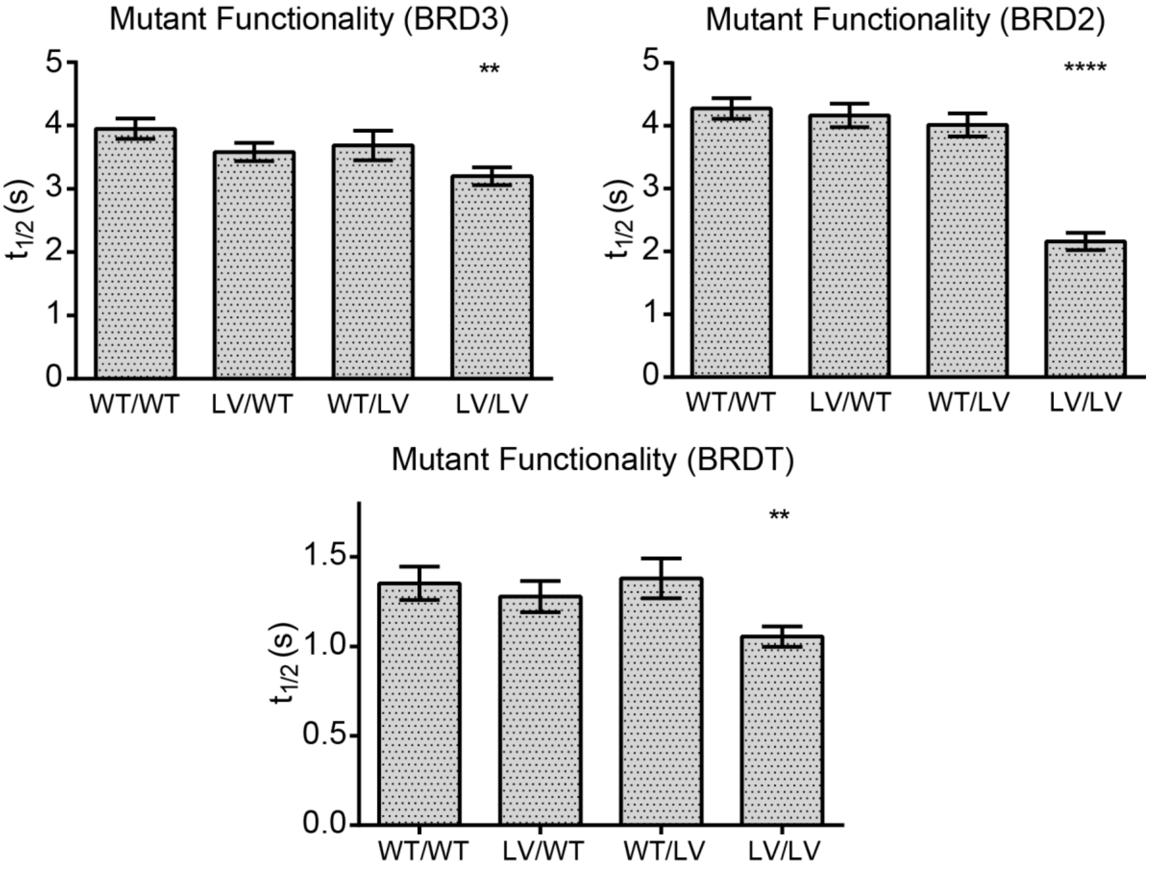

Figure S5 – Effect of L/V Mutation on BET Protein Construct's FRAP Recovery Times

Recovery times of GFP-labelled BET bromodomain constructs in FRAP assay, following 0.5s laser bleach event, at 2 µM SAHA. Each bar is mean and SE of ~50 U2OS cells tested over two separate experiments. Statistical significance determined with two-tailed *t* tests: ns P>0.05; * P ≤ 0.05, ** P≤0.01, *** P≤0.001, **** P<0.0001.

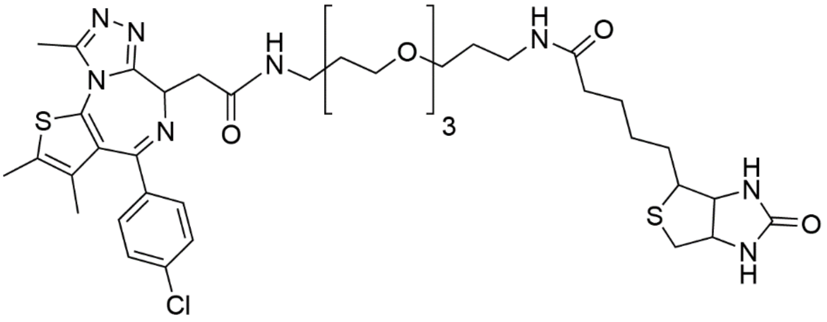

	Positive	Negative
**Mean**	1.7x10^6	1.7x10^4
**SD**	1.3x10^5	2.1x10^3
**S/N**	102	
**Z'**	0.77	

Figure S6 – Structure of Biotinylated-JQ1 and Associated Assay Robustness

Structure of biotinylated JQ1. Z' calculated using BRD2 BD2 WT with 0 and 300 µM JQ1 (positive and negative controls) (12 replicates).

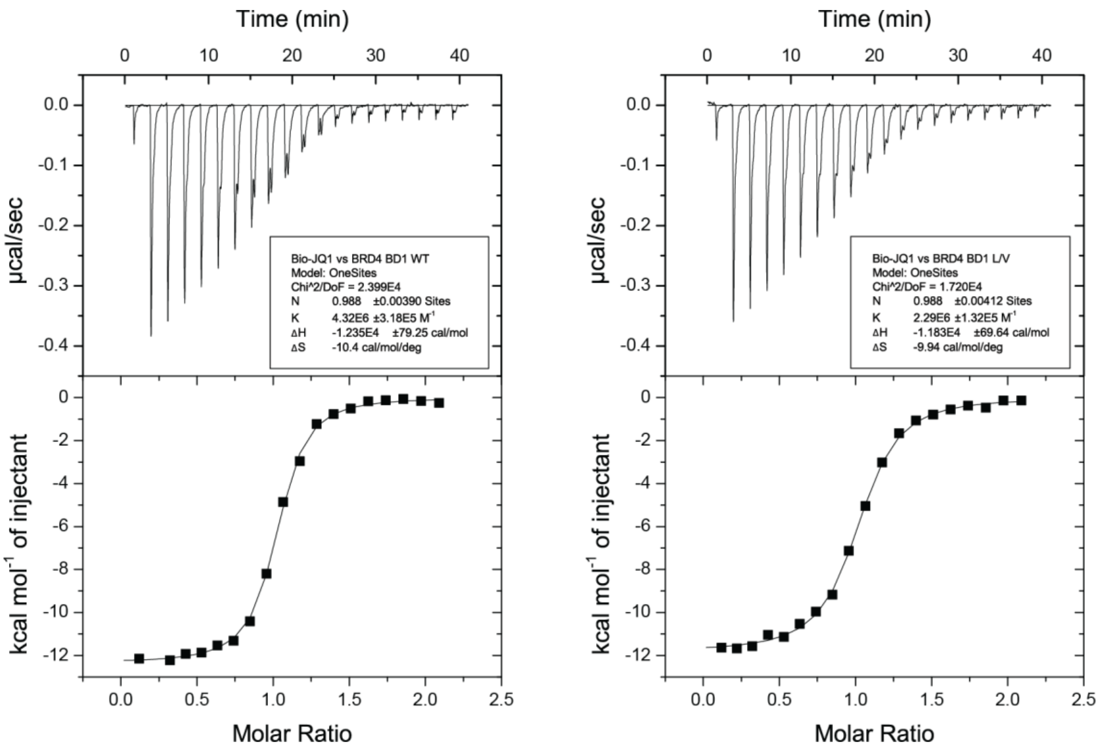

Figure S7 – ITC Titrations of Biotinylated-JQ1

ITC Titrations of biotinylated JQ1 into WT and L/V BRD4 BD1.

Bio-JQ1	BRD2 BD1		BRD2 BD2		BRD3 BD1		BRD3 BD2		BRD4 BD1		BRD4 BD2		BRDT BD1		BRDT BD2	
	WT	L/V	WT	L/V	WT	L/V	WT	L/V	WT	L/V	WT	L/V	WT	L/V	WT	L/V
EC_{50} (nM)	1.4	3.2	2.3	1.8	0.9	1.2	1.9	4.0	1.0	2.0	1.3	2.9	3.2	4.0	3.1	4.9
95% C.I.	1.0-2.6	1.2-2.1	1.6-3.2	1.6-2.2	0.6-1.3	1.1-1.4	1.7-2.3	3.7-4.4	0.7-1.5	1.6-2.6	0.8-2.2	2.7-3.8	2.7-3.7	3.7-4.4	2.4-3.9	3.9-6.1
LV/WT	2.3		0.8		1.3		2.1		2.0		2.2		1.3		1.6	

Table S3 – AlphaLISA Titrations of Biotinylated-JQ1

Results of dose-response curves of JPB titrated against 100 nM WT and L/V BET bromodomains. Data points after the 'hook' point excluded from analysis

**Compound**	**AlphaLISA pIC_{50} Value**															
	**BRD2 BD1**		**BRD2 BD2**		**BRD3 BD1**		**BRD3 BD2**		**BRD4 BD1**		**BRD4 BD2**		**BRDT BD1**		**BRDT BD2**	
	**WT**	**L/V**	**WT**	**L/V**	**WT**	**L/V**	**WT**	**L/V**	**WT**	**L/V**	**WT**	**L/V**	**WT**	**L/V**	**WT**	**L/V**
(+)JQ1	6.4	6.5	6.5	6.5	6.8	6.6	6.5	6.4	6.6	6.5	6.5	6.4	6.6	6.0	6.3	6.1
(-)JQ1	4.8	4.8	4.8	4.4	5.0	5.0	4.6	4.4	4.9	4.6	4.7	4.5	4.7	4.6	4.6	4.6
I-BET	6.2	6.1	6.3	6.7	6.7	6.9	6.6	6.3	6.6	6.3	6.5	6.5	6.3	6.0	6.3	6.3
1	6.5	6.6	6.5	6.5	6.9	7.2	6.6	6.3	6.9	6.6	6.4	6.6	6.3	6.2	6.6	6.0
2	5.7	5.8	5.6	5.7	6.2	6.3	5.6	6.0	6.0	6.0	5.6	5.6	5.7	5.8	5.4	5.5
3	5.7	7.2	6.0	7.3	6.6	7.5	6.3	7.5	6.2	7.4	6.1	7.3	5.7	7.3	5.9	7.1
4	4.9	6.5	5.0	6.8	5.2	7.0	5.0	7.0	5.1	6.6	4.9	6.6	4.8	6.3	4.8	6.2
5	5.0	6.0	5.0	6.8	5.4	6.9	5.0	6.9	5.1	6.5	5.0	6.6	4.7	5.7	4.9	6.2
6	4.5	5.0	<4	5.0	4.7	5.2	4.7	5.0	4.6	5.1	4.2	4.9	4.5	4.6	4.9	6.2
7	5.5	6.7	5.1	6.5	5.8	7.0	5.3	7.0	5.5	6.7	5.2	6.7	5.3	6.6	5.1	6.2
8	5.0	6.5	5.4	6.7	5.6	6.9	5.4	7.1	5.8	6.9	5.5	6.7	4.8	6.2	5.4	6.4
9	4.3	5.4	4.4	5.9	4.5	5.8	4.3	6.0	4.4	5.5	4.2	5.5	4.2	5.2	4.3	5.3
16	4.9	6.4	5.3	6.6	5.7	7.0	5.4	7.1	5.4	6.6	5.3	6.6	4.9	6.2	5.1	6.4
17	4.2	5.5	4.6	5.9	4.5	6.3	4.4	6.1	4.3	5.7	4.2	5.7	4.2	4.9	4.1	5.3
18	4.3	5.6	4.3	6.0	4.5	6.1	4.3	5.9	4.4	5.9	4.2	5.7	4.1	5.3	4.1	5.3
19	4.9	6.0	4.6	6.0	5.3	6.4	4.9	6.3	4.9	5.9	4.6	5.9	4.4	5.4	4.6	5.5
20	4.5	5.7	4.8	5.8	4.7	6.3	4.6	5.8	4.6	5.9	4.5	5.5	4.4	5.2	4.4	5.2
21	4.4	5.7	4.8	6.0	5.3	6.3	4.8	6.3	4.6	6.0	4.5	5.9	4.3	5.6	4.6	5.8
22	<4	5.2	<4	5.1	4.2	5.5	<4	5.4	4.2	5.1	<4	4.9	<4	4.7	<4	4.7
23	5.3	5.8	5.3	6.0	5.6	6.4	5.3	6.1	5.3	6.1	5.2	5.8	5.2	5.6	5.2	5.6
24	5.3	5.9	5.1	6.3	5.4	6.5	5.2	6.1	5.3	6.2	5.4	6.0	5.1	5.7	5.0	5.8
25	4.4	5.0	4.7	4.9	4.7	5.3	4.6	5.0	4.8	5.1	4.6	4.7	4.6	4.9	4.4	4.9
26	5.1	5.6	5.2	5.8	5.5	5.9	5.3	5.9	5.2	5.8	5.0	5.5	5.0	5.4	5.0	5.6

Table S4- Results of AlphaLISA Assay Screen

Compound	AlphaLISA pIC_{50}			Plasma t½ (mins)	CL_{int} (ml/min/g)	P_e (nm/s)	CLogP	ITC pKd		
	WT	L/V	Δ					WT	L/V	Δ
(+) JQ1	6.6	6.5	-0.1	>180	7.5		4.8			
(-) JQ1	4.8	4.6	-0.2				4.8			
I-BET762	6.5	6.5	0.0	>180	1.3	25	2.8			
1	6.6	6.6	0.0	54	<0.5	149	3.3			
2	5.8	5.9	0.1	67	1.7		3.3			
3	6.2	7.4	1.2	>180	0.7	185	3.5			
4	5.0	6.8	1.7	>180	1.5	153	3.9	5.1	6.9	1.8
5	5.1	6.6	1.5	>180	6.7	127	3.9	5.1	6.6	1.5
6	4.5	5.0	0.6	>180	5.2		4.3			
7	5.4	6.8	1.4	>180	<0.5	158	3.5	4.5	6.6	2.0
8	5.5	6.8	1.4	>180	4.1	155	3.9	<4.2	6.4	>2.2
9	4.4	5.7	1.3	>180	9.4	136	3.9			
16	5.3	6.7	1.4	>180	1.2	26	3.1	5.6	6.9	1.3
17	4.4	5.9	1.5	>180	1.4	45	3.5	<4.2	6.0	>1.8
18	4.3	5.9	1.5	>180	2.6	52	3.5			
19	4.9	6.1	1.2	>180	>50		3.8			
20	4.6	5.8	1.2	>180	>50		4.1			
21	4.7	6.0	1.3	>180	1.5	40	3.1	4.5	6.3	1.8
22	<4.0	5.2		>180	2.1	59	3.5			
23	5.3	6.0	0.7	>180	30.7		4.8			
24	5.3	6.2	0.9	>180	39.7		4.9			
25	4.6	5.0	0.4	>180	31.0		4.8			
26	5.2	5.8	0.5	>180	39.4		4.9			

4.0 — 7.0

0.0 — 2.2

Figure S8. Color-Coded SAR

Data from table 1, with potency/selectivity data color-coded.

Compound		BRD2 BD1		BRD2 BD2		BRD3 BD1		BRD3 BD2		BRD4 BD1		BRD4 BD2	
		WT	L/V	WT	L/V	WT	L/V	WT	L/V	WT	L/V	WT	L/V
**4**	K_d (nM)	22000 ±9000	240 ±50	3300 ±1400	50 ±10	3300 ±1000	80 ±10	16000 ±8000	100 ±30	2500 ±1000	160 ±10	35000 ±10000	330 ±50
	ΔH (kcal/mol)	-5.3 ±0.9	-12.6 ±0.2	-1.2 ±0.1	-8.5 ±0.1	-4.9 ±0.4	-14.1 ±0.1	-3.8 ±1.4	-9.5 ±0.2	-2.6 ±0.2	-10.3 ±0.1	-3.4 ±0.5	-7.9 ±0.1
	N	1 (FIX)	0.96 ±0.01	0.91 ±0.07	0.85 ±0.01	0.81 ±0.05	0.94 ±0.00	0.85 ±0.20	0.94 ±0.01	0.99 ±0.06	0.87 ±0.00	1 (FIX)	0.82 ±0.01
**5**	K_d (nM)	8400 ±3400	470 ±100	5800 ±600	240 ±60	1500 ±600	270 ±30	23000 ±8000	310 ±60	9700 ±1800	180 ±10	16000 ±9000	170 ±20
	ΔH (kcal/mol)	-4.0 ±0.7	-10.3 ±0.2	-2.3 ±0.1	-5.9 ±0.1	-2.3 ±0.2	-11.3 ±0.1	-3.7 ±0.6	-8.5 ±0.2	-3.5 ±0.3	-7.4 ±0.0	-2.2 ±0.8	-6.4 ±0.5
	N	1.0 ±0.10	0.87 ±0.01	0.75 ±0.02	1.16 ±0.01	0.85 ±0.05	0.84 ±0.01	1 (FIX)	0.78 ±0.01	1.10 ±0.05	0.88 ±0.00	0.98 ±0.20	0.89 ±0.01
**16**	K_d (nM)	2200 ±900	120 ±20	2400 ±500	190 ±20	1600 ±300	60 ±10	1500 ±300	130 ±20	9400 ±1100	160 ±20	1700 ±300	80 ±10
	ΔH (kcal/mol)	-4.5 ±0.4	-16.3 ±0.2	-3.9 ±0.2	-9.9 ±0.1	-8.8 ±0.3	-19.0 ±0.2	-6.0 ±0.2	-12.9 ±0.2	-10.2 ±0.5	-9.5 ±0.1	-3.6 ±0.1	-8.6 ±0.1
	N	0.94 ±0.05	0.75 ±0.01	0.65 ±0.03	0.62 ±0.00	0.81 ±0.02	0.64 ±0.00	0.88 ±0.03	0.74 ±0.01	0.96 ±0.03	0.92 ±0.01	0.81 ±0.02	0.83 ±0.00
**17**	K_d (nM)	>60000	960 ±180	>60000	1300 ±200	>60000	740 ±130	>60000	610 ±90	>60000	1600 ±300	>60000	1000 ±200
	ΔH (kcal/mol)		-10.2 ±0.3		-5.3 ±0.1		-9.4 ±0.2		-4.7 ±0.1		-5.9 ±0.2		-3.7 ±0.1
	N		0.97 ±0.02		0.94 ±0.01		1.07 ±0.02		0.68 ±0.01		0.99 ±0.02		0.99 ±0.02
**21**	K_d (nM)	>60000	990 ±50	7400 ±600	230 ±50	7400 ±600	410 ±20	11000 ±3000	800 ±50	39000 ±6000	370 ±50	>60000	320 ±20
	ΔH (kcal/mol)		-8.8 ±0.1	-5.1 ±0.2	-4.8 ±0.1	-5.1 ±0.2	-10.7 ±0.1	-4.5 ±1.3	-6.7 ±0.1	-3.3 ±0.3	-5.9 ±0.1		-4.4 ±0.0
	N		0.97 ±0.01	0.86 ±0.02	0.84 ±0.03	0.86 ±0.02	0.92 ±0.00	0.46 ±0.10	0.90 ±0.01	1 (FIX)	0.88 ±0.01		1.06 ±0.01

Table S5 – Results of ITC Profiling

Results of ITC titrations of compound titrated into BET bromodomains. For very weak interactions N value fixed to 1.0.

**7**	L/V	2.1	2.2	3.1	3.2	4.1	4.2
WT	Kd (nM)	310	330	140	230	140	980
2.1	32000	100	100	230	140	230	30
2.2	>60000	>190	>180	>430	>260	>430	>60
3.1	12000	40	40	90	50	90	10
3.2	34000	110	100	240	150	240	40
4.1	28000	90	90	200	120	200	30
4.2	33000	110	100	240	140	240	30

**8**	L/V	2.1	2.2	3.1	3.2	4.1	4.2
WT	Kd (nM)	340	300	270	410	530	740
2.1	>60000	>180	>200	>230	>150	>110	>80
2.2	>60000	>180	>200	>230	>150	>100	>80
3.1	29000	90	100	110	70	60	40
3.2	>60000	>180	>200	>230	>150	>110	>80
4.1	>60000	>180	>200	>230	>150	>100	>80
4.2	>60000	>180	>200	>230	>150	>100	>80

Table S6. Selectivity Plots of 9-ME & 9-ET

Results are means of three consistent ITC titrations of ligand into WT and L/V BET bromodomains. For some weak interactions N was fixed to 1

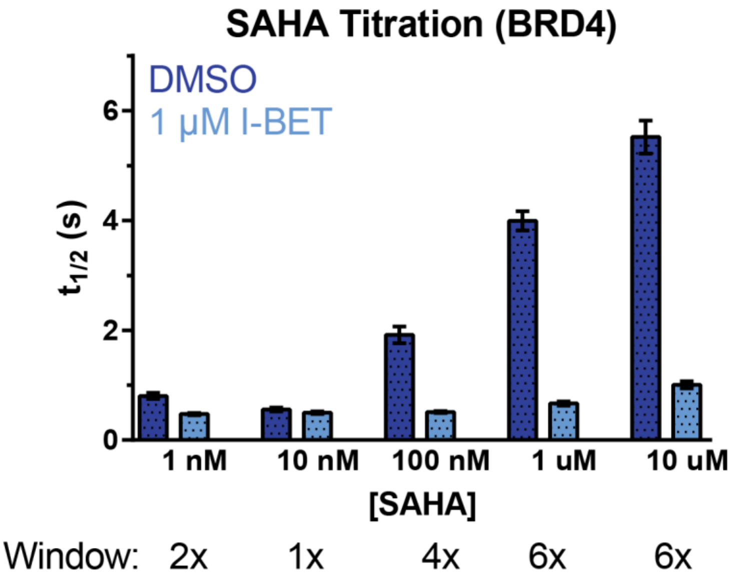

Figure S9 – Effects of SAHA treatment on FRAP assay window.

Recovery times of GFP-labelled BRD4 WT/WT in FRAP assay, following 0.5s laser bleach event, at a range of SAHA concentrations. Each bar is mean and SE of ~30 U2OS cells.

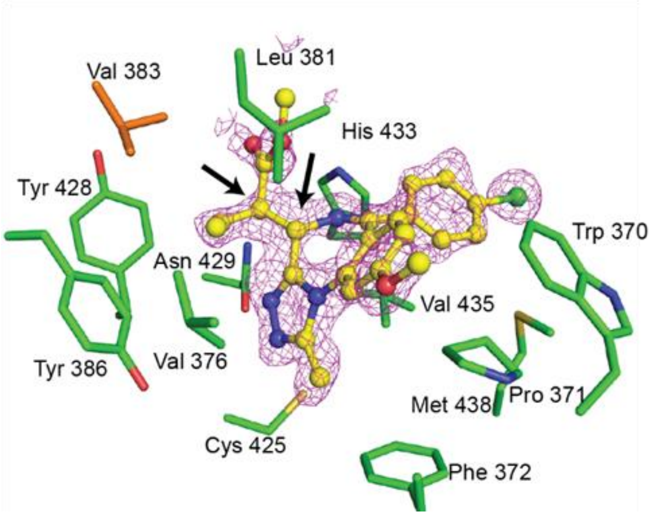

Figure S10 – 2R,3S is the Potent Enantiomer that Binds to BET Bromodomains

X-ray structure of BRD2 BD2 (L/V) (green, with mutant valine in orange) co-crystallised with (2R,3S)-8 (yellow). Fo-Fc ligand omit map (magenta mesh surface, contour: 2 sigma) shows clearly defined electron density around chiral centres of (2R,3S)-8 (marked with arrows).

BRD4 BD1	**7**		**(2R,3S)-7**		**(2S,3R)-7**		**8**		**(2R,3S)-8**		**(2S,3R)-8**	
	WT	L/V	WT	L/V	WT	L/V	WT	L/V	WT	L/V	WT	L/V
pIC_{50}	4.9	6.2	5.1	6.8	4.2	5.6	4.3	5.7	4.1	5.9	4.6	5.0
SE	0.07	0.04	0.03	0.07	0.07	0.05	0.08	0.06	0.11	0.06	0.18	0.12
ΔpIC_{50}	1.4		1.7		1.4		1.4		1.8		0.4	

Table S7 – AlphaLISA Testing of Separated Enantiomers

Results of titrations of 7 and 8 enantiomers and racemic mixtures against WT and L/V BRD4 BD1 in competitive AlphaLISA assay. Values refer to total compound concentration.

BRD4 BD1	**(2R,3S)-7**		**(2S,3R)-7**		**7**
	WT	L/V	WT	L/V	L/V
Kd (nM)	58000 ±4000	170 ±20	>60 000	>60 000	500 ±50
ΔH (kcal/mol)	-13.3 ±0.5	-7.9 ±0.1			-4.2 ±0.0
N	1 (FIX)	1.06 ±0.01			1.68 ±0.01

BRD4 BD1	**(2R,3S)-8**		**(2S,3R)-8**		**8**
	WT	L/V	WT	L/V	L/V
Kd (nM)	>60 000	440 ±50	>60 000	>60 000	840 ±170
ΔH (kcal/mol)		-6.5 ±0.1			-3.5 ±0.1
N		0.85 ±0.01			1.42 ±0.02

Table S8 – ITC Testing of Separated Enantiomers

7 and 8 enantiomers and racemic mixtures titrated against WT and L/V BRD4 BD1 in ITC. Values refer to total compound concentration. For some weak interactions N was fixed to 1.0.

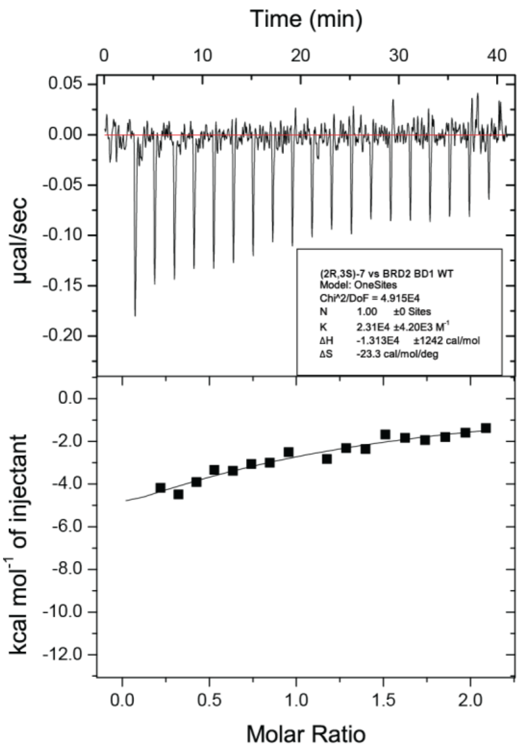
Time (min)
µcal/sec
(2R,3S)-7 vs BRD2 BD1 WT
Model: OneSites
Chi^2/DoF = 4.915E4
N 1.00 ±0 Sites
K 2.31E4 ±4.20E3 M^-1
ΔH -1.313E4 ±1242 cal/mol
ΔS -23.3 cal/mol/deg
kcal mol^-1 of injectant
Molar Ratio

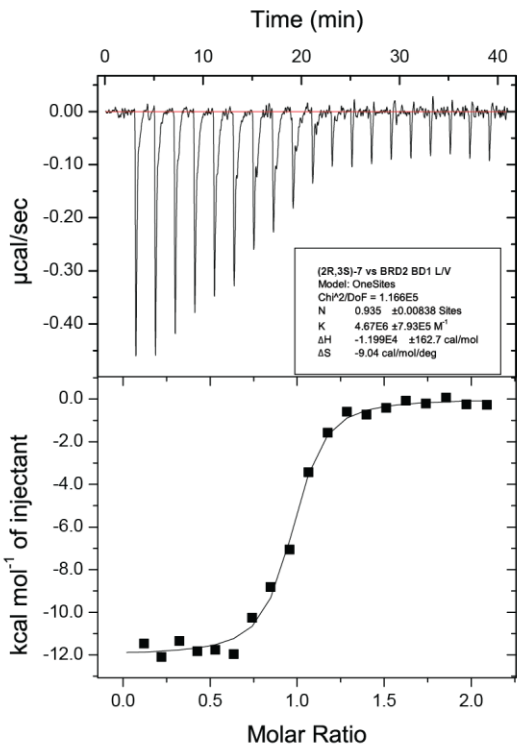
Time (min)
µcal/sec
(2R,3S)-7 vs BRD2 BD1 L/V
Model: OneSites
Chi^2/DoF = 1.166E5
N 0.935 ±0.00838 Sites
K 4.67E6 ±7.93E5 M^-1
ΔH -1.199E4 ±162.7 cal/mol
ΔS -9.04 cal/mol/deg
kcal mol^-1 of injectant
Molar Ratio

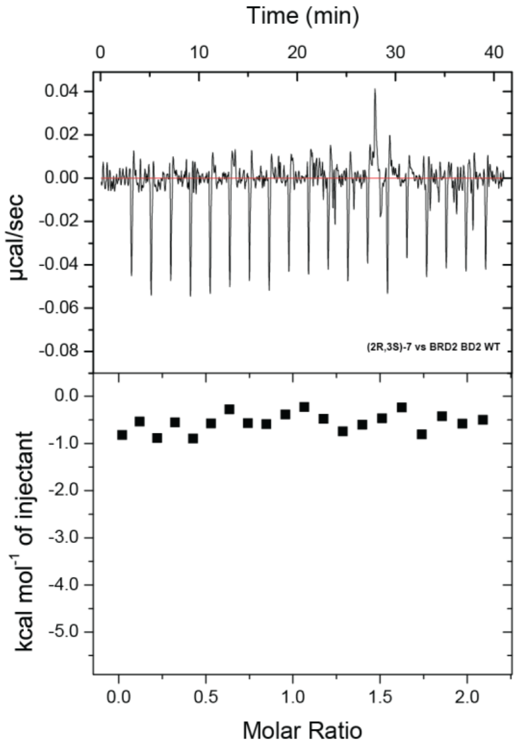
Time (min)
µcal/sec
(2R,3S)-7 vs BRD2 BD2 WT
kcal mol^-1 of injectant
Molar Ratio

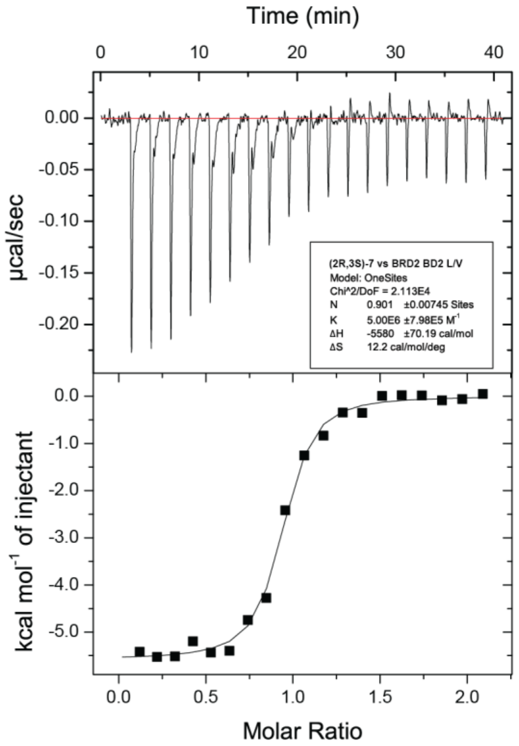
Time (min)
µcal/sec
(2R,3S)-7 vs BRD2 BD2 L/V
Model: OneSites
Chi^2/DoF = 2.113E4
N 0.901 ±0.00745 Sites
K 5.00E6 ±7.98E5 M^-1
ΔH -5580 ±70.19 cal/mol
ΔS 12.2 cal/mol/deg
kcal mol^-1 of injectant
Molar Ratio

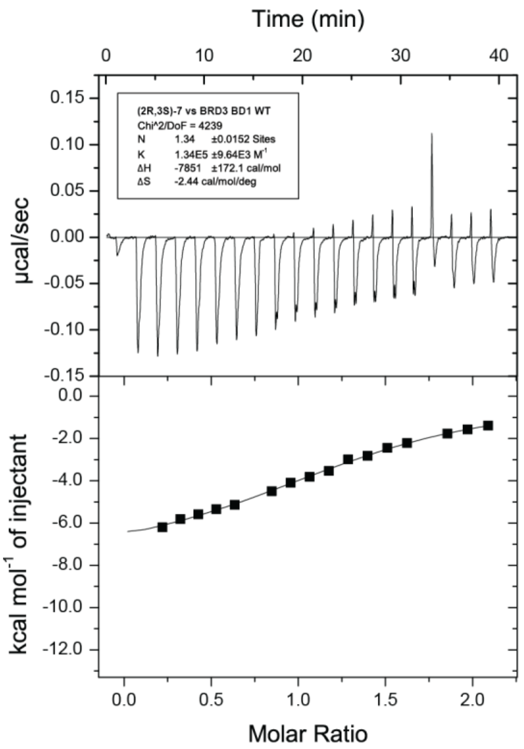
Time (min)
(2R,3S)-7 vs BRD3 BD1 WT
Chi^2/DoF = 4239
N 1.34 ±0.0152 Sites
K 1.34E5 ±9.64E3 M-1
ΔH -7851 ±172.1 cal/mol
ΔS -2.44 cal/mol/deg
µcal/sec
kcal mol-1 of injectant
Molar Ratio

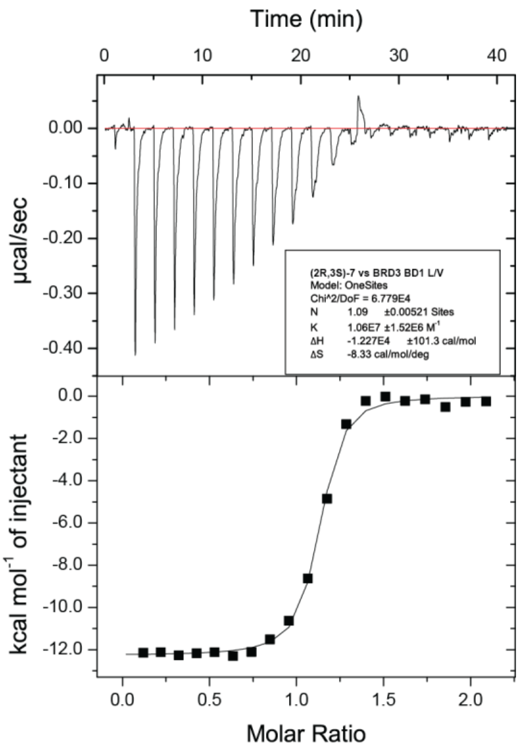
Time (min)
(2R,3S)-7 vs BRD3 BD1 L/V
Model: OneSites
Chi^2/DoF = 6.779E4
N 1.09 ±0.00521 Sites
K 1.06E7 ±1.52E6 M-1
ΔH -1.227E4 ±101.3 cal/mol
ΔS -8.33 cal/mol/deg
µcal/sec
kcal mol-1 of injectant
Molar Ratio

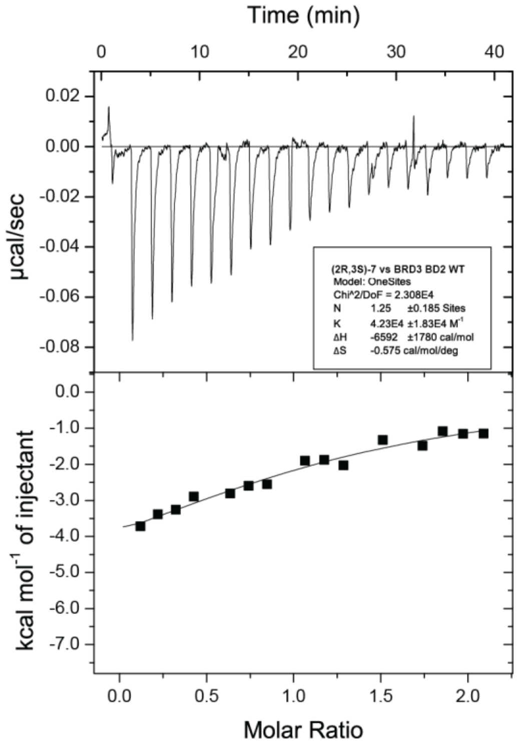
Time (min)
(2R,3S)-7 vs BRD3 BD2 WT
Model: OneSites
Chi^2/DoF = 2.308E4
N 1.25 ±0.185 Sites
K 4.23E4 ±1.83E4 M-1
ΔH -6592 ±1780 cal/mol
ΔS -0.575 cal/mol/deg
µcal/sec
kcal mol-1 of injectant
Molar Ratio

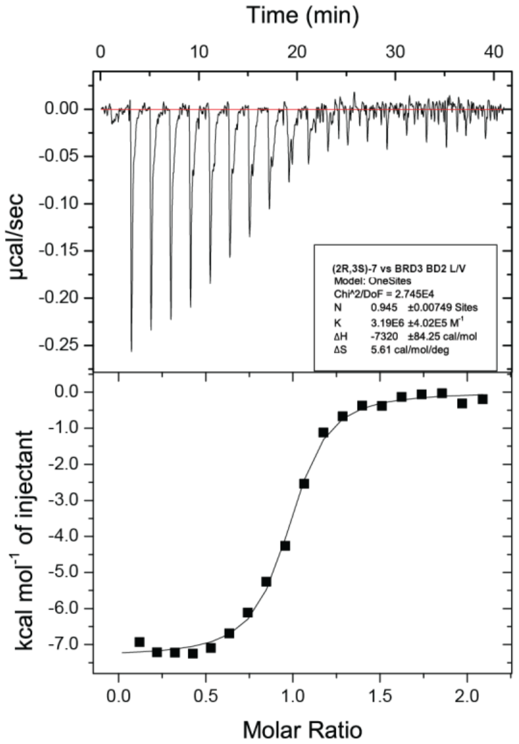
Time (min)
(2R,3S)-7 vs BRD3 BD2 L/V
Model: OneSites
Chi^2/DoF = 2.745E4
N 0.945 ±0.00749 Sites
K 3.19E6 ±4.02E5 M-1
ΔH -7320 ±84.25 cal/mol
ΔS 5.61 cal/mol/deg
µcal/sec
kcal mol-1 of injectant
Molar Ratio

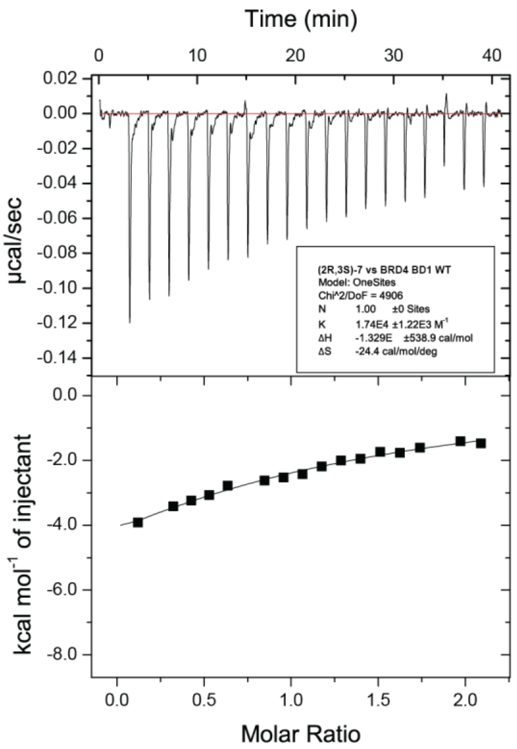
Time (min)
0 10 20 30 40
µcal/sec
0.02 0.00 -0.02 -0.04 -0.06 -0.08 -0.10 -0.12 -0.14
(2R,3S)-7 vs BRD4 BD1 WT
Model: OneSites
Chi^2/DoF = 4906
N 1.00 ±0 Sites
K 1.74E4 ±1.22E3 M-1
ΔH -1.329E ±538.9 cal/mol
ΔS -24.4 cal/mol/deg
kcal mol-1 of injectant
0.0 -2.0 -4.0 -6.0 -8.0
0.0 0.5 1.0 1.5 2.0
Molar Ratio

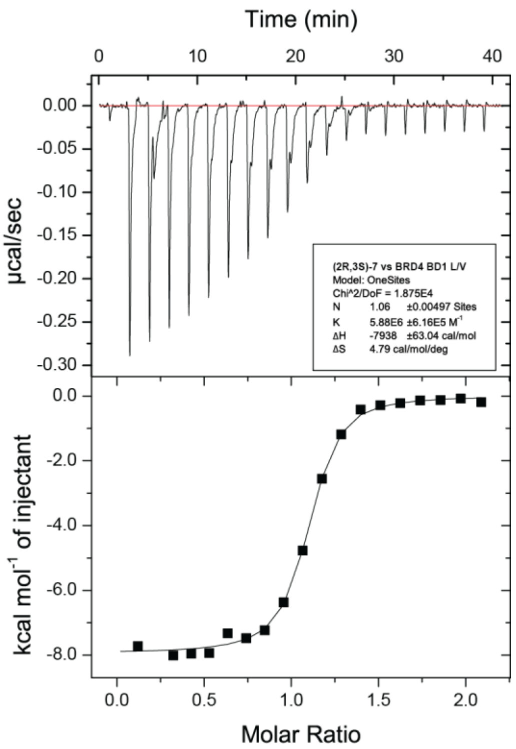
Time (min)
0 10 20 30 40
µcal/sec
0.00 -0.05 -0.10 -0.15 -0.20 -0.25 -0.30
(2R,3S)-7 vs BRD4 BD1 L/V
Model: OneSites
Chi^2/DoF = 1.875E4
N 1.06 ±0.00497 Sites
K 5.88E6 ±6.16E5 M-1
ΔH -7938 ±63.04 cal/mol
ΔS 4.79 cal/mol/deg
kcal mol-1 of injectant
0.0 -2.0 -4.0 -6.0 -8.0
0.0 0.5 1.0 1.5 2.0
Molar Ratio

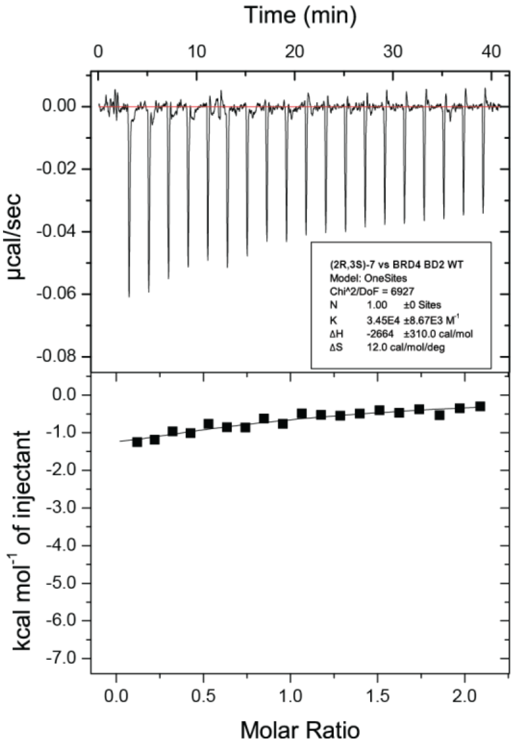
Time (min)
0 10 20 30 40
µcal/sec
0.00 -0.02 -0.04 -0.06 -0.08
(2R,3S)-7 vs BRD4 BD2 WT
Model: OneSites
Chi^2/DoF = 6927
N 1.00 ±0 Sites
K 3.45E4 ±8.67E3 M-1
ΔH -2664 ±310.0 cal/mol
ΔS 12.0 cal/mol/deg
kcal mol-1 of injectant
0.0 -1.0 -2.0 -3.0 -4.0 -5.0 -6.0 -7.0
0.0 0.5 1.0 1.5 2.0
Molar Ratio

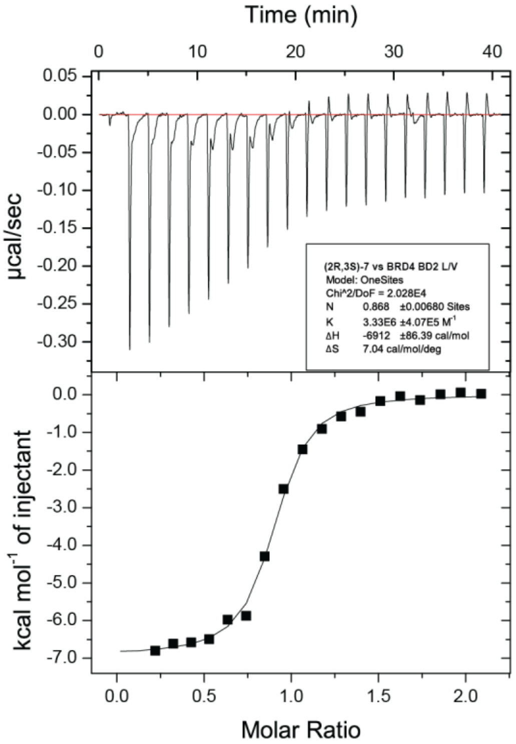
Time (min)
0 10 20 30 40
µcal/sec
0.05 0.00 -0.05 -0.10 -0.15 -0.20 -0.25 -0.30
(2R,3S)-7 vs BRD4 BD2 L/V
Model: OneSites
Chi^2/DoF = 2.028E4
N 0.868 ±0.00680 Sites
K 3.33E6 ±4.07E5 M-1
ΔH -6912 ±86.39 cal/mol
ΔS 7.04 cal/mol/deg
kcal mol-1 of injectant
0.0 -1.0 -2.0 -3.0 -4.0 -5.0 -6.0 -7.0
0.0 0.5 1.0 1.5 2.0
Molar Ratio

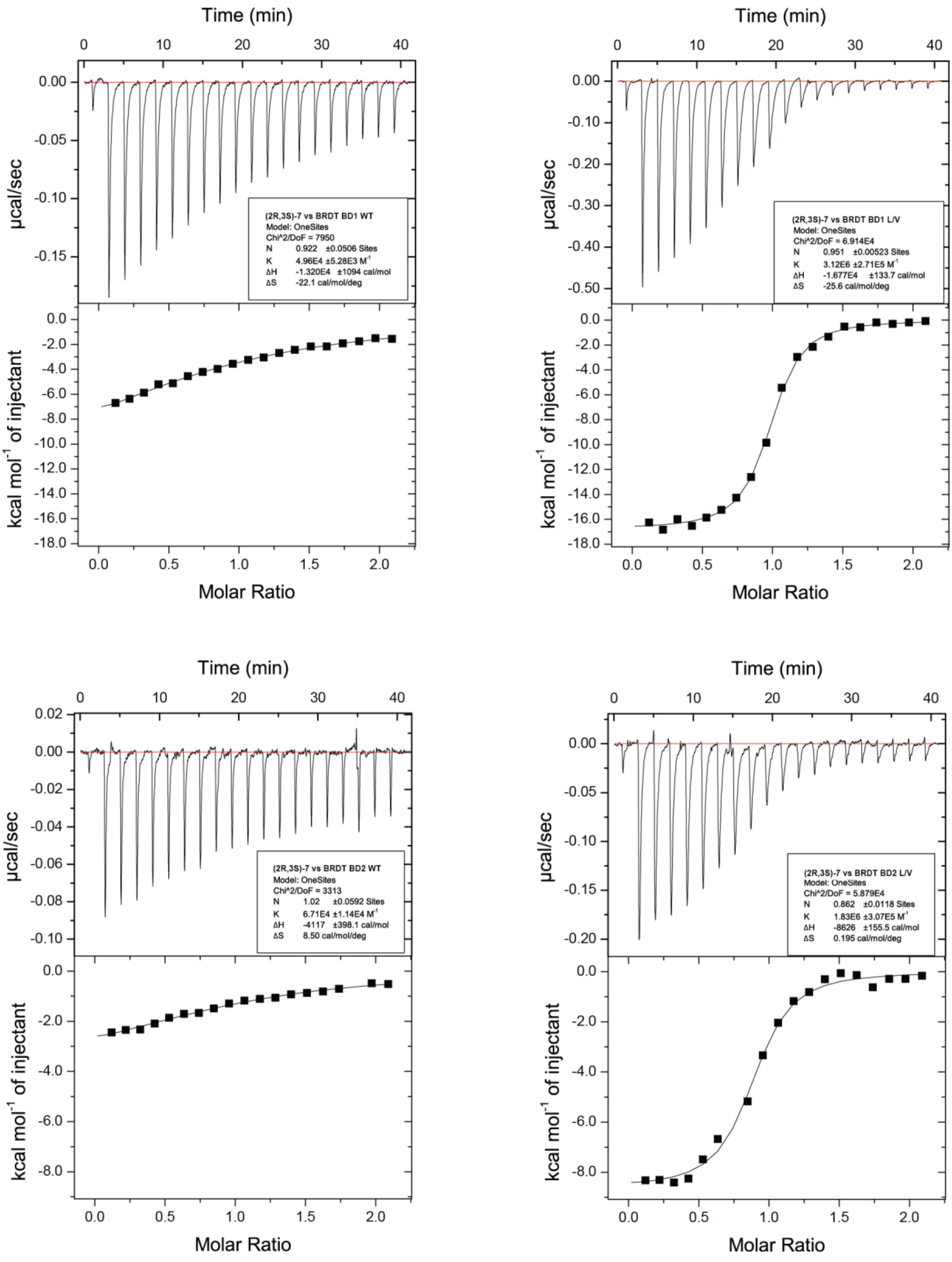

Figure S11. 9-ME-1 ITC Profile

Titrations of 9-ME-1 (7a) into WT and L/V BET bromodomains. For some weak interactions N value fixed to 1.0.

A

1 vs BRD4 BD1 WT	
ITC Kd	150 nM
AlphaLISA IC_{50}	140 nM
BROMOscan Kd	15 nM

B

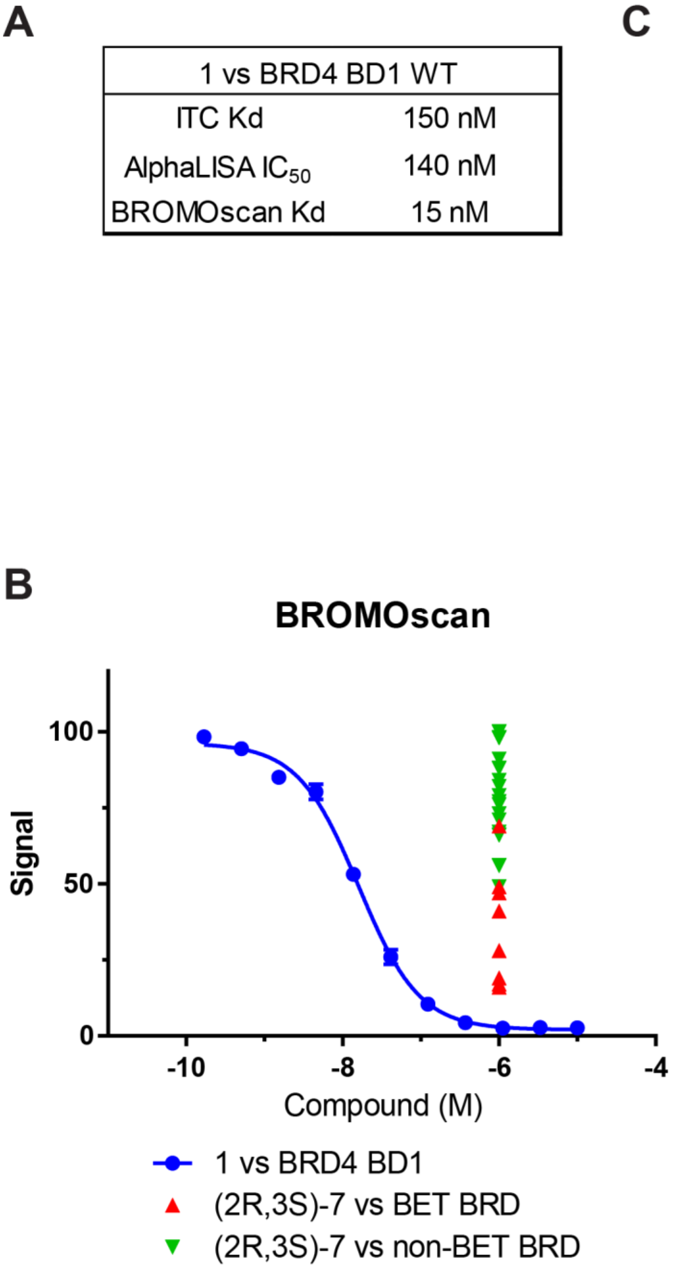

C

Bromodomain	Signal Remaining (%)
BRD2 BD1	49
BRD2 BD2	16
BRD3 BD1	19
BRD3 BD2	17
BRD4 BD1	47
BRD4 BD2	28
BRDT BD1	69
BRDT BD2	41
SMARCA4	49
SMARCA2	56
WDR9 BD2	56
BRD9	66
CREBBP	66
EP300	67
CECR2	71
PBRM1 BD2	73
BRPF1	76
PCAF	77
FALZ	79
BAZ2B	82
BRPF3	84
BRD1	88
GCN5L2	88
TAF1 BD2	91
ATAD2B	98
BAZ2A	98
BRD7	98
PBRM1 BD5	98
ATAD2A	100
TAF1L BD2	100
TRIM24	100
TRIM33	100

Figure S12. BROMOscan Screen.

A) Binding constants for **1** binding to BRD4 BD1 WT. B) BROMOscan titration of **1** against BRD4 BD1 WT, overlaid with results of C. C) BROMOscan signal generated by panel of human bromodomains following treatment with 1 µm **(2R, 3S)-7**. **1** tested through bromoKdELECT (DiscoveRX) and **(2R, 3S)-7** tested in bromoMAX (DiscoveRX). https://www.discoverx.com/services/drug-discovery-development-services/epigenetic-profiling/bromoscan

Kinase	Remaining activity (%)	S.D.
MST2	118	14
IRAK4	114	10
HER4	114	9
SmMLCK	112	1
NEK6	109	12
TAK1	108	1
CHK2	107	8
PIM1	106	10
PAK4	104	11
AMPK (hum)	104	3
SGK1	103	5
RIPK2	103	20
TTK	102	6
p38a MAPK	101	6
CAMKKb	101	1
PRK2	100	1
MLK3	100	4
HIPK2	99	1
RSK1	99	2
TrkA	98	0
SYK	98	13
TBK1	98	2
EF2K	97	6
CK2	97	0
PDK1	97	5
PKCa	96	5
BTK	96	4
JAK3	95	7
GSK3b	95	10
PKBa	95	9
MSK1	94	1
CAMK1	94	5
MARK3	94	1
Aurora B	94	16
IGF-1R	93	2
CK1δ	93	11
S6K1	93	4
PKA	92	12
LKB1	91	9
JNK1	89	16
VEG-FR	89	13
MKK1	89	2
PLK1	89	3
ROCK 2	88	3
EPH-A2	86	15
Lck	86	4
Src	82	4
DYRK1A	80	8
SRPK1	79	6
PKD1	78	4

Table S9. 9-ME-1 Kinase Screen

Activity of panel of human kinases following treatment with 1 μM **(2R, 3S)-7**. Dundee MRC-PPU Express Screen (http://www.kinase-screen.mrc.ac.uk/services/express-screen)

Receptor	Reference Compound	Mean % of control specific binding (±SD)
Y1 (h)	NPY	122 (16)
mu (MOP) (h)	DAMGO	119 (3)
H2 (h)	cimetidine	117 (28)
D1 (h)	SCH 23390	116 (6)
5-HT6 (h)	serotonin	112 (4)
Y2 (h)	NPY	110 (13)
GABA (non-selective)	GABA	110 (1)
5-HT2B (h)	(±)DOI	109 (0)
5-HT5a (h)	serotonin	109 (6)
sst (non-selective)	somatostatin-14	108 (6)
Ca2+ channel (L, verapamil site)	D 600	108 (6)
H1 (h)	pyrilamine	108 (3)
NTS1 (NT1) (h)	neurotensin	106 (3)
5-HT7 (h)	serotonin	106 (6)
beta 2 (h)	ICI 118551	105 (4)
Na+ channel (site 2)	veratridine	105 (6)
BZD (central)	diazepam	105 (10)
M2 (h)	methoctramine	105 (0)
M1 (h)	pirenzepine	104 (13)
5-HT1A (h)	8-OH-DPAT	104 (1)
SKCa channel	apamin	103 (10)
CCR1 (h)	MIP-1alpha	102 (4)
V1a (h)	[d(CH2)51,Tyr(Me)2]-AVP	102 (3)
NK2 (h)	[Nleu10]-NKA (4-10)	101 (4)
M3 (h)	4-DAMP	101 (2)
A1 (h)	DPCPX	100 (0)
delta (DOP) (h)	DPDPE	100 (4)
5-HT2A (h)	ketanserin	100 (3)
KV channel	alpha -dendrotoxin	100 (0)
NK3 (h)	SB 222200	99 (7)
A2A (h)	NECA	99 (5)
AT1 (h)	saralasin	99 (0)
alpha 1 (non-selective)	prazosin	99 (12)
B2 (h)	NPC 567	98 (1)
5-HT1B	serotonin	98 (9)
Cl- channel (GABA-gated)	picrotoxinin	98 (4)
VPAC1 (VIP1) (h)	VIP	98 (13)
alpha 2 (non-selective)	yohimbine	98 (0)
CXCR2 (IL-8B) (h)	IL-8	96 (10)
5-HT3 (h)	MDL 72222	96 (1)
kappa (KOP)	U 50488	96 (9)
GAL2 (h)	galanin	96 (1)
5-HT transporter (h)	imipramine	96 (2)
D2S (h)	(+)butaclamol	95 (3)
beta 1 (h)	atenolol	95 (8)
NOP (ORL1) (h)	nociceptin	94 (4)
EP4 (h)	PGE2	93 (2)
A3 (h)	IB-MECA	91 (7)
CB1 (h)	CP 55940	90 (4)
CCK1 (CCKA) (h)	CCK-8s	90 (15)
MC4 (h)	NDP-alpha -MSH	89 (15)
norepinephrine transporter (h)	protriptyline	88 (4)
ETA (h)	endothelin-1	87 (3)
dopamine transporter (h)	BTCP	84 (1)
MT1 (ML1A) (h)	melatonin	23 (2)

Table S10. Receptor & Ion-Channel Screening

Activity of panel of receptors and ion channels following treatment with 1 µM **(2R, 3S)-7**. CEREP ExpresSProfile panel http://www.cerep.fr/cerep/utilisateurs/pages/downloads/Documents/Marketing/Pharmacology%20&%20ADME/Standard%20profiles/ExpresSProfile_2014v2LD.pdf

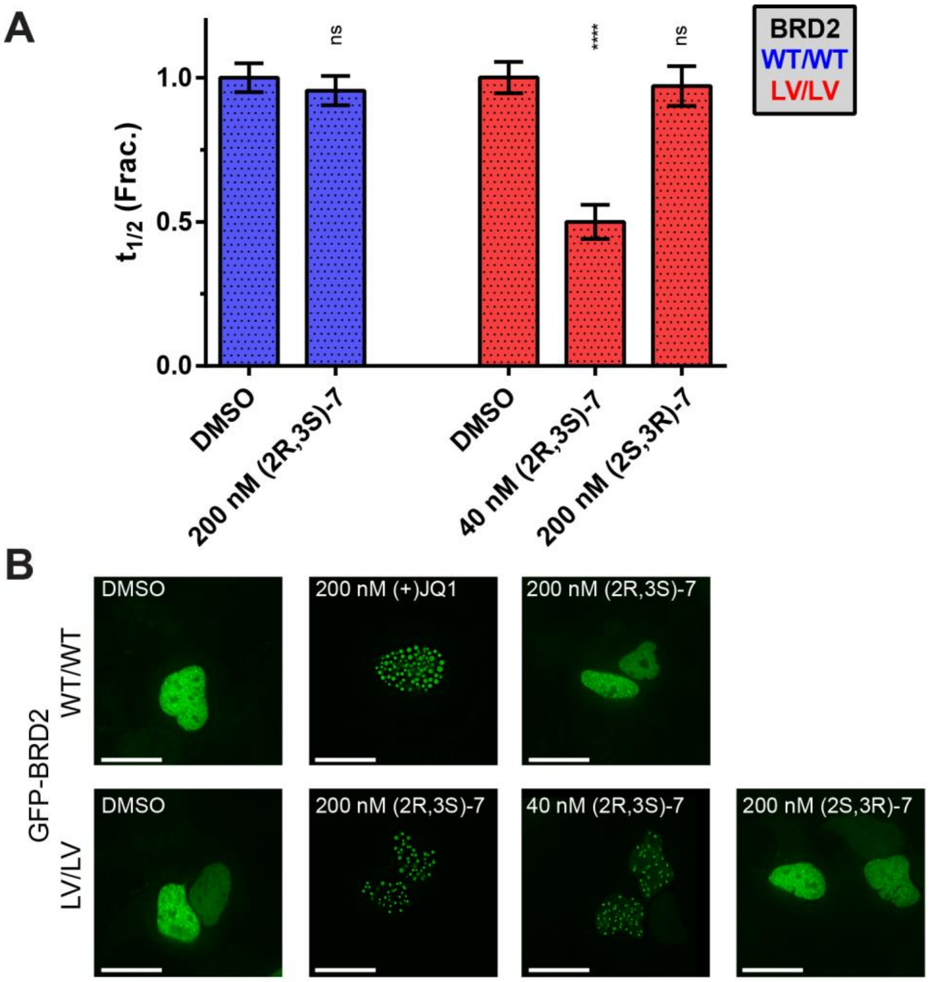

Figure S13 – GFP-BRD2 Aggregates upon Inhibition

A) Recovery times of GFP-labelled BRD2 constructs in FRAP assay, following 0.5s laser bleach event, at 2 µM SAHA and 0.03% DMSO. Each bar is mean and SE of ~30 U2OS cells. BRD2 LV/LV + 40 nM **(2R, 3S)-7** showed some aggregation but recovery time could still be determined. Statistical significance determined with two-tailed *t* tests: ns P>0.05; * P ≤ 0.05, ** P≤0.01, *** P≤0.001, **** P<0.0001.

B) Nuclei of transfected U2OS cells, treated with 2 µM SAHA, 0.03% DMSO and test compound. Scale bar = 20 µm.

X-Ray Crystallography: Data Collection & Refinement Statistics

Structure	Apo	3	4	5	7	8
**PDB code**	**5O38**	**5O39**	**5O3A**	**5O3B**	**503C**	**5O3D**
**Data Collection**						
Space group	P 21 21 2	P 21 21 2	I 41 2 2	I 222	P 2 21 21	P 21 21 2
Cell dimensions						
a, *b*, *c* (Å)	52.420, 71.409, 31.969	52.406 72.129 31.990	126.799, 126.799, 43.194	88.075 101.893 123.534	31.852 52.082 71.789	52.407 71.83 32.003
α, β, γ (°)	90, 90, 90	90, 90, 90	90, 90, 90	90, 90, 90	90, 90, 90	90, 90, 90
Resolution (Å)	19.50 - 1.20 (1.243 - 1.20)	21.77-1.74 (1.802-1.74)	40.10 - 2.40 (2.486 - 2.40)	44.04 - 1.95 (2.02 - 1.95)	31.85 - 1.60 (1.657 - 1.60)	32.00 - 1.60 (1.657 - 1.60)
Rmerge	0.05918 (0.06509)	0.04883 (0.05565)	0.09961 (0.3312)	0.2827 (0.9697)	0.1111 (0.5627)	0.05236 (0.07519)
I/sigma(*I*)	17.14 (14.31)	43.16 (37.50)	17.11 (5.14)	10.53 (2.26)	7.67 (2.04)	32.70 (18.62)
$CC_{1/2}$	0.997 (0.998)	1 (0.999)	0.999 (0.984)	0.984 (0.535)	0.992 (0.755)	0.999 (0.994)
Completeness (%)	98.26 (93.37)	97.12 (79.28)	99.18 (100.00)	99.92 (99.98)	99.51 (99.56)	98.83 (94.76)
Redundancy	5.7 (5.7)	17.1 (15.3)	15.3 (14.6)	8.6 (7.8)	4.3 (4.2)	10.3 (7.2)
**Refinement**						
Resolution (Å)	19.50 - 1.20 (1.243 - 1.20)	21.77-1.74 (1.802-1.74)	40.10 - 2.40 (2.486 - 2.40)	44.04 - 1.95 (2.02 - 1.95)	31.85 - 1.60 (1.657 - 1.60)	32 .00 - 1.60 (1.657 - 1.60)
No. reflections	122513 (5072)	123746 (4452)	109065 (10146)	349867 (31395)	69717 (6561)	168336 (11018)
Unique no. of reflections	37657 (894)	12633 (291)	7114 (696)	40791 (4007)	16285 (1575)	16364 (1536)
Rwork	0.1178 (0.1068)	0.1443 (0.1466)	0.2055 (0.3278)	0.1972 (0.2710)	0.1615 (0.2197)	0.1473 (0.1294)
Rfree	0.1413 (0.1365)	0.1897 (0.2237)	0.2547 (0.3512)	0.2442 (0.3180)	0.1945 (0.2670)	0.1702 (0.1561)
No. atoms						
Protein	991	922	893	3613	918	948
Ligand/ion	31	40	31	128	40	41
Water	237	203	57	559	212	219
B factors						
Average	13.3	12.9	43.2	19.3	16.7	11.80
Protein	10.1	10.4	43.3	18.4	14.3	8.50
Ligand/ion	16.9	11.0	37.9	15.4	15.8	18.10
Water	26.0	24.9	44.4	25.9	27.6	24.80
R.m.s. deviations						
Bond lengths (Å)	0.005	0.007	0.011	0.009	0.007	0.009
Bond angles (°)	1.03	1.05	1.19	1.04	1.08	1.06

Structure<br>PDB Code<br>Data Collection	16<br>5O3E	17<br>5O3F	18<br>5O3G	21<br>5O3H	24<br>5O3I
Space group	P 21 21 2	P 21 21 2	P 21 21 2	P 21 21 2	P 21 21 2
Cell dimensions					
a, *b*, *c* (Å)	52.177, 71.95, 31.917	51.965 71.548 31.704	73.74, 52.66, 63.12	52.143, 71.837, 31.97	53.12, 72.829, 32.359
α, β, γ (°)	90, 90, 90	90, 90, 90	90, 90, 90	90, 90, 90	90, 90, 90
Resolution (Å)	35.98 - 1.40 (1.45 - 1.40)	42.05 - 1.75 (1.813 - 1.75)	25.41 - 1.85 (1.916 - 1.85)	35.92 - 1.40 (1.45 - 1.40)	14.31 - 1.20 (1.243 - 1.20)
Rmerge	0.1005 (0.2228)	0.1116 (0.1436)	0.07581 (0.3878)	0.06188 (0.1025)	0.1049 (0.5239)
I/sigma(*I*)	15.07 (4.57)	7.72 (6.60)	9.64 (3.00)	21.35 (7.92)	9.10 (3.25)
$CC_{1/2}$	0.998 (0.955)	0.992 (0.99)	0.997 (0.776)	0.999 (0.982)	0.994 (0.781)
Completeness (%)	99.77 (97.92)	99.66 (99.92)	95.19 (95.67)	98.72 (93.14)	99.19 (99.29)
Redundancy	9.6 (4.8)	3.9 (4.0)	3.9 (3.9)	7.5 (3.7)	5.8 (5.8)
**Refinement**					
Resolution (Å)	35.98 - 1.40 (1.45 - 1.40)	42.05 - 1.75 (1.813 - 1.75)	25.41 - 1.85 (1.916 - 1.85)	35.92 - 1.40 (1.45 - 1.40)	14.31 - 1.20 (1.243 - 1.20)
No. reflections	232876 (11299)	27589 (1075)	79692 (7845)	180745 (8305)	229769 (22505)
Unique no. of reflections	24337 (2352)	12439 (269)	20578 (2034)	24057 (2228)	39710 (3911)
Rwork	0.1315 (0.1401)	0.1721 (0.2604)	0.1888 (0.2460)	0.1477 (0.1678)	0.1557 (0.2044)
Rfree	0.1653 (0.1794)	0.2018 (0.2902)	0.2356 (0.2788)	0.1636 (0.1633)	0.1784 (0.2326)
No. atoms					
Protein	931	909	1818	932	939
Ligand/ion	41	42	66	41	45
Water	218	185	196	229	218
B factors					
Average	12.7	14.3	26.5	14.3	14.2
Protein	9.7	12.2	25.9	11.1	11.2
Ligand/ion	11.5	14.8	26.3	13.6	18.1
Water	26.0	24.7	32.0	27.5	26.2
R.m.s. deviations					
Bond lengths (Å)	0.006	0.006	0.014	0.011	0.019
Bond angles (°)	1.68	1.01	1.52	1.15	1.75

Materials & Methods

1 – Cloning & Mutagenesis

Plasmids pNIC28-Bsa4 KanR containing the 8 single BET bromodomain constructs BRD2 BD1 (protein start and stop positions K71-K176), BRD2 BD2 G344-D455, BRD3 BD1 P24-E144, BRD3 BD2 G306- P416, BRD4 BD1 N44-E168, BRD4 BD2 K333-E460, BRDT BD1 N21-E137 and BRDT BD2 S257-M361 were provided by the Oxford Structural Genomics Consortium (SGC) for expression with an N-terminal His6-tag and a TEV protease cleavage site (UniProt accession number, BRD2: P25440; BRD3: Q15059; BRD4: O60885). For tandem construct of BRD2, cDNA encoding both bromodomains and the linker region (G73-D455) was cloned from full-length cDNA clone purchased from DNASU Plasmid Repository at the Arizona State University into a pCri (11b) vector (based on pET15b, AmpR) for expression with an N-terminal His6-tag, a Small Ubiquitin-like Modifier (SUMO) tag, and a SUMO protease (SENP1) cleavage site. Plasmid pcDNA5-FRT/TO-GFP and cDNA clones for full length BRD2, BRD3 and BRD4 were gifts from Dr Mark Peggie from University of Dundee. Full-length cDNA of human BRD2 (M1-G801), BRD3 (M1-E726) and BRD4 (M1-F1362) were cloned into pcDNA5-FRT/TO-GFP to generate constructs for expressing corresponding protein in mammalian cell line. GFP-BRDT (pcDNA6.2/N-EmGFP-DEST)[1] was a gift from Kyle Miller (Addgene plasmid # 65381). NF-κB-RE/luc2P luciferase reporter plasmid consists of one luc2P gene, controlled by 5 copies of an NF-κB response element, cloned from a pGL4.32 vector into a pBABE vector. Luciferase reporter plasmid provided by Dr. Mark Peggie (Division of Signal Transduction Therapy (DSTT), MRC-PPU, University of Dundee).

Single point mutations were introduced using QuickChange II Site directed Mutagenesis Kit (Agilent). Primers were designed following the recommendations from manufacturer. The polymerase chain reaction (PCR) was performed on a 2720 Thermal Cycler (Applied Biosystems®). Upon digestion of the parental DNA strands by DpnI restriction enzyme, the PCR product was transformed into competent E. coli DH5α cells and grown on lysogeny broth (LB) agar plates containing corresponding selection antibiotics at 37°C for 16h. Single colonies were then picked from the agar plates and grown for 12h in 10 mL of LB medium containing selection antibiotics. Extracted and purified plasmid DNA were then sequenced to confirm the presence of the desired mutation.

2 – Protein Expression & Purification

BL21 E. coli cells were transformed with bromodomain-expressing pNIC plasmids using heat shock. Cell cultures were grown at 37°C and 200 rpm in LB media with 50 µg/ml kanamycin. A starter culture was incubated until saturation, then diluted 100-fold in fresh media and grown until reaching an optical density of 2.0 (OD_{600}). The temperature of the culture was decreased to 18°C, and protein expression was induced overnight with 0.4 mM IPTG. Cells were harvested the next day by centrifugation (4000 rpm for 20 minutes at 4°C, JS-4.2 rotor on a Beckman J6-MC centrifuge) and stored as pellets at -20°C.

Cell pellets were resuspended in lysis buffer (50 mM HEPES, 500 mM NaCl, 10 mM imidazole & 2 mM β-mercaptoethanol pH7.5) and treated with one complete protease inhibitor (Roche) tablet. Cells were lysed using a Stanstead pressure cell homogenizer and the lysate centrifuged at 20 000 rpm for 1 hour at 4°C (JA-25.50 rotor in Beckman Avanti J-25 centrifuge) and the supernatant transferred and passed through a 0.45 um filter.

The lysate was purified using metal ion affinity chromatography, with a His Trap 5ml Ni sepharose column (GE Healthcare) on an AKTApure™ system (GE Healthcare). The column was washed with 30 ml of lysis buffer, and the protein was eluted using 30 ml of elution buffer (50 mM HEPES, 500 mM NaCl, 250 mM imidazole and 2 mM β-mercaptoethanol at pH 7.5). The Ni column elution was concentrated to 2 ml using a Vivaspin 20, 10 000 MWCO (Sartorius) before further purification using size exclusion chromatography. Concentrated Ni column elution was passed through a Superdex 75 16/60 Hiload column (GE healthcare) on an AKTApure™ system, using gel filtration buffer (20 mM HEPES, 150 mM NaCl and 1 mM DTT at pH 7.5). Desired fractions were pooled, concentrated and aliquoted before being flash frozen and stored at -80°C.

3 – Differential Scanning Fluorimetry (DSF) / Thermal Shift

DSF experiments were performed on a BioRad CFXconnect machine, using clear 96-well plates. Protein constructs were tested at 6 µM, with 2.5X SYPRO orange (Invitrogen), in 40 µl of buffer (10 mM HEPES, 100 mM NaCl, pH7.5). Samples underwent a heat cycle from 20°C to 95°C, heated at a rate of 1°C every minute. Plates were

read at 1 minute intervals. Each sample was tested in triplicate. Data was analyzed as recommended by Niesen et al[2], using GraphPad Prism 6 and the SGC's DSF Analysis 3.02 spreadsheet.

4 – X-ray Crystallography

Purified BRD2 BD2 L/V protein at 8 mg/mL was mixed with excess amount of a bumped compound (2-4 mM) to form a protein-compound complex. Drops of the complex were mixed 2:1 with precipitant solution in sitting-drop vapour diffusion format. Crystallization condition of protein complexes varies with different bumped compounds, the condition ranges from 0.1 M Tris pH 7.5 - 9.0, 45 - 60 % pentaerythiol propoxylate (5/4 PO/OH), with or without 0.2 M imidazole as additive. Crystals appeared within hours and were fully grown after 2-3 d. Diffraction data were either collected at in-house Rigaku M007HF X-ray generator equipped with Varimax Cu-VHF optics using a Saturn 944HG+ CCD detector at a wavelength of 1.5418 Å or Diamond Light Source beamline I04-1 using a Pilatus 6M-F detector at a wavelength of 0.92819 Å at temperature 100K.

Indexing and integration of reflections was either performed using XDS with the XDSGUI interface[3] or MOSFLM[4], and scaling and merging with AIMLESS[5] in CCP4i[6]. To solve the phase problem the molecular replacement method was used with the programs MOLREP[7] and Phaser[8] using search models derived from the coordinates of BRD2 BD2 WT (PDB entry 2DVV). The initial model was refined iteratively using PHENIX[9, 10] and COOT[11]. Ligand structures and restraints were generated using eLBOW[12], REEL[13] and PRODRG[14]. The MOLPROBITY server[15] was used to validate the geometry and steric clashes in the structures. The structure models have been deposited in the protein data bank (PDB) and data collection and refinement statistics are presented in supplementary information. All figures were generated using PyMOL[16].

5 – Bio-Layer Interferometry (BLI)

All BLI experiments were carried out on a OctetRed 384 instrument (ForteBio), at 25°C and in 20 mM HEPES, 150 mM NaCl pH7.5 buffer. A histone peptide library, possessing both single and multiple lysine acetylation marks, was obtained from Alta Bioscience Ltd. Peptides were 20 residues long and were biotinylated on the C-terminal (with aminohexanoic linker). Streptavidin-coated BLI tips (ForteBio) were loaded in 100 µl 5 µM peptide (or 10 µg/ml biocytin) over 10 minutes. For the assay bromodomain constructs were kept at 20 µM in 100 µl buffer in black 384-well plates, agitated at 1000rpm. Each assay involved exposing peptide-loaded BLI tips to buffer for 120s to determine baseline signal, 240s in protein to measure association and finally 120s in buffer to measure dissociation. Raw data was then analyzed using the ForteBio software, to account for background signal and non-specific binding.

6 – Isothermal Titration Calorimetry (ITC)

ITC titrations were performed on an ITC_{200} instrument (MicroCal™, GE Healthcare). Proteins, peptides and compounds were all dissolved in ITC buffer (20 mM HEPES, 100 mM NaCl, pH7.5), with protein samples buffer-exchanged through dialysis using D-tubes (Millipore). Each ITC titrations consisted of 20 titrations: 1 initial injection of 0.4 µl over 0.8s, followed by 19 injections of 2 µl over 4s, at 2 minute intervals. Data was analyzed using the Microcal LLC ITC200 Origin data analysis software, using a single binding site model, to determine binding values such as K_d and enthalpy of binding.

Peptide titrations were carried out at 15°C, with 2 mM of acetylated histone peptide titrated against 50-150 µM bromodomain. Ligand titrations were performed at 30°C, with 250 µM active ligand titrated against 25 µM bromodomains, with a final DMSO concentration of 2.5%. Tandem constructs were carried out at 30°C and with 1.5% DMSO, with 150 µM compound titrated into 15 µM protein.

7 – AlphaLISA

In all competitive AlphaLISA assays ligand was titrated against 100 nM His-tagged bromodomain and 5 nM biotinylated JQ1 (bio-JQ1), in AlphaLISA buffer: 50 mM HEPES, 100 mM NaCl, 0.1% BSA, 0.02% CHAPS, pH7.5 (final concentration). Ligands were tested over an 11-point 3-fold serial dilution, starting at 100 µM, and giving a final DMSO concentration of 1%. Binding was detected using anti-His6 antibody-conjugated AlphaLISA acceptor beads and streptavidin-coated AlphaScreen donor beads (PerkinElmer), with a final concentration of 10 µg/ml for each

bead). Titrations were prepared in light-grey 384-well Alphaplates (PerkinElmer), and read on a Pherastar FS plate reader (BMG) equipped with an AlphaLISA excitation/emission module. Data was analyzed and dose-response curves generated using GraphPad Prism 6.

Each assay well had a final volume of 25 µl. First 5 µl of 5X ligand was mixed with 10 µl of a 2.5X mix of bromodomains and Bio-JQ1 (prepared en-masse, aliquoted, flash-frozen and stored at -80°C) and incubated for 1 hour at room temperature. The assay plate was then moved to a dark room and 5 µl of 5X acceptor beads were added and incubated for 1 hour. Then (still in darkness) 5 µl of 5X donor beads were added, the plate was incubated for 1 more hour before being read. The direct titrations of Bio-JQ1 against bromodomains follows the same procedure, but in the first step of the assay 5 µl 5X Bio-JQ1 was mixed with 10 µl 5X bromodomain. Bio-JQ1 followed an 11-step 3-fold serial dilution, starting at 10 µM (final concentration).

8 – DMPK

Plasma Half-life:

50 µM test compound incubated in mouse BALB/c plasma (pre-warmed to 37°C and buffered to pH7.4 in ratio of 70:30 plasma:buffer). At 0, 30, 60, 120 and 180 minutes a 50 µl aliquot of incubation mixture were removed and mixed with 200ul acetonitrile, containing 50 ng/ml Donepezil as the internal standard, to stop the reaction. The samples were centrifuged to sediment any precipitated protein and the microplates sealed prior to UPLC-MS/MS analysis using a Quattro Premier XE (Waters Corporation). XLfit (IDBS) was used to calculate the exponential decay and hence the rate constant (K) using the ratio of the peak areas of the test compound to the internal standard at each time point. The half-life was calculated for each test compound using the formula: t1/2 = 0.693/K.

Intrinsic Clearance:

0.5 µM test compound was incubated with BAB/c female CD1 mouse liver microsomes (Xenotech LLC TM; 0.5 mg/ml 50 mM potassium phosphate buffer, pH7.4) and the reaction started with addition of excess NADPH (8 mg/ml 50 mM potassium phosphate buffer, pH7.4). At 0, 3, 6, 9, 15 and 30 minutes a 50 µl aliquot of the incubation mixture was removed and mixed with 100 µl acetonitrile to stop the reaction. Internal standard was added to all samples, which were centrifuged to sediment precipitated protein and the microplates were then sealed prior to UPLC MS/MS analysis using a Quattro Premier XE. XLfit was used to calculate the exponential decay and hence the rate constant (K), based on the ratio of the peak areas of test compound to internal standard at each time point. The rate of intrinsic clearance (CLi) was then calculated using the following formula:

CLi (ml/min/g liver) = K x V x microsomal protein yield

Where V (ml/mg protein) is the incubation volume/mg protein added, and microsomal protein yield is taken as 52.5 mg protein/g liver. 0.5 µM Verapamil used as a positive control to confirm assay performance.

PAMPA:

PAMPA was performed using a 96-well pre-coated BD Gentest™ PAMPA plate (BD Biosciences). Each well was divided into two chambers; donor and acceptor, separated by a lipid-oil-lipid tri-layer constructed in a porous filter. The effective permeability, P_e, of the compound was measured at pH 7.4. Stock solutions (5 mM) of the compound were prepared in DMSO. The compound was then further diluted to 10 µM in phosphate buffered saline at pH 7.4. The final DMSO concentration did not exceed 5% v/v. The compound dissolved in phosphate buffered saline was then added to the donor side of the membrane and phosphate buffered saline without compound was added to the acceptor side. The PAMPA plate was left at room temperature for 5 h. After which time, an aliquot (100 µl) was then removed from both acceptor and donor compartments and mixed with acetonitrile (80 µl) containing an internal standard. The samples were centrifuged (10 min, 5^0C, 3270 g) to sediment precipitated protein and sealed prior to UPLC-MS/MS analysis using a Quattro Premier XE. P_e was calculated as shown in the below equation:

$$P_e \text{ (nm/sec)} = 10^7 \quad x \quad - \ln [1 - C_A(t) / C_{equi}]) \ / \ (A * [1/V_D + 1/V_A] * t)$$

Where:

$C_A(t)$ = peak area of compound present in acceptor well at time t = 18000 sec

C_{equi} = $[C_D(t) * V_D + C_A(t) * V_A] / (V_D + V_A)$

A = filter area

VD = donor well volume

VA = acceptor well volume

t = incubation time

$C_D(t)$ = peak area of compound present in donor well at time t = 18000 sec

Recovery of compound from donor and acceptor wells was calculated and data was only accepted when recovery exceeded 70 %.

9 – Tissue Culture

Human U2OS, HEK293T and HL-60 cell lines were obtained from ATCC and MV4;11 cell line was obtained from DSMZ. U2OS and HEK293T were kept in DMEM medium (Gibco) supplemented with 10% FBS (Gibco), 1% L-glutamate (Gibco) and 100 U/ml penicillin and streptomycin. Cells were kept in an incubator at 37°C, 5% CO2. MV4-11 and HL-60 were kept in RPMI medium (Gibco) supplemented with 10% FBS, 1% L-glutamate (Gibco) and 100 U/ml penicillin and streptomycin. All cell lines were tested for mycoplasma contamination every month using MycoAlert Mycoplasma detection kit (Lonza).

10 – Fluorescence Recovery after Photobleaching (FRAP)

FRAP experiments were performed in human osteosarcoma U2OS cells transfected with pcDNA5 FRT/TO plasmids encoding wild-type and mutant GFP chimeras of BET proteins. Cells were seeded into glass-bottom, 35x10mm dishes (WillCo) at ~200 000 cells in 2 ml media per dish ~66 hours before FRAP. Cells were transfected using FuGENE HD (Promega) and 1 µg of plasmid DNA ~42 hours before FRAP. 18 hours before FRAP cells were treated with test compounds and SAHA (Sigma-Aldrich) to a final concentration of 1 µM and 2 µM, respectively, giving a final DMSO concentration of 0.03%.

FRAP experiments were carried out on a Deltavision Elite imaging system (GE Healthcare) running Resolve 3D (SoftWoRx) kept at 37°C and using a FITC filter set (488 nm excitation, 525 nm emission). Cells were imaged using a 60X objective lens (Olympus), with an exposure time of 0.05s and using 2x2 binning to give a 512x512 pixel image. Cells were excluded for FRAP experiments if they displayed aberrant morphology and signs of cell-death. Cells providing a signal of <1000 fluorescence units were excluded as they were too close to the background signal for photobleaching (requiring reduction to ~50% fluorescence) to be observed. Cells fluorescing over 3500 units were also excluded due to the risk of detector saturation.

Cells were photobleached using a quantifiable laser module (QLM), set to a wavelength of 488 nm and 100% power, for 0.5s, covering an area with a 0.5 μm^2 radius. For each cell 5 images were captured pre-bleach (over 5 seconds) and 32 post-bleach, using a CoolSNAP HQ camera (Photometrics). Post-bleach imaging was usually spread over 60s and distributed to best measure a t½ of ~2s, although this was altered in some cases. FRAP experiments were analyzed in SoftWorx (GE Healthcare) using the PK analysis function, set to analyze a bleach event with 0.5 μm^2 radius. Calculated t½ values were extracted and analyzed in Prism 6 (GraphPad). Each experiment was run twice on separate days, with ~20 cells tested on each day.

11 – WT Cell Cytotoxicity

Compound cytotoxicity was measured using the CellTiter-Glo assay (Promega). Compounds were serially diluted in a sterile, white, clear-bottom 384-well cell culture microplate (Greiner Bio-one), at 2X concentration and a volume of 25 µl. 25 µl of 2X cell suspension was then added. Both cells and compounds were diluted in RPMI medium. After 48hr incubation 25 µl of CellTiter-Glo reagent was added to each cells. Following 15 minute incubation the luminescence signal was read on a Pherastar FS. The final concentration of assay components are as follows: $3x10^5$ cells/ml, 0.05% DMSO, 5 µM and below compound. Compounds were tested in triplicate, against the BET-dependent AML cell-lines MV4-11 and HL60.

12 – NF-κB Luciferase Assay

Luciferase experiments were performed in human HEK293T cells seeded in a 24-well plate at 100 000 cells in 500 µl media, and transfected with pcDNA5 FRT/TO plasmids encoding WT and mutant GFP-BRD4 chimeras, and pBABE NF-κB-RE/*luc2P* reporter plasmid. Cells were transfected using Lipofectamine™ LTX (Invitrogen) with PLUS™ reagent, with each well receiving 0.5 µg of each plasmid, 2.5 µl LTX and 0.5 µl PLUS reagent. The next day cells were treated with compounds, giving a final concentration of 1 µM compound and 0.01% DMSO. 6 hours later cells were washed with PBS, lysed with passive lysis buffer (Promega) and stored at -20°C.

Luciferase activity was measured suing the Promega luciferase assay. In a black 384-well plate 3µl of clarified lysate was mixed with 15µl luciferase assay reagent and after 15 minutes luminescence was measured in a Pherastar FS. Differences in cell numbers were controlled for by measuring the protein content of each lysate using the Pierce™ Coomassie (Bradford) assay. Each experiment was run twice, on separate days, with each experiment containing 3 technical replicates.

Chemical Synthesis

General information:

NMR spectra were recorded on a Bruker 500 Ultrashield or a Bruker Ascend 400. Chemical shifts are quoted in ppm and referenced to the residual solvent signals: ^{1}H δ = 7.26 ($CDCl_3$), ^{13}C δ = 77.16 ($CDCl_3$). High Resolution Mass Spectra (HRMS) were recorded on a Bruker micrOTOF. All chemicals, unless otherwise stated are commercially available and used without further purification. Micro wave reactions were performed in Biotage Initiator. Flash column chromatography was performed using a Teledyne Isco Combiflash Rf or Rf200i. Prepacked columns RediSep Rf Normal Phase Disposable Columns were used. Preparative HPLC was performed on a Waters mass directed HPLC with a Waters X-Bridge C18 column (100 mm x 19 mm; 5 µm particle size). Separation of the diastereomers was achieved with a gradient of 5 % to 95 % acetonitrile in water with 0.1 % formic acid in the aqueous phase.

Compounds **1**,[2] I-Bet762,[2] and **2**[3] were prepared according to literature procedures. **3**,[4] and **4**[4] were prepared according to previous work in our group.

General procedure for the alkylation in α-positon:

Compound **1** (200 mg, 487 µmol, 1 eq.) or **2** (200 mg, 487 µmol, 1 eq.) was dissolved in anhydrous tetrahydrofuran (5 ml in the case of **1** and 10 ml in the case of **2**). This solution was then added drop wise to a solution of potassium hexamethyldisilazane (1.17 ml of a 0.5 M solution in toluene, 584 µmol, 1.2 eq.) in tetrahydrofuran (5 ml) at -80 °C under an atmosphere of nitrogen. After 1 h at this temperature the corresponding alkyl iodide (584 µmol, 1.2 eq.) was added drop wise. The reaction mixture was warmed to 25 °C over 18 h and a few drops of acetic acid were then added to quench the reaction. The solvent was removed and the residue purified by flash column chromatography using a linear gradient from 10 % to 60 % acetone in heptane. For isomerization, the alkylated compound, together with freshly prepared sodium methoxide (10 eq.), was dissolved in methanol (2 ml) and heated to 120 °C for 40 min in a microwave reactor. The reaction mixture was acidified with aqueous hydrochloric acid (1 M), diluted with water and extracted three times with dichloromethane. The combined organic phases were dried over magnesium sulphate and evaporated to dryness. Separation of the diastereoisomers was achieved as described above.

(+-)methyl (R)-2-((S)-6-(4-chlorophenyl)-8-methoxy-1-methyl-4H-benzo[f][1,2,4]triazolo[4,3-a][1,4]diazepin-4-yl)pent-4-enoate (**5**).

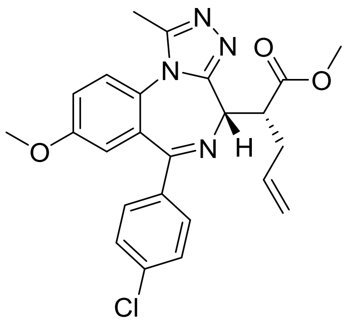

Yield: 32.3 mg (15 %); ^{1}H-NMR ($CDCl_3$, 500 MHz) δ 2.40-2.46 (m, 1 H), 2.60 (s, 3 H), 2.88-2.93 (m, 1 H), 3.80 (s, 3 H), 3.81 (s, 3 H), 4.12-4.16 (m, 1 H), 4.27 (d, 1 H, *J*(H,H)= 11.0 Hz), 4.99-5.06 (m, 2 H), 5.82-5.90 (m, 1 H), 6.87 (d, 1 H, *J*(H,H)= 2.90 Hz), 7.21 (dd, 1 H, *J*(H,H)= 2.90 Hz, *J*(H,H)= 8.90 Hz), 7.30-7.33 (m, 2 H), 7.39-7.43 (m, 3 H); ^{13}C-NMR ($CDCl_3$, 126 MHz) δ 12.1, 34.2, 47.8, 51.5, 55.8, 58.4, 115.8, 117.2, 117.9, 124.8, 126.4, 128.5, 129.9, 130.7, 134.4, 137.0, 150.4, 154.9, 158.0, 165.5, 174.7; HRMS m/z calc. for $C_{24}H_{24}ClN_4O_3$ [M+H$^+$] 451.1531, found 451.1523.

(+-)methyl (R)-2-((S)-6-(4-chlorophenyl)-8-methoxy-1-methyl-4H-benzo[f][1,2,4]triazolo[4,3-a][1,4]diazepin-4-yl)pentanoate (**6**).

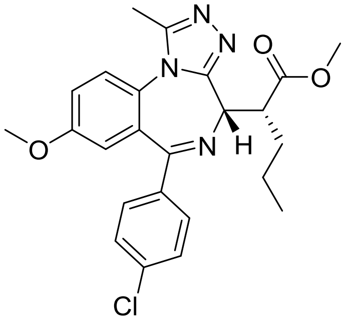

Yield: 28.6 mg (12 %); ^{1}H-NMR (400 MHz, $CDCl_3$) δ 0.92 (t, *J*(H,H)= 7.3 Hz, 3H), 1.35 (m, 1H), 1.53 (m, 2H), 2.06 (m, 1H), 2.59 (s, 3H), 3.80 (s, 3H), 3.83 (s, 3H), 4.05 (m, 1H), 4.22 (d, *J*(H,H)= 11.1 Hz, 1H), 6.86 (d, *J*(H,H)= 2.9 Hz, 1H), 7.21 (dd, *J*(H,H)= 8.9, 2.9 Hz, 1H), 7.31 (m, 2H), 7.40 (m, 3H); ^{13}C-NMR (100 MHz, $CDCl_3$) δ 12.1, 14.0, 20.6, 32.1, 48.1, 51.6, 55.8, 59.1, 115.7, 117.9, 124.8, 126.4, 128.5, 129.9, 130.7, 136.9 (2C), 150.4, 155.1, 158.0, 165.5, 175.7; HRMS m/z calc. for $C_{24}H_{26}ClN_4O_3$ $[M+H]^+$ 453.1693, found: 453.1678.

(+-) methyl (R)-2-((S)-6-(4-chlorophenyl)-9-methoxy-1-methyl-4H-benzo[f][1,2,4]triazolo[4,3-a][1,4]diazepin-4-yl)propanoate (**7**).

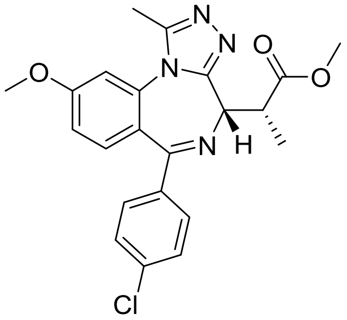

Yield: 35.9 mg (17 %); ^{1}H-NMR ($CDCl_3$, 400 MHz) δ 1.49 (d, 3 H, *J*(H,H)= 6.64 Hz), 2.64 (s, 3 H), 3.82 (s, 3 H), 3.95 (s, 3 H), 4.05-4.11 (m, 1 H), 4.23 (d, 1 H, *J*(H,H)= 11.04 Hz), 6.94 (s, 1 H), 6.98 (d, 1 H, *J*(H,H)= 8.96 Hz), 7.31 (d, 2 H, *J*(H,H)= 7.40 Hz), 7.35-7.39 (m, 3 H); ^{13}C-NMR ($CDCl_3$, 101 MHz) δ 12.3, 15.3, 42.4, 51.8, 55.9, 59.6, 109.4, 112.7, 121.4, 128.4, 130.8, 133.4, 134.7, 136.7, 137.4, 150.2, 154.9, 161.6, 165.7, 176.1; HRMS m/z calc. for $C_{22}H_{22}ClN_4O_3$ [M+H$^+$] 425.1375, found 425.1381.

(+-) Methyl (S*)-2-((S*)-6-(4-chlorophenyl)-9-methoxy-1-methyl-4H-benzo[f][1,2,4]triazolo[4,3-a][1,4]diazepin-4-yl)propanoate (S*, S*) 7

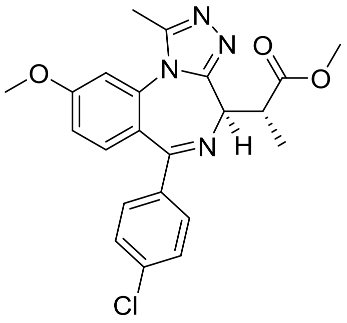

^{1}H NMR (400 MHz, CDCl3) δ: 1.60 (d, J=7.1 Hz, 3H), 2.63 (s, 3H), 3.72 (s, 3H), 3.86- 3.92 (m, 3H), 3.94 (s, 3H), 4.27 (d, J=10.3 Hz, 1H), 6.93 (d, J=2.4 Hz, 1H), 6.98 (dd, J=2.6, 8.8 Hz, 1H),7.32-7.38 (m, 3H), 7.49 (d, J=8.1 Hz, 2H). 13C-NMR (100 MHz, CDCl3) δ: 12.4, 15.5, 41.1, 52.0, 55.9, 57.8, 76.7, 77.0, 77.3, 109.3, 112.9, 121.4, 128.5, 130.8, 133.1, 135.1, 136.8, 137.7, 150.0, 156.1, 161.6, 166.5, 176.1; HRMS m/z calc. for C22H22ClN4O3 [M+H+] 425.1375, found 425.1388.

(+-) methyl (R)-2-((S)-6-(4-chlorophenyl)-9-methoxy-1-methyl-4H-benzo[f][1,2,4]triazolo[4,3-a][1,4]diazepin-4-yl)butanoate (**8**).

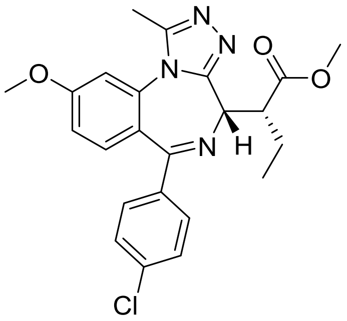

Yield: 25.6 mg (12 %); ^{1}H-NMR ($CDCl_3$, 400 MHz) δ 1.01 (t, 3 H, *J*(H,H)= 7.28 Hz), 1.57-1.68 (m, 1 H), 2.12-2.21 (m, 1 H), 2.63 (s, 3 H), 3.84 (s, 3 H) 3.95 (s, 3 H), 4.00 (dd, 1 H, *J*(H,H)= 2.80 Hz, *J*(H,H)= 11.2 Hz), 4.23 (d, 1 H, *J*(H,H)= 10.9 Hz), 6.94 (s, 1 H) 6.98 (d, 1 H, *J*(H,H)= 8.48 Hz), 7.30 (d, 2 H, *J*(H,H)= 7.68 Hz), 7.34-7.38 (m, 3 H); ^{13}C-NMR ($CDCl_3$, 101 MHz) δ 11.6, 12.4, 23.2, 49.7, 51.6, 55.9, 58.8, 109.4, 112.7, 121.4, 128.4, 130.8, 133.4, 134.7, 136.7, 137.4, 150.2, 155.1, 161.6, 165.8, 175.5; HRMS m/z calc. for $C_{23}H_{24}ClN_4O_3$ [M+H$^+$] 439.1531, found 439.1513.

(+-)methyl (R)-2-((S)-6-(4-chlorophenyl)-9-methoxy-1-methyl-4H-benzo[f][1,2,4]triazolo[4,3-a][1,4]diazepin-4-yl)pent-4-enoate (**9**).

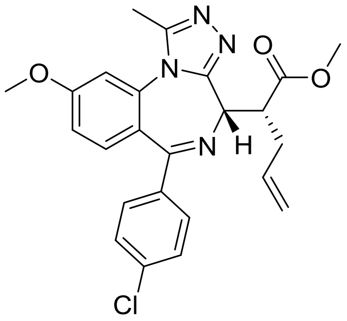

Yield: 27.3 mg (12 %); ^{1}H-NMR ($CDCl_3$, 400 MHz) δ 2.38-2.46 (m, 1 H), 2.64 (s, 3 H), 2.88-2.94 (m, 1 H), 3.80 (s, 3 H), 3.95 (s, 3 H), 4.11-4.17 (m, 1 H), 4.27 (d, 1 H, *J*(H,H)= 10.9 Hz), 4.99-5.07 (m, 2 H), 5.82-5.92 (m, 1 H), 6.93 (d, 1 H, *J*(H,H)= 2.44 Hz), 6.98 (dd, 1 H, *J*(H,H)= 2.48 Hz, *J*(H,H)= 8.80 Hz), 7.29-7.32 (m, 2 H), 7.34-7.39 (m, 3 H); ^{13}C-NMR ($CDCl_3$, 101 MHz) δ 12.4, 34.2, 47.9, 51.6, 55.9, 58.3, 109.4, 112.7, 117.2, 121.3, 128.4, 130.8, 133.4, 134.5, 134.7, 136.8, 137.3, 150.3, 154.8, 161.6, 165.9, 174.7; HRMS m/z calc. for $C_{24}H_{24}ClN_4O_3$ [M+H$^+$] 451.1531, found 451.1540.

General procedure for Amide formation:

The mixture of diasteromers of the ester compounds (100 µmol, 1 eq.) were hydrolyzed in methanol (0.5 ml) and aqueous sodium hydroxide (0.5 ml, 1 M in water) by heating to 100 °C for 30 min in a microwave oven. After quenching with aqueous hydrochloric acid (1 M) the reaction mixture was extracted three times with dichloromethane. The combined organic phases were dried over magnesium sulfate and evaporated to dryness. To this end, the obtained free carboxylic acid was dissolved in dichloromethane, the corresponding amine (150 µmol, 1.5 eq.), HATU (57.0 mg, 150 µmol, 1.5 eq.) and *N,N*-diisopropylethylamine (69.9 µl, 400µmol, 4 eq.) were added and the reaction mixture stirred at 25 °C for 2 h. The solvent was removed and the residue subject to flash column chromatography before the diastereomers were separated as described above.

(+-) (R)-2-((S)-6-(4-chlorophenyl)-8-methoxy-1-methyl-4H-benzo[f][1,2,4]triazolo[4,3-a][1,4]diazepin-4-yl)-N-ethylpropanamide (**16**).

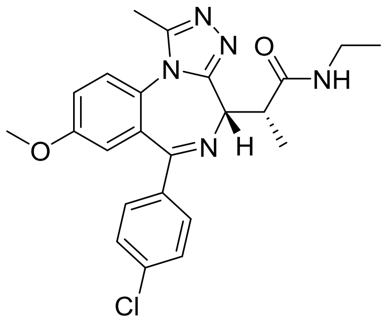

Yield: 13.2 mg (30 %); ^{1}H-NMR ($CDCl_3$, 500 MHz) δ 1.25 (t, 3 H, *J*(H,H)= 7.25 Hz), 1.44 (d, 3 H, *J*(H,H)= 6.75 Hz), 2.59 (s, 3 H), 3.33-3.50 (m, 2 H), 3.63-3.69 (m, 1 H), 3.79 (s, 3 H), 4.24 (d, 1 H, *J*(H,H)= 9.80 Hz), 6.25 (t, 1 H, *J*(H,H)= 5.49 Hz), 6.85 (d, 1 H, *J*(H,H)= 2.90 Hz), 7.20 (dd, 1 H, *J*(H,H)= 2.90 Hz, *J*(H,H)= 8.90 Hz), 7.29-7.32 (m, 2 H), 7.39 (d, 1 H, *J*(H,H)= 8.85 Hz), 7.43-7.46 (m, 2 H); ^{13}C-NMR ($CDCl_3$, 126 MHz) δ 12.1, 15.0, 15.6, 34.4, 43.6, 55.8, 59.7, 115.6, 118.1, 124.8, 126.4, 128.4, 130.0, 136.9, 137.1, 150.3, 155.6, 158.0, 165.5, 174.4; HRMS m/z calc. for $C_{23}H_{25}ClN_5O_2$ [M+H$^+$] 438.1691, found 438.1675.

(+-) (R)-2-((S)-6-(4-chlorophenyl)-8-methoxy-1-methyl-4H-benzo[f][1,2,4]triazolo[4,3-a][1,4]diazepin-4-yl)-N-ethylbutanamide (**17**).

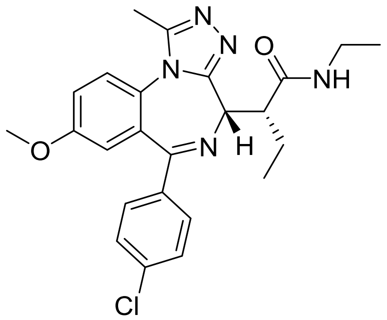

Yield: 11.7 mg (26 %); ^{1}H-NMR ($CDCl_3$, 500 MHz) δ 1.03 (t, 3 H, *J*(H,H)= 7.35 Hz), 1.27 (t, 3 H, *J*(H,H)= 7.25 Hz), 1.64-1.74 (m, 1 H), 2.01-2.09 (m, 1 H), 2.59 (s, 3 H), 3.42-3.49 (m, 3 H), 3.79 (s, 3 H), 4.23 (d, 1 H, *J*(H,H)= 10.0 Hz), 6.17 (s, 1 H), 6.85 (d, 1 H, *J*(H,H)= 2.85 Hz), 7.20 (dd, 1 H, *J*(H,H)= 2.90 Hz, *J*(H,H)= 10.4 Hz), 7.30-7.21 (m, 2 H), 7.38 (d, 1 H, *J*(H,H)= 8.90 Hz), 7.42-7.45 (m, 2 H); ^{13}C-NMR ($CDCl_3$, 126 MHz) δ 11.9, 12.1, 15.2, 22.9, 34.4, 51.3, 55.8, 59.0, 115.6, 118.1, 124.8, 126.5, 128.4, 130.0, 130.8, 136.9, 137.1, 150.3, 155.7, 158.0, 165.4, 173.5; HRMS m/z calc. for $C_{24}H_{26}ClN_5O_2$ [M+H$^+$]452.1848, found 452.1839.

(+-) (R)-2-((S)-6-(4-chlorophenyl)-8-methoxy-1-methyl-4H-benzo[f][1,2,4]triazolo[4,3-a][1,4]diazepin-4-yl)-N-ethylpent-4-enamide (**18**).

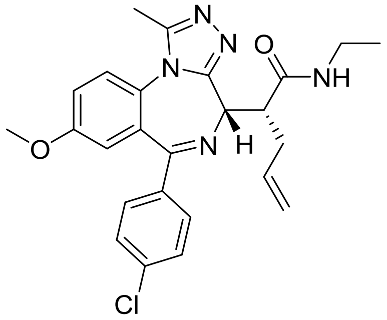

Yield: 14.7 mg (32 %); ^{1}H-NMR ($CDCl_3$, 400 MHz) δ 1.19 (t, 3 H, *J*(H,H)= 7.20 Hz), 2.48-2.56 (m, 1 H), 2.62 (s, 3 H), 2.70-2.76 (m, 1 H), 3.32-3.44 (m, 2 H), 3.52-3.58 (m, 1 H), 3.80 (s, 3 H), 4.32 (d, 1 H, *J*(H,H)= 8.48 Hz), 5.02 (d, 1 H, *J*(H,H)= 10.3 Hz), 5.10 (d, 1 H, *J*(H,H)= 17.0 Hz), 5.82-5.92 (m, 1 H), 6.60 (s, 1 H), 6.86 (d, 1 H, *J*(H,H)= 2.84 Hz), 7.23 (dd, 1 H, *J*(H,H)= 2.84 Hz, *J*(H,H)= 8.96 Hz), 7.33-7.35 (m, 2 H), 7.44-7.48 (m, 3 H); ^{13}C-NMR ($CDCl_3$, 101 MHz) δ 11.8, 14.9, 34.4, 48.5, 55.9, 57.7, 116.1, 117.5, 118.1, 125.2, 125.6, 128.5, 129.9, 130.9, 134.8, 136.8, 137.2, 150.8, 155.3, 158.4, 166.4, 172.9; HRMS m/z calc. for $C_{25}H_{27}ClN_5O_2$ [M+H$^+$] 464.1848, found 464.1840.

(+-) (R)-2-((S)-6-(4-chlorophenyl)-8-methoxy-1-methyl-4H-benzo[f][1,2,4]triazolo[4,3-a][1,4]diazepin-4-yl)-N,N-diethylpropanamide (19)

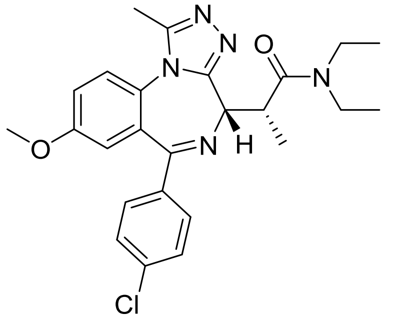

Yield: 17.3 mg (37 %); ^{1}H-NMR ($CDCl_3$, 400 MHz) δ 1.24 (t, 3 H, *J*(H,H)= 7.24 Hz), 1.33 (t, 3 H, *J*(H,H)= 6.92 Hz), 1.41 (d, 3 H, *J*(H,H)= 6.64 Hz), 2.65 (s, 3 H), 3.37-3.45 (m, 1 H), 3.50-3.74 (m, 3 H), 3.80 (s, 3 H), 4.21-4.29 (m, 1 H), 4.40 (d, 1 H, *J*(H,H)= 10.6 Hz), 6.88 (s, 1 H), 7.21-7.29 (m, 1 H), 7.30 (d, 2 H), 7.41-7.46 (m, 3 H); ^{13}C-NMR ($CDCl_3$, 101 MHz) δ 11.8, 13.4, 15.0, 15.6, 38.0, 40.7, 42.5, 55.9, 60.4, 115.7, 118.1, 125.0, 125.8, 128.3, 130.0, 130.8, 136.9, 137.0, 150.7, 155.8, 158.3, 165.1, 174.3; HRMS m/z calc. for $C_{25}H_{29}ClN_5O_2$ [M+H$^+$] 466.2004, found 466.1997.

(+-) (R)-2-((S)-6-(4-chlorophenyl)-8-methoxy-1-methyl-4H-benzo[f][1,2,4]triazolo[4,3-a][1,4]diazepin-4-yl)-N,N-diethylbutanamide (20)

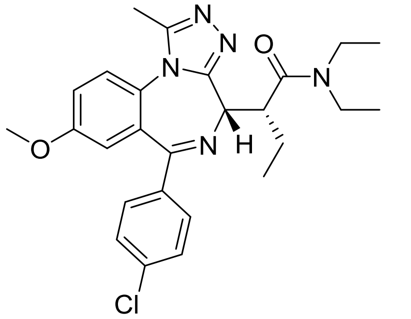

Yield: 15.8 mg (33 %); ^{1}H-NMR ($CDCl_3$, 400 MHz) δ 1.00 (t, 3 H, *J*(H,H)= 7.28 Hz), 1.26 (t, 3 H, *J*(H,H)= 7.00 Hz), 1.33 (t, 3 H, *J*(H,H)= 6.84 Hz), 1.65-1.74 (m, 1 H), 2.04-2.11 (m, 1 H), 2.62 (s, 3 H), 3.50-3.76 (m, 4 H), 3.80 (s, 3 H), 4.11-4.23 (m, 1 H), 4.31 (d, 1 H, *J*(H,H)= 10.5 Hz), 6.86 (s, 1 H), 7.19-7.22 (m, 1 H), 7.29 (d, 2 H, *J*(H,H)= 7.64 Hz), 7.40-7.43 (m, 3 H); ^{13}C-NMR ($CDCl_3$, 101 MHz) δ 11.6, 11.9, 13.4, 14.8, 40.8, 42.5, 44.4, 55.9, 59.9, 115.7, 118.1, 124.9, 126.0, 128.3, 129.9, 130.8, 136.8, 137.1, 150.6, 155.9, 158.2, 165.2, 173.5; HRMS m/z calc. for $C_{26}H_{31}ClN_5O_2$ [M+H$^+$] 480.2161, found 480.2171.

(+-) (R)-2-((S)-6-(4-chlorophenyl)-9-methoxy-1-methyl-4H-benzo[f][1,2,4]triazolo[4,3-a][1,4]diazepin-4-yl)-N-ethylpropanamide (**21**).

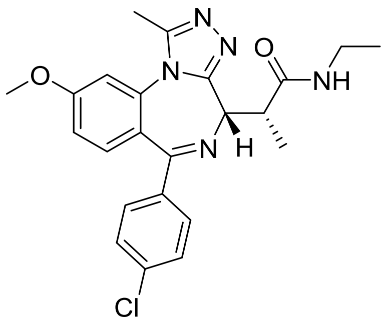

Yield: 12.6 mg (29 %); ^{1}H-NMR ($CDCl_3$, 400 MHz) δ 1.24 (t, 3 H, *J*(H,H)= 7.20 Hz), 1.44 (d, 3 H, *J*(H,H)= 6.56 Hz), 2.62 (s, 3 H), 3.32-3.50 (m, 2 H), 3.62-3.70 (m, 1 H), 3.93 (s, 3 H), 4.23 (d, 1 H, *J*(H,H)= 9.64 Hz), 6.28 (s, 1 H), 6.93-6.97 (m, 2 H), 7.28-7.34 (m, 3 H), 7.40 (d, 2 H, *J*(H,H)= 7.62 Hz); ^{13}C-NMR ($CDCl_3$, 101 MHz) δ 12.3, 15.0, 15.6, 34.4, 43.6, 55.9, 59.6, 109.4, 112.7, 121.4, 128.3, 130.8, 133.4, 134.7, 136.7, 137.5, 150.2, 155.5, 161.5, 165.8, 174.4; HRMS m/z calc. for $C_{23}H_{25}ClN_5O_2$ [M+H$^+$] 438.1691, found 438.1684.

(+-)(R)-2-((S)-6-(4-chlorophenyl)-9-methoxy-1-methyl-4H-benzo[f][1,2,4]triazolo[4,3-a][1,4]diazepin-4-yl)-N-ethylbutanamide (22)

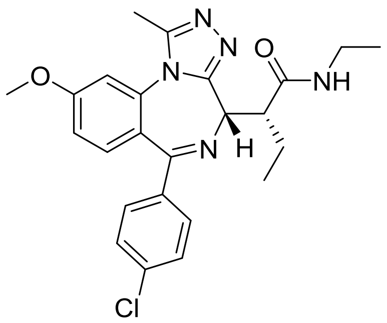

Yield: 9.5 mg (21 %); ^{1}H-NMR ($CDCl_3$, 400 MHz) δ 1.03 (t, 3 H, *J*(H,H)= 7.24 Hz), 1.24 (t, 3 H, *J*(H,H)= 7.24 Hz), 1.70-1.78 (m, 1 H), 1.96-2.06 (m, 1 H), 2.64 (s, 3 H), 3.41-3.48 (m, 3 H), 3.94 (s, 3 H), 4.26 (d, 1 H, *J*(H,H)= 9.32 Hz), 6.43 (s, 1 H), 6.97-6.99 (m, 2 H), 7.31-7.34 (m, 3 H), 7.42 (d, 2 H, *J*(H,H)= 7.94 Hz); ^{13}C-NMR ($CDCl_3$, 101 MHz) δ 11.9, 12.2, 15.0, 23.2, 34.4, 50.9, 56.0, 58.4, 109.4, 113.3, 121.2, 128.4, 130.9, 133.5, 134.4, 137.0, 137.3, 150.5, 155.5, 161.8, 173.7; HRMS m/z calc. for $C_{24}H_{27}ClN_5O_2$ [M+H$^+$] 452.1848, found 452.1855.

General procedure for *tert*-butyl ester formation:

The mixture of diasteroisomers of the alkylated methyl-esters (100 µmol, 1 eq.) were hydrolyzed in methanol (0.5 ml) and aqueous sodium hydroxide (0.5 ml, 1 M in water) by heating to 100 °C for 30 min in a microwave reactor. After quenching with aqueous hydrochloric acid (1 M) the reaction mixture was extracted three times with dichloromethane. The combined organic phases were dried over magnesium sulfate and evaporated to dryness. The crude was dissolved in anhydrous dichloromethane and *tert*-butyl trichloroacetimidate (200 µmol, 2 eq.) was added. After addition of boron trifluoride-diethyl ether complex (5 µmol, 0.05 eq.) the reaction mixture was stirred at 25 °C for 18 h. The solvent was removed and the residue subject to flash column chromatography before the diastereoisomers were separated as described above.

tert-butyl (R)-2-((S)-6-(4-chlorophenyl)-8-methoxy-1-methyl-4H-benzo[f][1,2,4]triazolo[4,3-a][1,4]diazepin-4-yl)butanoate (**23**).

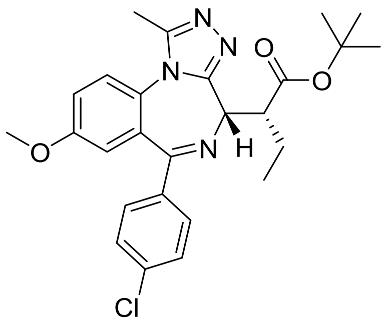

Yield: 3.21 mg (7 %); ^{1}H-NMR ($CDCl_3$, 400 MHz) δ 1.04 (t, 3 H, *J*(H,H)= 7.40), 1.56 (s, 9 H), 1.58-1.63 (m, 1 H), 2.11-2.18 (m, 1 H), 2.59 (s, 3 H), 3.81 (s, 3 H), 3.84-3.90 (m, 1 H), 4.19 (d, 1 H, *J*(H,H)= 11.1), 6.88 (d, 1 H, *J*(H,H)= 2.7), 7.20 (dd, 1 H, *J*(H,H)= 2.9, *J*(H,H)= 8.98), 7.30-7.32 (m, 2H), 7.38-7.40 (m, 1 H), 7.46-7.48 (m, 2 H); ^{13}C-NMR ($CDCl_3$, 101 MHz) δ 11.6, 12.3, 23.5, 28.5, 50.2, 56.0, 59.1, 80.7, 115.7, 118.0, 124.9, 126.6, 128.5, 128.8, 130.2, 130.9, 137.0, 137.2, 150.4, 155.5, 158.1, 165.2, 174.2; HRMS m/z calc. for $C_{26}H_{30}ClN_4O_3$ [M+H$^+$] 481.2001, found 481.2009.

tert-butyl (R)-2-((S)-6-(4-chlorophenyl)-8-methoxy-1-methyl-4H-benzo[f][1,2,4]triazolo[4,3-a][1,4]diazepin-4-yl)pent-4-enoate (**24**).

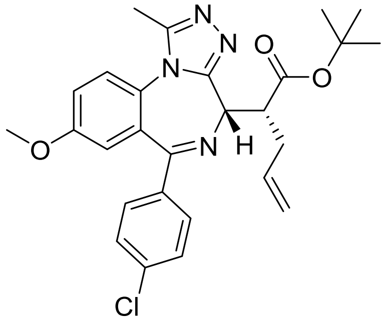

Yield: 5.20 mg (11 %); ^{1}H-NMR ($CDCl_3$, 400 MHz) δ 1.52 (s, 9 H), 2.35-2.43 (m, 1 H), 2.60 (s, 3 H), 2.85-2.90 (m, 1 H), 3.81 (s, 3 H), 3.99 (dt, 1 H, *J*(H,H)= 3.7, *J*(H,H)= 10.5), 4.22 (d, 1 H, *J*(H,H)= 11.0), 4.98-5.05 (m, 2 H), 5.83-5.93 (m, 1 H), 6.88 (d, 1 H, *J*(H,H)= 2.8), 7.20 (dd, 1 H, *J*(H,H)= 2.8, *J*(H,H)= 8.8), 7.30-7.32 (m, 2 H), 7.38-7.40 (m, 1 H), 7.46-7.48 (m, 2 H); ^{13}C-NMR ($CDCl_3$, 101 MHz) δ 12.3, 28.4, 34.7, 48.3, 56.0, 58.6, 81.0, 115.8, 117.2, 118.0, 124.9, 126.6, 128.6, 130.1, 130.9, 134.7, 137.0, 137.1, 150.4, 155.2, 158.1, 165.3, 173.4; HRMS m/z calc. for $C_{27}H_{30}ClN_4O_3$ [M+H$^+$] 493.2001, found 493.2021.

tert-butyl (R)-2-((S)-6-(4-chlorophenyl)-9-methoxy-1-methyl-4H-benzo[f][1,2,4]triazolo[4,3-a][1,4]diazepin-4-yl)butanoate (25)

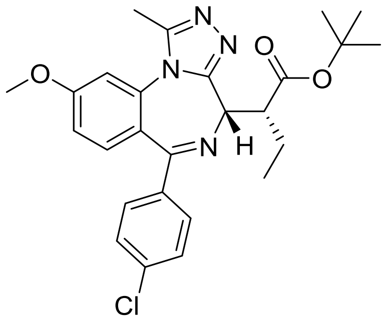

^{1}H NMR (400 MHz, CDCl3) 1.04 (t, J=7.6 Hz, 3H), 1.55-1. 63 (m, 11H), 2.11-2.18 (m, 1H), 2.62 (s, 3H), 3.81-3.87 (m, 1H), 3.95 (s, 3H), 4.18 (d, J=11.1 Hz, 1H), 6.93 (d, J=2.2 Hz, 1H), 6.98 (dd, J=2.5, 8.7 Hz, 1H), 7.30 (d, J=8.5 Hz, 2H), 7.36 (d, J=8.7 Hz, 1H), 7.43 (d, J=8.7 Hz, 2H). 13C-NMR (CDCl3, 500 MHz) 11.5, 12.4, 23.4, 28.3, 50.2, 55.9, 58.9, 80.6, 109.4, 112.7, 121.5, 128.3, 130.9, 133.3, 134.8, 136.7, 137.5, 150.1, 155.3, 161.5, 165.4, 174.0; HRMS m/z calc. for C26H30ClN4O3 [M+H+] 481.2006, found 481.2015

tert-butyl (R)-2-((S)-6-(4-chlorophenyl)-9-methoxy-1-methyl-4H-benzo[f][1,2,4]triazolo[4,3-a][1,4]diazepin-4-yl)pent-4-enoate (26)

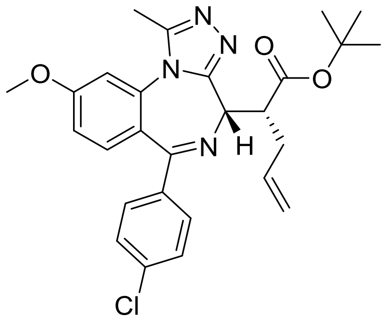

^{1}H NMR (CDCl3, 500 MHz) 1.52 (s, 9H), 2.36-2.43 (m, 1H), 2.64 (s, 3H), 2.86-2.91 (m, 1H), 3.95 (s, 3H), 3.96-4.02 (m, 1H), 4.22 (d, J=11.0 Hz, 1H), 4.98-5.07 (m, 2H), 5.85-5.94 (m, 1H), 6.93 (d, J=2.4 Hz, 1H), 6.98 (dd, J=2.5, 8.8 Hz, 1H), 7.30 (d, J=8.4 Hz, 2H), 7.37 (d, J=8.7 Hz, 1H), 7.43 (d, J=8.7 Hz, 2H). 13C-NMR (CDCl3, 500 MHz) 12.4, 28.3, 34.6, 48.3, 55.9, 58.4, 80.8, 109.4, 112.7, 117.0, 121.5, 128.4, 130.9, 133.3, 134.7, 134.8, 136.7, 137.5, 150.1, 155.1, 161.5, 165.5, 173.2; HRMS m/z calc. for C27H30ClN4O3 [M+H+] 493.2006, found 493.2025

Chiral HPLC separation of compound 7

Racemate 7 was dissolved to 20 mg/mL in ethanol and was then purified by HPLC. Combined fractions of each of UD5_1 and UD5_2 were then evaporated to near dryness using a rotary evaporator, transferred into final vessels with DCM, which was removed under a stream of nitrogen at 40C before being stored in a vacuum oven at 40°C and 5mbar for 16 hours to afford UD5_1 and UD5_2 as off white glasses.

Column Details: Lux A1 (21.2mm x 250mm, 5um)

Column Temperature: Ambient

Flow Rate: 21 mL/min

Detector Wavelength: 220 nm

Injection Volume: 1000 uL (20 mg)

Isocratic Conditions 4:6 HEPT:EtOH (0.1% v/v NH3)A)

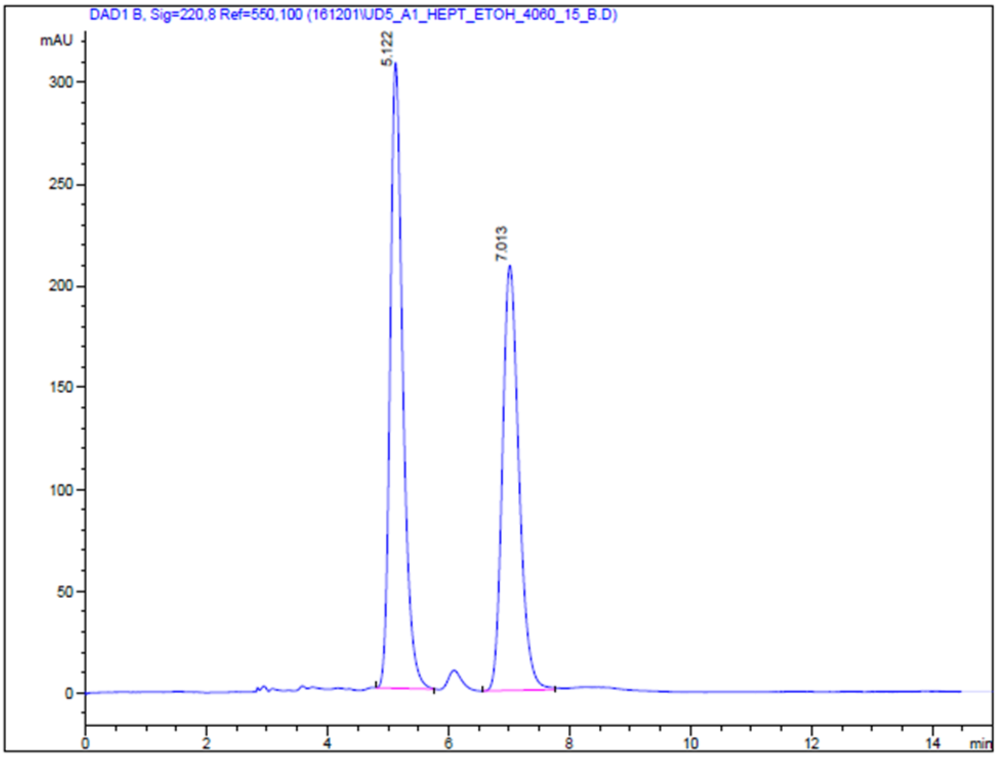

#	Meas. R	Height	Width	Area	Area %	Symmetr
1	5.122	307.488	0.243	4.484e3	53.076	0.730
2	7.013	208.429	0.294	3.965e3	46.924	0.789

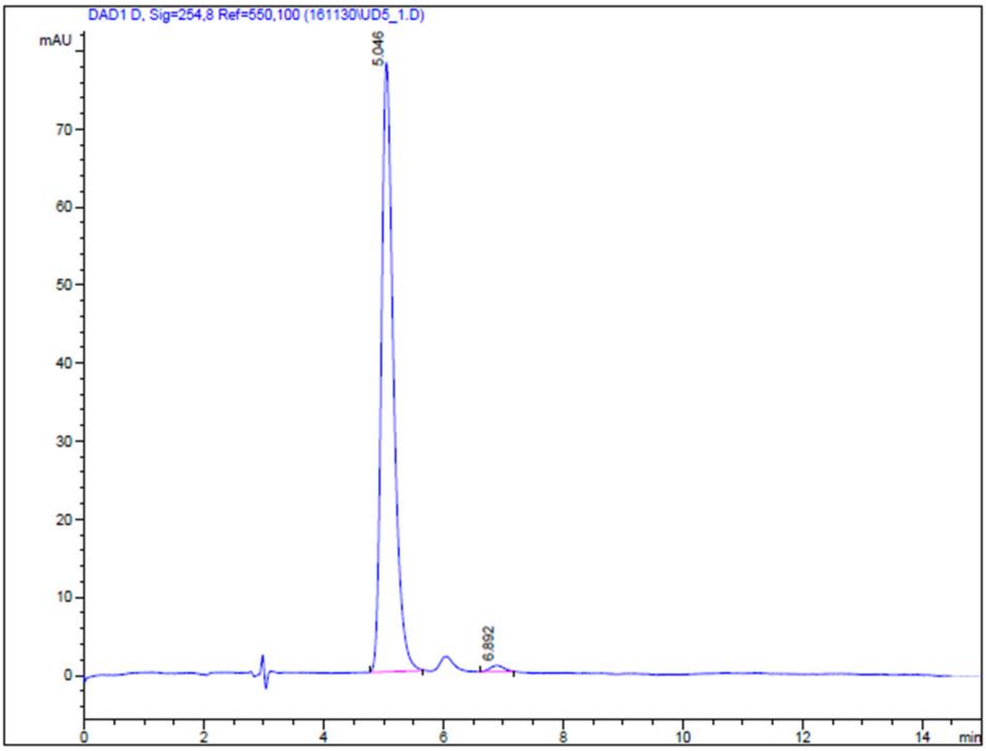

#	Meas. R	Height	Width	Area	Area %	Symmetr
1	5.046	78.080	0.234	1.094e3	98.786	0.692
2	6.892	0.828	0.271	13.447	1.214	0.986

B)

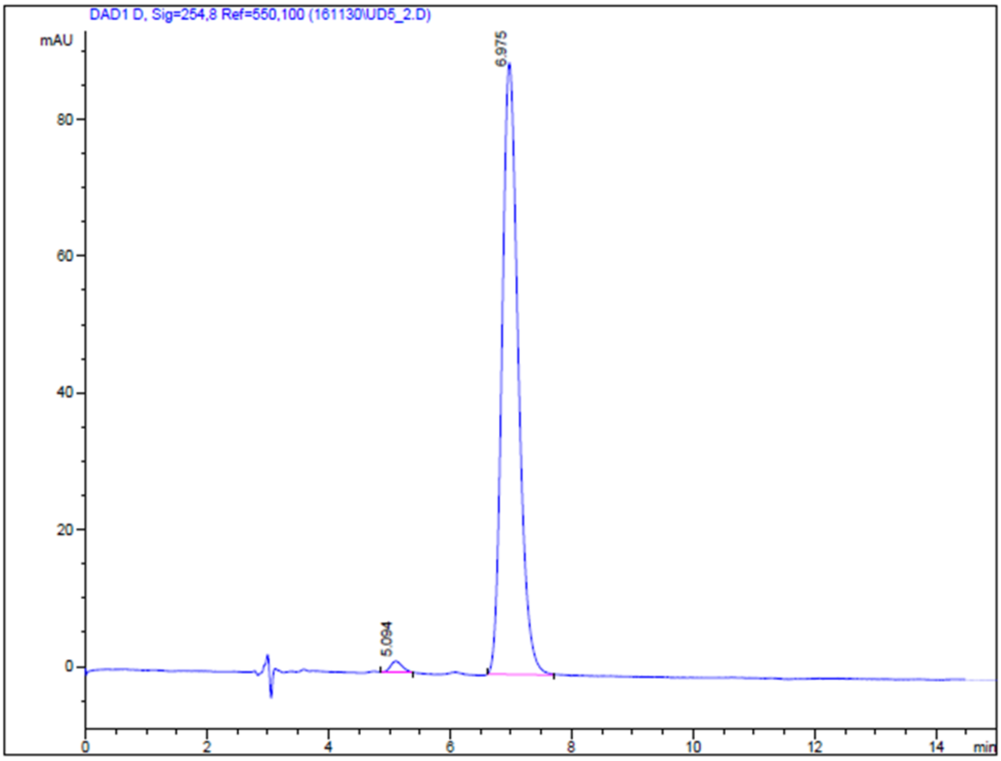

#	Meas. R	Height	Width	Area	Area %	Symmetr
1	5.094	1.621	0.227	22.051	1.314	0.650
2	6.975	88.925	0.297	1.657e3	98.686	0.793

C)

HPLC traces of A) 7 racemate (7), B) (2R, 3S)-7, C) (2S,3R)-7

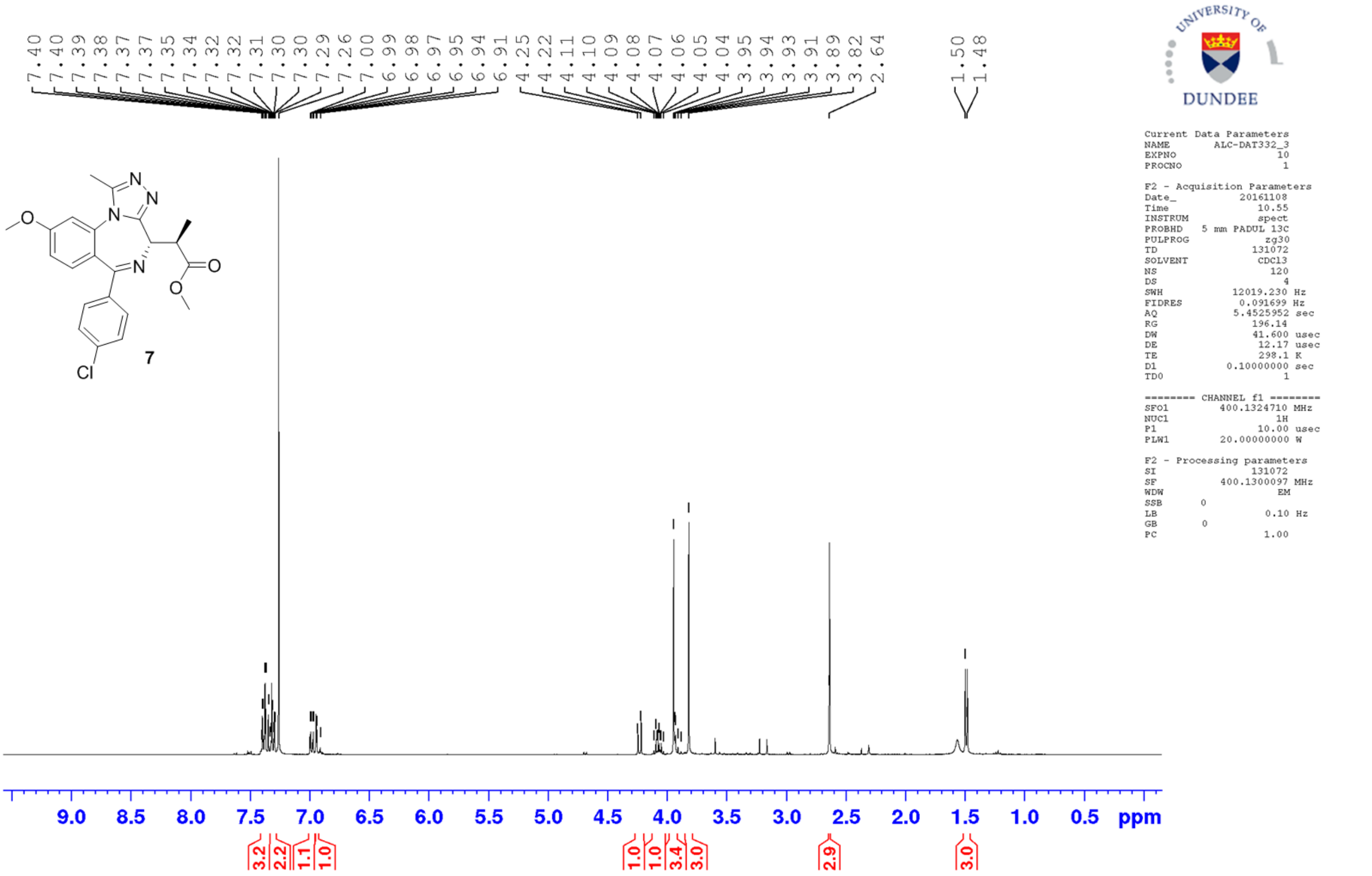

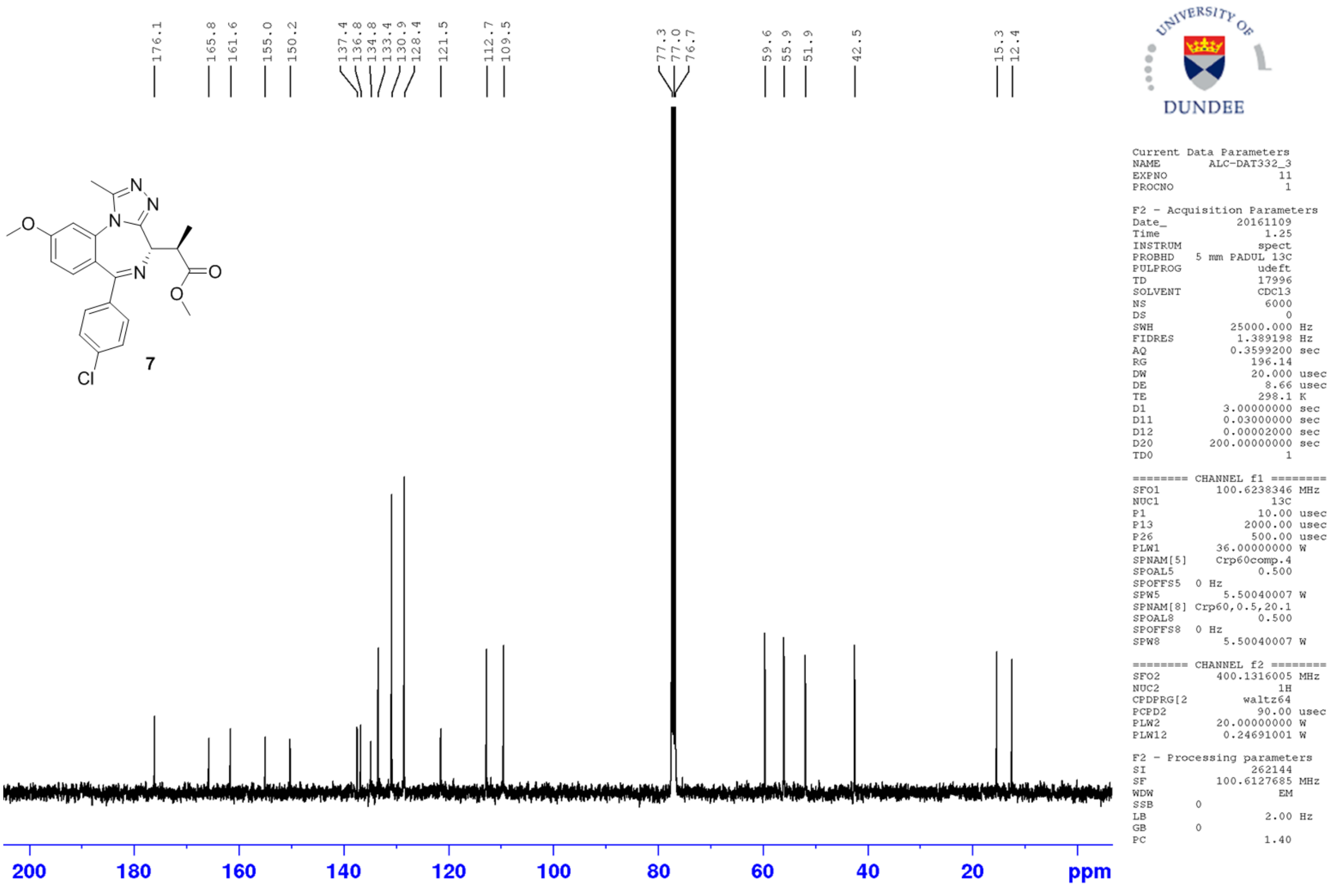

^{1}H and ^{13}C NMR spectra of 7 S*R* diastereomer

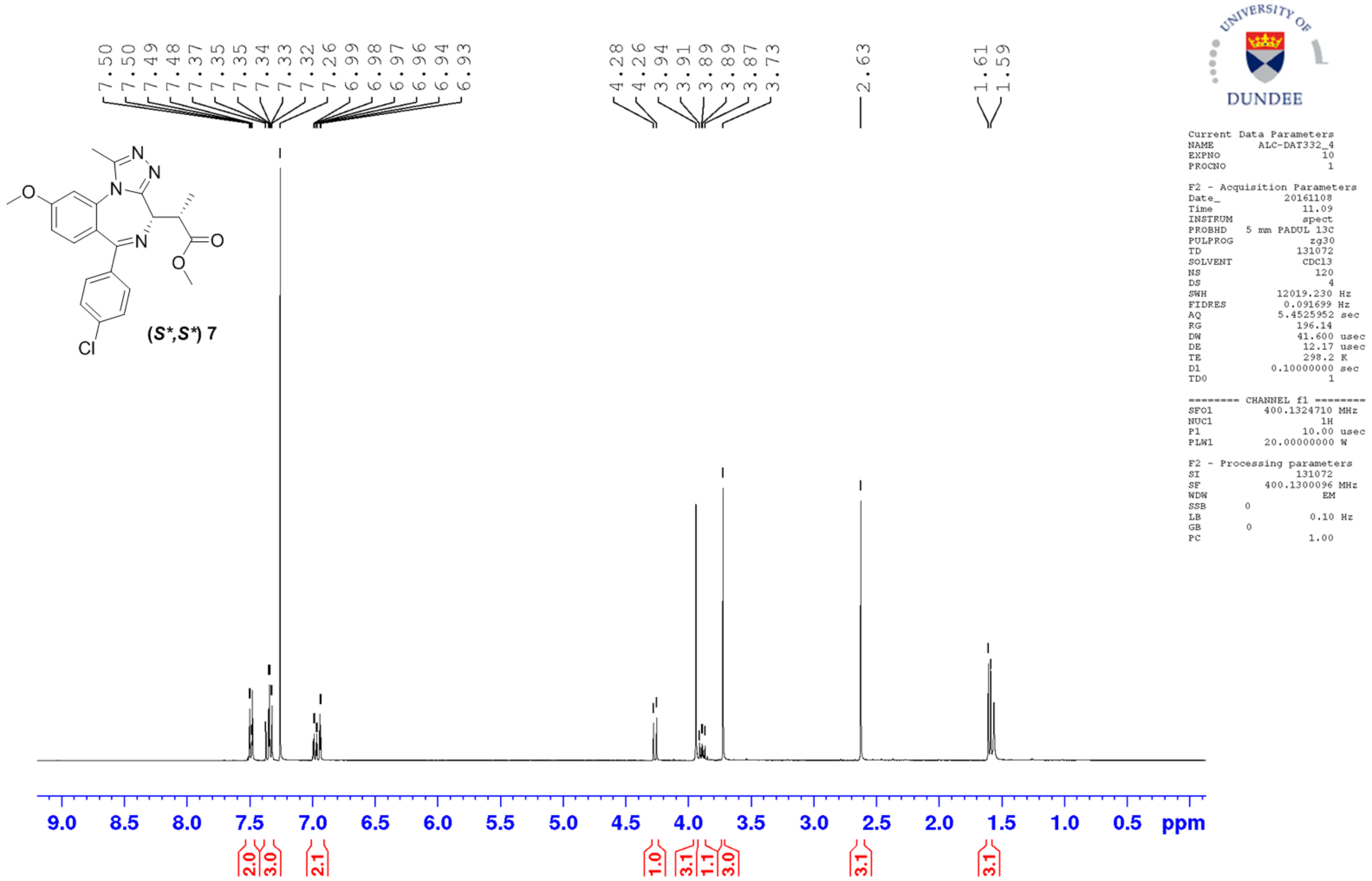

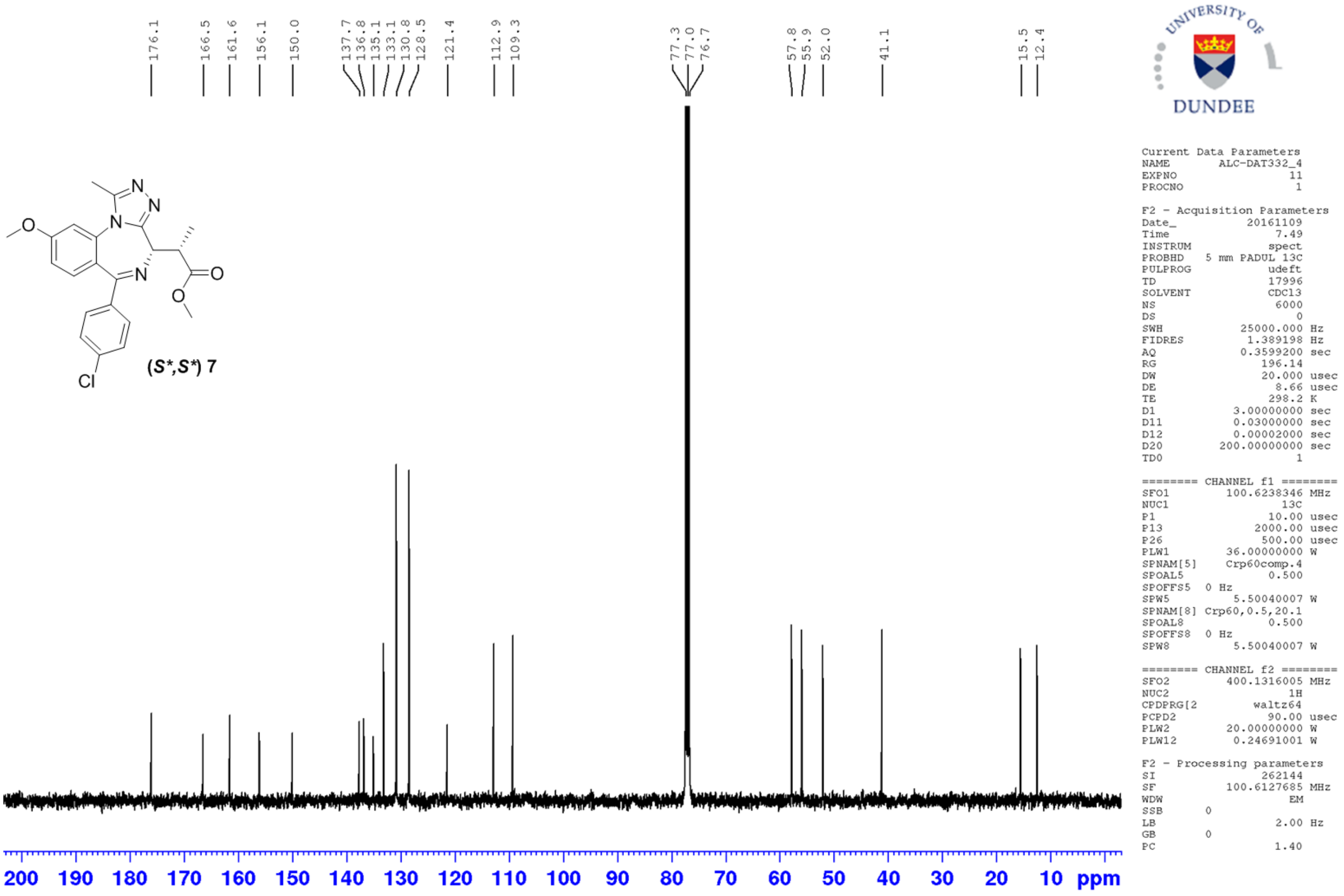

^{1}H and ^{13}C NMR spectra of 7 S*S* diastereomer

Supplemental References

1. F. Gong, L. Y. Chiu, B. Cox, F. Aymard, T. Clouaire, J. W. Leung, M. Cammarata, M. Perez, P. Agarwal, J. S. Brodbelt, G. Legube and K. M. Miller, *Genes Dev*, 2015, 29, 197-211.
2. F. H. Niesen, H. Berglund and M. Vedadi, *Nat Protoc*, 2007, 2, 2212-2221.
3. W. Kabsch, *Acta Crystallogr D Biol Crystallogr*, 2010, 66, 125-132.
4. T. G. Battye, L. Kontogiannis, O. Johnson, H. R. Powell and A. G. Leslie, *Acta Crystallogr D Biol Crystallogr*, 2011, 67, 271-281.
5. P. R. Evans and G. N. Murshudov, *Acta Crystallogr D Biol Crystallogr*, 2013, 69, 1204-1214.
6. M. D. Winn, C. C. Ballard, K. D. Cowtan, E. J. Dodson, P. Emsley, P. R. Evans, R. M. Keegan, E. B. Krissinel, A. G. Leslie, A. McCoy, S. J. McNicholas, G. N. Murshudov, N. S. Pannu, E. A. Potterton, H. R. Powell, R. J. Read, A. Vagin and K. S. Wilson, *Acta Crystallogr D Biol Crystallogr*, 2011, 67, 235-242.
7. A. Vagin and A. Teplyakov, *Acta Crystallogr D Biol Crystallogr*, 2010, 66, 22-25.
8. A. J. McCoy, R. W. Grosse-Kunstleve, P. D. Adams, M. D. Winn, L. C. Storoni and R. J. Read, *J Appl Crystallogr*, 2007, 40, 658-674.
9. P. V. Afonine, R. W. Grosse-Kunstleve, N. Echols, J. J. Headd, N. W. Moriarty, M. Mustyakimov, T. C. Terwilliger, A. Urzhumtsev, P. H. Zwart and P. D. Adams, *Acta Crystallogr D Biol Crystallogr*, 2012, 68, 352-367.
10. P. D. Adams, P. V. Afonine, G. Bunkoczi, V. B. Chen, I. W. Davis, N. Echols, J. J. Headd, L. W. Hung, G. J. Kapral, R. W. Grosse-Kunstleve, A. J. McCoy, N. W. Moriarty, R. Oeffner, R. J. Read, D. C. Richardson, J. S. Richardson, T. C. Terwilliger and P. H. Zwart, *Acta Crystallogr D Biol Crystallogr*, 2010, 66, 213-221.
11. P. Emsley, B. Lohkamp, W. G. Scott and K. Cowtan, *Acta Crystallogr D Biol Crystallogr*, 2010, 66, 486-501.
12. N. W. Moriarty, R. W. Grosse-Kunstleve and P. D. Adams, *Acta Crystallogr D Biol Crystallogr*, 2009, 65, 1074-1080.
13. N. W. Moriarty, E. J. Draizen and P. D. Adams, *Acta Crystallogr D Struct Biol*, 2017, 73, 123-130.
14. A. W. Schuttelkopf and D. M. van Aalten, *Acta Crystallogr D Biol Crystallogr*, 2004, 60, 1355-1363.
15. V. B. Chen, W. B. Arendall, 3rd, J. J. Headd, D. A. Keedy, R. M. Immormino, G. J. Kapral, L. W. Murray, J. S. Richardson and D. C. Richardson, *Acta Crystallogr D Biol Crystallogr*, 2010, 66, 12-21.
16. The PyMol Molecular Graphics System Schrodinger, LLC,
